# The Open Pediatric Cancer Project

**DOI:** 10.1093/gigascience/giaf093

**Published:** 2025-09-02

**Authors:** Zhuangzhuang Geng, Eric Wafula, Ryan J Corbett, Yuanchao Zhang, Run Jin, Krutika S Gaonkar, Sangeeta Shukla, Komal S Rathi, Dave Hill, Aditya Lahiri, Daniel P Miller, Alex Sickler, Kelsey Keith, Christopher Blackden, Antonia Chroni, Miguel A Brown, Adam A Kraya, Kaylyn L Clark, Brian R Rood, Adam C Resnick, Nicholas Van Kuren, John M Maris, Alvin Farrel, Mateusz P Koptyra, Gerri R Trooskin, Noel Coleman, Yuankun Zhu, Stephanie Stefankiewicz, Zied Abdullaev, Asif T Chinwalla, Mariarita Santi, Ammar S Naqvi, Jennifer L Mason, Carl J Koschmann, Xiaoyan Huang, Sharon J Diskin, Kenneth Aldape, Bailey K Farrow, Weiping Ma, Bo Zhang, Brian M Ennis, Sarah Tasian, Saksham Phul, Matthew R Lueder, Chuwei Zhong, Joseph M Dybas, Pei Wang, Deanne Taylor, Jo Lynne Rokita

**Affiliations:** Center for Data-Driven Discovery in Biomedicine, Children’s Hospital of Philadelphia, Philadelphia, PA 19104, USA; Division of Neurosurgery, Children’s Hospital of Philadelphia, Philadelphia, PA 19104, USA; Department of Biomedical and Health Informatics, Children’s Hospital of Philadelphia, Philadelphia, PA 19104, USA; Center for Data-Driven Discovery in Biomedicine, Children’s Hospital of Philadelphia, Philadelphia, PA 19104, USA; Division of Neurosurgery, Children’s Hospital of Philadelphia, Philadelphia, PA 19104, USA; Center for Cancer and Immunology Research, Children's National Hospital, Washington, DC 20010, USA; Department of Biomedical and Health Informatics, Children’s Hospital of Philadelphia, Philadelphia, PA 19104, USA; Center for Data-Driven Discovery in Biomedicine, Children’s Hospital of Philadelphia, Philadelphia, PA 19104, USA; Division of Neurosurgery, Children’s Hospital of Philadelphia, Philadelphia, PA 19104, USA; Center for Data-Driven Discovery in Biomedicine, Children’s Hospital of Philadelphia, Philadelphia, PA 19104, USA; Division of Neurosurgery, Children’s Hospital of Philadelphia, Philadelphia, PA 19104, USA; Department of Biomedical and Health Informatics, Children’s Hospital of Philadelphia, Philadelphia, PA 19104, USA; Department of Biomedical and Health Informatics, Children’s Hospital of Philadelphia, Philadelphia, PA 19104, USA; Department of Biomedical and Health Informatics, Children’s Hospital of Philadelphia, Philadelphia, PA 19104, USA; Department of Biomedical and Health Informatics, Children’s Hospital of Philadelphia, Philadelphia, PA 19104, USA; Department of Biomedical and Health Informatics, Children’s Hospital of Philadelphia, Philadelphia, PA 19104, USA; Center for Data-Driven Discovery in Biomedicine, Children’s Hospital of Philadelphia, Philadelphia, PA 19104, USA; Division of Neurosurgery, Children’s Hospital of Philadelphia, Philadelphia, PA 19104, USA; Center for Data-Driven Discovery in Biomedicine, Children’s Hospital of Philadelphia, Philadelphia, PA 19104, USA; Division of Neurosurgery, Children’s Hospital of Philadelphia, Philadelphia, PA 19104, USA; Center for Cancer and Immunology Research, Children's National Hospital, Washington, DC 20010, USA; Department of Biomedical and Health Informatics, Children’s Hospital of Philadelphia, Philadelphia, PA 19104, USA; Center for Data-Driven Discovery in Biomedicine, Children’s Hospital of Philadelphia, Philadelphia, PA 19104, USA; Division of Neurosurgery, Children’s Hospital of Philadelphia, Philadelphia, PA 19104, USA; Center for Data-Driven Discovery in Biomedicine, Children’s Hospital of Philadelphia, Philadelphia, PA 19104, USA; Division of Neurosurgery, Children’s Hospital of Philadelphia, Philadelphia, PA 19104, USA; Center for Data-Driven Discovery in Biomedicine, Children’s Hospital of Philadelphia, Philadelphia, PA 19104, USA; Division of Neurosurgery, Children’s Hospital of Philadelphia, Philadelphia, PA 19104, USA; Center for Data-Driven Discovery in Biomedicine, Children’s Hospital of Philadelphia, Philadelphia, PA 19104, USA; Division of Neurosurgery, Children’s Hospital of Philadelphia, Philadelphia, PA 19104, USA; Center for Cancer and Immunology Research, Children's National Hospital, Washington, DC 20010, USA; Center for Cancer and Immunology Research, Children's National Hospital, Washington, DC 20010, USA; Department of Pediatrics, George Washington University School of Medicine and Health Sciences, Washington, DC 20052, USA; Center for Data-Driven Discovery in Biomedicine, Children’s Hospital of Philadelphia, Philadelphia, PA 19104, USA; Division of Neurosurgery, Children’s Hospital of Philadelphia, Philadelphia, PA 19104, USA; Center for Data-Driven Discovery in Biomedicine, Children’s Hospital of Philadelphia, Philadelphia, PA 19104, USA; Division of Neurosurgery, Children’s Hospital of Philadelphia, Philadelphia, PA 19104, USA; Division of Oncology, Children’s Hospital of Philadelphia, Philadelphia, PA 19104, USA; Department of Pediatrics, University of Pennsylvania, Philadelphia, PA 19104, USA; Department of Biomedical and Health Informatics, Children’s Hospital of Philadelphia, Philadelphia, PA 19104, USA; Center for Childhood Cancer Research, Children’s Hospital of Philadelphia, Philadelphia, PA 19104, USA; Center for Data-Driven Discovery in Biomedicine, Children’s Hospital of Philadelphia, Philadelphia, PA 19104, USA; Division of Neurosurgery, Children’s Hospital of Philadelphia, Philadelphia, PA 19104, USA; Center for Data-Driven Discovery in Biomedicine, Children’s Hospital of Philadelphia, Philadelphia, PA 19104, USA; Division of Neurosurgery, Children’s Hospital of Philadelphia, Philadelphia, PA 19104, USA; Center for Data-Driven Discovery in Biomedicine, Children’s Hospital of Philadelphia, Philadelphia, PA 19104, USA; Division of Neurosurgery, Children’s Hospital of Philadelphia, Philadelphia, PA 19104, USA; Center for Data-Driven Discovery in Biomedicine, Children’s Hospital of Philadelphia, Philadelphia, PA 19104, USA; Division of Neurosurgery, Children’s Hospital of Philadelphia, Philadelphia, PA 19104, USA; Center for Data-Driven Discovery in Biomedicine, Children’s Hospital of Philadelphia, Philadelphia, PA 19104, USA; Division of Neurosurgery, Children’s Hospital of Philadelphia, Philadelphia, PA 19104, USA; Laboratory of Pathology, National Cancer Institute, Bethesda, MD 20892, USA; Department of Biomedical and Health Informatics, Children’s Hospital of Philadelphia, Philadelphia, PA 19104, USA; Department of Pathology and Laboratory Medicine, Children’s Hospital of Philadelphia, Philadelphia, PA 19104, USA; Department of Pathology and Laboratory Medicine, University of Pennsylvania Perelman School of Medicine, Philadelphia, PA 19104, USA; Center for Data-Driven Discovery in Biomedicine, Children’s Hospital of Philadelphia, Philadelphia, PA 19104, USA; Division of Neurosurgery, Children’s Hospital of Philadelphia, Philadelphia, PA 19104, USA; Center for Data-Driven Discovery in Biomedicine, Children’s Hospital of Philadelphia, Philadelphia, PA 19104, USA; Division of Neurosurgery, Children’s Hospital of Philadelphia, Philadelphia, PA 19104, USA; Department of Pediatrics, University of Michigan Health, Ann Arbor, MI 48105, USA; Pediatric Hematology Oncology, Mott Children’s Hospital, Ann Arbor, MI 48109, USA; Center for Data-Driven Discovery in Biomedicine, Children’s Hospital of Philadelphia, Philadelphia, PA 19104, USA; Division of Neurosurgery, Children’s Hospital of Philadelphia, Philadelphia, PA 19104, USA; Division of Oncology, Children’s Hospital of Philadelphia, Philadelphia, PA 19104, USA; Department of Pediatrics, University of Pennsylvania, Philadelphia, PA 19104, USA; Center for Data-Driven Discovery in Biomedicine, Children’s Hospital of Philadelphia, Philadelphia, PA 19104, USA; Division of Neurosurgery, Children’s Hospital of Philadelphia, Philadelphia, PA 19104, USA; Department of Genetics and Genomic Sciences, Icahn School of Medicine at Mount Sinai, New York, NY 10029, USA; Tisch Cancer Institute, Icahn School of Medicine at Mount Sinai, New York, NY 10029, USA; Center for Data-Driven Discovery in Biomedicine, Children’s Hospital of Philadelphia, Philadelphia, PA 19104, USA; Division of Neurosurgery, Children’s Hospital of Philadelphia, Philadelphia, PA 19104, USA; Center for Data-Driven Discovery in Biomedicine, Children’s Hospital of Philadelphia, Philadelphia, PA 19104, USA; Division of Neurosurgery, Children’s Hospital of Philadelphia, Philadelphia, PA 19104, USA; Division of Oncology, Children’s Hospital of Philadelphia, Philadelphia, PA 19104, USA; Department of Pediatrics, University of Pennsylvania, Philadelphia, PA 19104, USA; Center for Data-Driven Discovery in Biomedicine, Children’s Hospital of Philadelphia, Philadelphia, PA 19104, USA; Division of Neurosurgery, Children’s Hospital of Philadelphia, Philadelphia, PA 19104, USA; Center for Data-Driven Discovery in Biomedicine, Children’s Hospital of Philadelphia, Philadelphia, PA 19104, USA; Division of Neurosurgery, Children’s Hospital of Philadelphia, Philadelphia, PA 19104, USA; Department of Pathology and Laboratory Medicine, Children’s Hospital of Philadelphia, Philadelphia, PA 19104, USA; Center for Data-Driven Discovery in Biomedicine, Children’s Hospital of Philadelphia, Philadelphia, PA 19104, USA; Division of Neurosurgery, Children’s Hospital of Philadelphia, Philadelphia, PA 19104, USA; Center for Data-Driven Discovery in Biomedicine, Children’s Hospital of Philadelphia, Philadelphia, PA 19104, USA; Division of Neurosurgery, Children’s Hospital of Philadelphia, Philadelphia, PA 19104, USA; Tisch Cancer Institute, Icahn School of Medicine at Mount Sinai, New York, NY 10029, USA; Department of Biomedical and Health Informatics, Children’s Hospital of Philadelphia, Philadelphia, PA 19104, USA; Department of Pediatrics, University of Pennsylvania Perelman Medical School, Philadelphia, PA 19104, USA; Center for Data-Driven Discovery in Biomedicine, Children’s Hospital of Philadelphia, Philadelphia, PA 19104, USA; Division of Neurosurgery, Children’s Hospital of Philadelphia, Philadelphia, PA 19104, USA; Department of Biomedical and Health Informatics, Children’s Hospital of Philadelphia, Philadelphia, PA 19104, USA; Center for Cancer and Immunology Research, Children's National Hospital, Washington, DC 20010, USA; Department of Pediatrics, George Washington University School of Medicine and Health Sciences, Washington, DC 20052, USA

**Keywords:** pediatric cancer, open science, reproducibility, multiomics, Docker, OpenPedCan

## Abstract

**Background:**

In 2019, the Open Pediatric Brain Tumor Atlas (OpenPBTA) was created as a global, collaborative open-science initiative to genomically characterize 1,074 pediatric brain tumors and 22 patient-derived cell lines. Here, we present an extension of the OpenPBTA called the Open Pediatric Cancer (OpenPedCan) Project, a harmonized open-source multiomic dataset from 6,112 pediatric cancer patients with 7,096 tumor events across more than 100 histologies. Combined with RNA sequencing (RNA-seq) from the Genotype-Tissue Expression and The Cancer Genome Atlas projects, OpenPedCan contains nearly 48,000 total biospecimens (24,002 tumor and 23,893 normal specimens).

**Findings:**

We utilized Gabriella Miller Kids First workflows to harmonize whole-genome sequencing (WGS), whole exome sequencing (WXS), RNA-seq, and Targeted Sequencing datasets to include somatic SNVs, indels, copy number variants, structural variants, RNA expression, fusions, and splice variants. We integrated summarized Clinical Proteomic Tumor Analysis Consortium whole-cell proteomics and phospho-proteomics data and miRNA sequencing data, as well as developed a methylation array harmonization workflow to include m-values, beta-values, and copy number calls. OpenPedCan contains reproducible, dockerized workflows in GitHub, CAVATICA, and Amazon Web Services (AWS) to deliver harmonized and processed data from over 60 scalable modules, which can be leveraged both locally and on AWS. The processed data are released in a versioned manner and accessible through CAVATICA or AWS S3 download (from GitHub) and queryable through PedcBioPortal and the National Cancer Institute’s pediatric Molecular Targets Platform. Notably, we have expanded Pediatric Brain Tumor Atlas molecular subtyping to include methylation information to align with the World Health Organization 2021 Central Nervous System Tumor classifications, allowing us to create research-grade integrated diagnoses for these tumors.

**Conclusions:**

OpenPedCan data and its reproducible analysis module framework are openly available and can be utilized and/or adapted by researchers to accelerate discovery, validation, and clinical translation.

## Data Description

**Figure 1:**
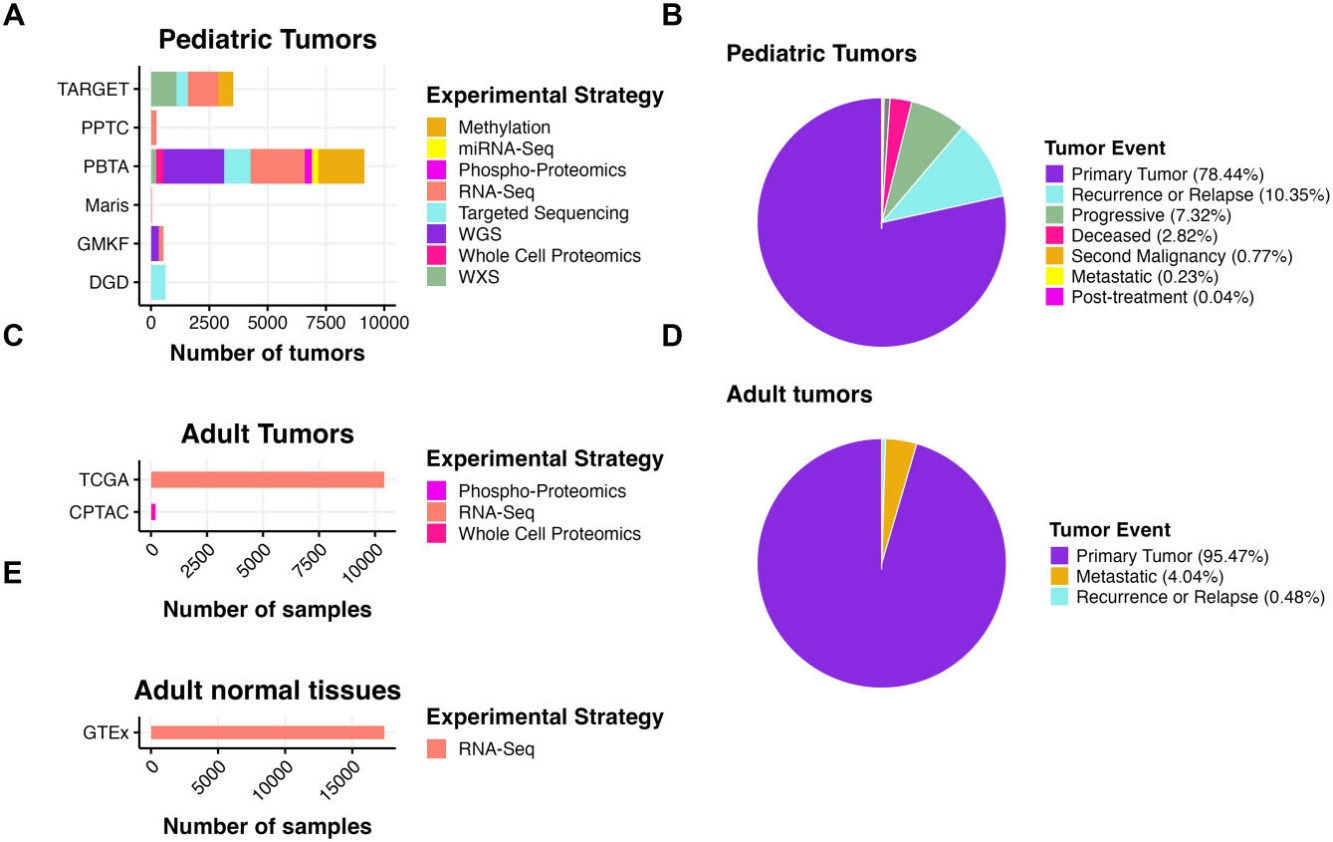
OpenPedCan data. (A) OpenPedCan contains multiomic data from 7 cohorts of pediatric tumors (A, B) with counts by tumor event, RNA-seq from adult tumors from The Cancer Genome Atlas (TCGA) Program (C, D), and RNA-seq from normal adult tissues from the Genotype-Tissue Expression (GTeX) project (E) with counts by specimen. Abbreviations: TARGET = Therapeutically Applicable Research to Generate Effective Treatments; PPTC = Pediatric Preclinical Testing Consortium; PBTA = Pediatric Brain Tumor Atlas; Maris = Neuroblastoma cell lines from the Maris Laboratory at CHOP; GMKF = Gabriella Miller Kids First; DGD = Division of Genomic Diagnostics at CHOP; CPTAC = Clinical Proteomic Tumor Analysis Consortium.

The Open Pediatric Cancer (OpenPedCan) project is an iterative open analysis effort in which we harmonize pediatric cancer data from multiple sources, perform downstream cancer analyses on these data, and provide them through Amazon S3, CAVATICA, PedcBioPortal, and v2.1 of the National Cancer Institute’s (NCI’s) Pediatric Molecular Targets Platform (MTP) [1]. We harmonized, aggregated, and analyzed data from multiple pediatric and adult data sources, building upon the work of the OpenPBTA (Fig. 1). All RNA sequencing (RNA-seq) and DNA sequencing (DNA-seq) data from OpenPBTA were updated from GENCODE v27 to GENCODE v39 as part of the OpenPedCan project. Further, all data within OpenPedCan are harmonized with GENCODE v39 annotations. Biospecimen-level metadata and clinical data are contained in Supplementary Table S1.

OpenPedCan currently include the following datasets, described more fully below:

- OpenPBTA
- TARGET
- Kids First Neuroblastoma (X01)
- Kids First PBTA (X01)
- Chordoma Foundation
- PPTC
- Maris
- MI-ONCOSEQ Study
- DGD
- GTEx
- TCGA
- CPTAC PBTA
- CPTAC GBM
- HOPE proteomics

### Open Pediatric Brain Tumor Atlas (OpenPBTA)

In September 2018, the Children’s Brain Tumor Network (CBTN) [2, 3] released the Pediatric Brain Tumor Atlas (PBTA), a genomic dataset (whole-genome sequencing, whole-exome sequencing, RNA sequencing, proteomic, and clinical data) for nearly 1,000 tumors, available from the Gabriella Miller Kids First Portal [4]. In September 2019, the Open Pediatric Brain Tumor Atlas (OpenPBTA) Project was launched. OpenPBTA was a global open science initiative to comprehensively define the molecular landscape of tumors of 943 patients from the CBTN and the PNOC003 DIPG clinical trial from the Pediatric Neuro-oncology Consortium [5] through real-time, collaborative analyses and collaborative manuscript writing on GitHub [6]. Additional PBTA data have been, and will be continually added to, OpenPedCan.

### Therapeutically Applicable Research to Generate Effective Treatments (TARGET)

The TARGET [7] Initiative is an NCI-funded collection of disease-specific projects that seeks to identify the genomic changes of pediatric cancers. The overall goal is to collect genomic data to accelerate the development of more effective therapies. OpenPedCan analyses include newly harmonized, open-access data associated with the 7 diseases present in the TARGET dataset: acute lymphoblastic leukemia (ALL), acute myeloid leukemia (AML), clear cell sarcoma of the kidney, neuroblastoma, osteosarcoma, rhabdoid tumor, and Wilm’s tumor.

### Gabriella Miller Kids First Neuroblastoma and PBTA

The Gabriella Miller Kids First Pediatric Research Program (Kids First) is a large-scale effort to accelerate research and gene discovery in pediatric cancers and structural birth defects. The program includes whole-genome sequencing (WGS) from patients with pediatric cancers and structural birth defects and their families. OpenPedCan analyses include neuroblastoma [8] and PBTA [9] data from the Kids First projects.

### Chordoma Foundation

The Chordoma Foundation [10] seeks to advance research and improve health care for patients diagnosed with chordoma and has shared patient and model sequencing data with the CBTN.

### Pediatric Preclinical Testing Consortium (PPTC)

The NCI’s former PPTC, now the Pediatric Preclinical in Vivo Testing (PIVOT) Program [11], molecularly and pharmacologically characterizes cell-derived and patient-derived xenograft (PDX) models. OpenPedCan includes re-harmonized RNA-Seq data for 244 models from the initial PPTC study [12, 13]. A subset of PPTC includes neuroblastoma models; the Maris cohort includes reharmonized RNA-seq data for 39 neuroblastoma cell lines [14], some of which have corresponding PDX models within the PPTC.

### MI-ONCOSEQ study

The MI-ONCOSEQ study [15] includes clinical sequencing data from the University of Michigan that were donated to CBTN and added to the PBTA cohort.

### Division of Genomic Diagnostics at Children’s Hospital of Philadelphia (DGD)

CHOP’s Division of Genomic Diagnostics has partnered with CCDI to add somatic panel sequencing data to OpenPedCan and the Molecular Targets Platform.

### The Genotype-Tissue Expression Project (GTEx)

The GTEx project [16] is an ongoing effort to build a comprehensive public data resource and tissue bank to study tissue-specific gene expression, regulation, and their relationship with genetic variants. Samples were collected from 54 nondiseased tissue sites across nearly 1,000 individuals, primarily for molecular assays, including WGS, WXS, and RNA-seq. OpenPedCan project includes 17,382 GTEx RNA-seq samples from GTEx v8 release, which span across 31 GTEx groups in the v12 release.

### The Cancer Genome Atlas Program (TCGA)

TCGA [17] is a landmark cancer genomics program that molecularly characterized over 20,000 primary cancer and matched normal samples spanning 33 cancer types. It is a joint effort between NCI and the National Human Genome Research Institute. OpenPedCan project includes open-access 10,414 RNA-seq for 716 normal and 9,698 TCGA tumor samples from 33 cancer types.

### Clinical Proteomic Tumor Analysis Consortium (CPTAC) PBTA proteomics study

The CPTAC pediatric pan-brain tumor study [18] contains 218 tumors profiled by proteogenomics and are included in OpenPedCan.

### CPTAC adult GBM proteomics study

This CPTAC adult GBM study [19] contains 99 tumors profiled by proteogenomics and are included in OpenPedCan.

### Project HOPE proteomics study

Project HOPE is an adolescent and young adult high-grade glioma study (in preparation for publication) that contains 90 tumors profiled by proteogenomics and are included in OpenPedCan.

OpenPedCan represents a substantial expansion since the OpenPBTA, both in cohort size and in data modality integration. By incorporating methylation, proteomics, splicing, and reference datasets, as well as enabling reproducible analyses across more than 48,000 biospecimens, OpenPedCan delivers a uniquely scalable and reusable resource for pediatric cancer research.

## Context

Creation of this dataset had multiple motivations. First, we sought to harmonize, summarize, and contextualize pediatric cancer genomics data among normal tissues (GTEx) and adult cancer tissues (TCGA) to enable the creation of the NCI’s MTP [1]. The inclusion of harmonized GTEx and adult TCGA data specifically allows for the identification of genes and/or transcripts expressed in a tumor-specific and/or pediatric tumor-specific manner. Next, we created this resource for broad community use to promote rapid reuse and accelerate the discovery of additional mechanisms contributing to the pathogenesis of pediatric cancers and/or to identify novel candidate therapeutic targets for pediatric cancer.

Similar to OpenPBTA, OpenPedCan operates on a pull request model to accept contributions. We set up continuous integration software via GitHub Actions to confirm the reproducibility of analyses within the project’s Docker container. We maintained a data release folder on Amazon S3, downloadable directly from S3 or our open-access CAVATICA project, with merged files for each analysis. As we produced new results, identified data issues, or added additional data, we created new data releases in a versioned manner. The project maintainers have included engineers and scientists from the Children’s Hospital of Philadelphia and Children’s National Hospital.

## Methods

**Figure 2:**
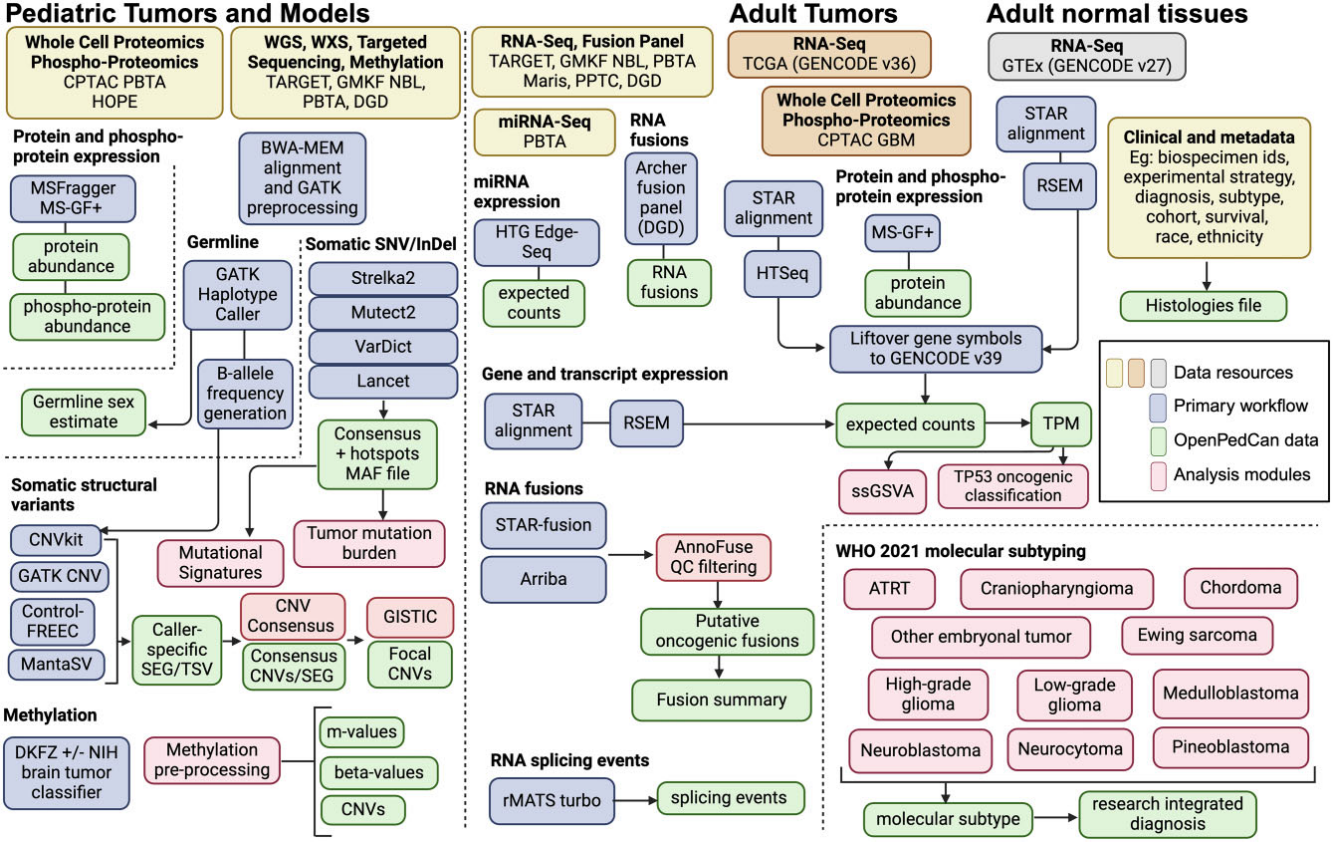
OpenPedCan analysis workflow. Depicted are the datasets (yellow, orange, and gray) contained within OpenPedCan. These datasets are made available in a harmonized manner through primary analysis workflows (blue) for DNA, RNA, and/or proteogenomics data. Files derived from the primary analysis workflows (green) are released within OpenPedCan. Additional analysis modules developed within OpenPedCan (red) also generate results files (green), which are released within OpenPedCan (Figure created in BioRender, https://BioRender.com/05gdk8k).

An overview of the OpenPedCan methods is depicted in Fig. 2. Briefly, most primary data harmonization analysis workflows were performed with Kids First pipelines written in Common Workflow Language (CWL) using CAVATICA (detailed below). Alignment and expression quantification for GTEx and TCGA RNA-seq were performed by the respective consortium. Custom Python, R, and/or bash scripts were then created in OpenPedCan using the primary harmonized output files.

### Sample details

A list of all biospecimens and associated metadata can be found in Supplementary Table S1.

#### Nucleic acids extraction and library preparation (PBTA X01 and miRNA-seq)

For detailed methods about the OpenPBTA cohort, please refer to [6]. For the PBTA X01 cohort, libraries were prepped using the Illumina TruSeq Strand-Specific Protocol to pull out poly-adenylated transcripts.

#### cDNA library construction

Total RNA was quantified using the Quant-iT RiboGreen RNA Assay Kit and normalized to 5 ng/μL. Following plating, 2 μL of ERCC controls (using a 1:1,000 dilution) was spiked into each sample. An aliquot of 325 ng for each sample was transferred into library preparation. The resultant 400-bp cDNA went through dual-indexed library preparation: “A” base addition, adapter ligation using P7 adapters, and PCR enrichment using P5 adapters. After enrichment, the libraries were quantified using Quant-iT PicoGreen (1:200 dilution). Samples were normalized to 5 ng/μL. The sample set was pooled and quantified using the KAPA Library Quantification Kit for Illumina Sequencing Platforms.

#### miRNA extraction and library preparation

Total RNA for CBTN samples was extracted as described in OpenPBTA [6] and prepared according to the HTG EdgeSeq protocol for the extracted RNA miRNA whole transcriptome assay (WTA). Then, 15 ng RNA was mixed in 25 μL of lysis buffer, which was then loaded onto a 96-well plate. Human Fetal Brain Total RNA (Takara Bio USA, #636,526) and Human Brain Total RNA (Ambion) were used as controls. The plate was loaded into the HTG EdgeSeq processor along with the miRNA WTA assay reagent pack. Samples were processed for 18–20 hours, then were barcoded and amplified using a unique forward and reverse primer combination. PCR settings used for barcoding and amplification were 95°C for 4 minutes, 16 cycles of (95°C for 15 seconds, 56°C for 45 seconds, 68°C for 45 seconds), and 68°C for 10 minutes. Barcoded and amplified samples were cleaned using AMPure magnetic beads (Ampure XP, cat. A63881). Libraries were quantified using the KAPA Biosystem assay qPCR kit (Kapa Biosystems, cat. KK4824), and CT values were used to determine the pM concentration of each library.

#### Data generation

**PBTA X01 Illumina Sequencing** Pooled libraries were normalized to 2 nM and denatured using 0.1 N NaOH prior to sequencing. Flowcell cluster amplification and sequencing were performed according to the manufacturer’s protocols using the NovaSeq 6000. Each run was a 151-bp paired-end with an 8-base index barcode read. Data were analyzed using the Broad Picard Pipeline, which includes de-multiplexing and data aggregation.

**PBTA miRNA Sequencing** Libraries were pooled, denatured, and loaded onto sequencing cartridge. Libraries were sequenced using an Illumina Nextseq 500 per manufacturer guidelines. FASTQ files were generated from raw sequencing data using Illumina BaseSpace and analyzed with the HTG EdgeSeq Parser software v5.4.0.7543 to generate an Excel file containing quantification of 2,083 miRNAs per sample. Any sample that did not pass the quality control set by the HTG REVEAL software version 2.0.1 was excluded from the analysis.

### Primary workflows through Kids First

#### DNA WGS alignment and SNP calling

Please refer to the OpenPBTA manuscript for details on DNA WGS alignment, prediction of participants’ genetic sex, and single-nucleotide polymorphism (SNP) calling for B-allele frequency (BAF) generation. [6].

#### Somatic mutation and indel calling

For matched tumor/normal samples, we used the same mutation calling methods as described in the OpenPBTA manuscript [6]. For tumor-only samples, we ran Mutect2 from GATK v4.2.2.0 using the following workflow [20].

#### VCF annotation and MAF creation

Somatic variants were annotated by the Ensembl Variant Effect Predictor (VEP v105) [21]. From tumor-only variant calls, we removed variants with alt_depth == 0 or t_depth < 4.

#### Consensus SNV calling (tumor/normal only)

We adopted the consensus single-nucleotide variant (SNV) calling method described in the OpenPBTA manuscript with adjustment [6]. For SNV calling, we combined 4 consensus SNV calling algorithms: Strelka2 [22], Mutect2 [23], Lancet [24], and VarDict [25].

Strelka2 outputs multinucleotide polymorphisms (MNPs) as consecutive single-nucleotide polymorphisms. In order to preserve MNPs, we gather MNP calls from the other caller inputs and search for evidence supporting these consecutive SNP calls as MNP candidates. Once found, the Strelka2 SNP calls supporting a MNP are converted to a single MNP call. This is done to preserve the predicted gene model as accurately as possible in our consensus calls. Consensus SNVs from all 4 callers were collected, and by default, calls that were detected in at least 2calling algorithms or marked with “HotSpotAllele” were retained.

For all SNVs, potential non-hotspot germline variants were removed if they had a normal depth ≤7 and gnomAD allele frequency >0.001. Final results were saved in MAF format.

#### Somatic copy number variant calling

We called copy number variants (CNVs) for tumor/normal samples using Control-FREEC [26, 27] and CNVkit [28], as described in the OpenPBTA manuscript [6]. We used GATK [29] to call CNVs for matched tumor/normal WGS samples when there were at least 30 male and 30 female normals from the same sequencing platform available for a panel of normal creation. For tumor-only samples, we used Control-FREEC with the following modifications. Instead of the b-allele frequency germline input file, we used the dbSNP_v153_ucsc-compatible.converted.vt.decomp.norm.common_snps.vcf.gz dbSNP common snps file [30], and to avoid hard-to-call regions, we utilized the hg38_canonical_150.mappability mappability file [31]. Both are also linked in the public Kids First references CAVATICA project [32]. The Control-FREEC tumor-only workflow can be found in the Kids First GitHub repository [33].

#### Somatic structural variant calling (WGS samples only)

We called structural variants (SVs) using Manta [34], restricting analysis to the same regions utilized by Strelka2. We annotated SVs using AnnotSV [35].

#### Gene expression

The tumor-normal-differential-expression module performs differential expression analyses for all sets of Disease (cancer_group) and Dataset (cohort) across all genes found in the gene-expression-rsem-tpm-collapsed.rds table. The purpose of this analysis is to highlight the correlation and understand the variability in gene expression in different cancer conditions across different histological tissues. For OpenPedCan v12 data release, this module performs expression analysis over 102 cancer groups across 52 histological tissues for all 54,346 genes found in the dataset. This analysis was performed on the Children’s Hospital of Philadelphia HPC and was configured to use 96 G of RAM per CPU, with 1 task (1 iteration of expression analysis for each set of tissue and cancer group) per CPU (total 102 × 52 = 5,304 CPUs) using the R/DESeq2 [36] package. Please refer to script run-tumor-normal-differential-expression.sh in the module for additional details on Slurm processing configuration. The same analysis can also be performed on CAVATICA but requires further optimization. The module describes the steps for CAVATICA setup and scripts to publish an application on the portal. The required data files are also available publicly on CAVATICA [37]. Refer to the module for detailed description and scripts.

### Abundance estimation

Among the data sources used for OpenPedCan, GTEx and TCGA used GENCODE v27 and v36, respectively. Therefore, the gene symbols had to be harmonized to GENCODE v39 for compatibility with the rest of the dataset. The liftover process was done via a custom script [38]. The script first constructs an object detailing the gene symbol changes from the HGNC symbol database [39]. Using the symbol-change object, the script updates any columns containing gene symbols. This liftover process was used on GTEx RNA-seq, TCGA RNA-seq, DGD fusions, and DNA hotspot files.

Additionally, the gene expression matrices had some instances where multiple Ensembl gene identifiers mapped to the same gene symbol. This was dealt with by filtering the expression matrix to only genes with [FPKM/TPM] >0 and then selecting the instance of the gene symbol with the maximum mean [FPKM/TPM/Expected_count] value across samples. This enabled many downstream modules that require RNA-seq data have gene symbols as unique gene identifiers. Refer to collapse-rnaseq module for scripts and details.

### Gene fusion detection from RNA-seq

Gene fusions were called using Arriba [40] and STAR-Fusion [41], as previously reported in OpenPBTA [6]. We updated the annoFuseData R package [42] to liftover gene symbols to be concordant with VEP v105. Fusions are now filtered with annoFuse [43, 44] upstream and released in fusion-annoFuse.tsv.gz.

### Gene fusion detection from fusion panels (DGD only)

Clinical RNA fusion calls from the CHOP DGD fusion panel [45] are included in the data release in the fusion-dgd.tsv.gz file.

#### Splicing quantification

To detect alternative splicing events, we utilized rMATS turbo (v. 4.1.0) with Ensembl/GENCODE v39 GFF annotations using the Kids First RNA-seq workflow [46]. We used –variable-read-length and -t paired options and applied an additional filter to include only splicing events with total junction read counts greater than 10. The OpenPedCan data release file splice-events-rmats.tsv.gz contains predicted single exon (SE), alternative 5′ splice site (A5SS), alternative 3′ splice site (A3SS), and retained intron (RI) events. These are made available for the community, but were not yet used in OpenPedCan analysis modules.

### Proteomics data integration

#### CPTAC PBTA, CPTAC GBM, and HOPE proteogenomics

The following methods are the general proteomics approaches used for the CPTAC PBTA [18], CPTAC GBM [19], and HOPE (prepublication, correspondence with Dr. Pei Wang) studies. For specific descriptions of sample preparation, mass spectrometry instrumentation and approaches, and data generation, processing, or analysis, please refer to the relevant publications.

### TMT-11 labeling and phosphopeptide enrichment

Proteome and phosphoproteome analyses of brain cancer samples in the CPTAC PBTA (pediatric), CPTAC GBM (adult), and HOPE (adolescent and young adult, AYA) cohort studies were structured as TMT11-plex experiments. Tumor samples were digested with LysC and trypsin. Digested peptides were labeled with TMT11-plex reagent and prepared for phosphopeptide enrichment. For each dataset, a common reference sample was compiled from representative samples within the cohort. Phosphopeptides were enriched using immobilized metal affinity chromatography (IMAC) with Fe3+-NTA-agarose bead kits.

### Liquid chromatography with tandem mass spectrometry analysis

To reduce sample complexity, peptide samples were separated by high pH reversed-phase HPLC fractionation. For CPTAC PBTA, a total of 96 fractions were consolidated into 12 final fractions for liquid chromatography with tandem mass spectrometry (LC-MS/MS) analysis. For CPTAC GBM and HOPE cohorts, a total of 96 fractions were consolidated into 24 fractions. For CPTAC PBTA, global proteome mass spectrometry analyses were performed on an Orbitrap Fusion Tribrid Mass Spectrometer, and phosphoproteome analyses were performed on an Orbitrap Fusion Lumos Tribrid Mass Spectrometer. For CPTAC GBM and HOPE studies, mass spectrometry analysis was performed using an Orbitrap Fusion Lumos Mass Spectrometer.

### Protein identification

The CPTAC PBTA spectra data were analyzed with MSFragger version 20,190,628 [47] searching against a CPTAC harmonized RefSeq-based sequence database containing 41,457 proteins mapped to the human reference genome (GRCh38/hg38) obtained via the UCSC Table Browser on 29 June 2018, with the addition of 13 proteins encoded in the human mitochondrial genome, 264 common laboratory contaminant proteins, and an equal number of decoy sequences. The CPTAC GBM and HOPE spectra data were analyzed with MS-GF+ v9881 [48–50] searching against the RefSeq human protein sequence database downloaded on 29 June 2018 (hg38; 41,734 proteins), combined with 264 contaminants, and a decoy database composed of the forward and reversed protein sequences.

### Protein quantification and data analysis

Relative protein (gene) abundance was calculated as the ratio of sample abundance to reference abundance using the summed reporter ion intensities from peptides mapped to the respective gene. For phosphoproteomic datasets, data were not summarized by protein but left at the phosphopeptide level. Global normalization was performed on the gene-level abundance matrix (log2 ratio) for global proteomic and on the site-level abundance matrix (log2 ratio) for phosphoproteomic data. The median, log2 relative protein or peptide abundance for each sample was calculated and used to normalize each sample to achieve a common median of 0. To identify TMT outliers, inter-TMT *t*-tests were performed for each individual protein or phosphopeptide. Batch effects were checked using the log2 relative protein or phosphopeptide abundance and corrected using the Combat algorithm [51]. Imputation was performed after batch effect correction for proteins or phosphopeptides with a missing rate <50%. For the phosphopeptide datasets, 440 markers associated with cold-regulated ischemia genes were filtered and removed.

### Creation of OpenPedCan Analysis modules

A list of all modules, repository links, one line description, and input and output files can be found in Supplementary Table S2.

### Methylation analysis

#### Methylation array preprocessing

We preprocessed raw Illumina 450 K and EPIC 850 K Infinium Human Methylation Bead Array intensities using the array preprocessing methods implemented in the minfi Bioconductor package [52]. We utilized either preprocessFunnorm when an array dataset had both tumor and normal samples or multiple OpenPedCan-defined cancer_groups and preprocessQuantile when an array dataset had only tumor samples from a single OpenPedCan-defined cancer_group to estimate usable methylation measurements (beta-values and m-values) and copy number (cn-values). Some Illumina Infinium array probes targeting CpG loci contain SNPs near or within the probe [53], which could affect DNA methylation measurements [54]. As the minfi preprocessing workflow recommends, we dropped probes containing common SNPs in dbSNP (minor allele frequency >1%) at the CpG interrogation or the single-nucleotide extensions.

Details of methylation array preprocessing are available in the OpenPedCan methylation-preprocessing module.

### Methylation classification of brain tumor molecular subtypes

The Clinical Methylation Unit Laboratory of Pathology at the National Cancer Institute Center for Cancer Research ran the DKFZ brain classifier version 12.6, a comprehensive DNA methylation-based classification of central nervous system (CNS) tumors across all entities and age groups [55] and/or the NIH Bethesda Brain tumor classifier v2.0 (NIH_v2) and the combo reporter pipeline v2.0 on Docker container trust1/bethesda:latest. Unprocessed IDAT-files from the CBTN Infinium Human Methylation EPIC (850k) BeadChip arrays were used as input, and the following information was compiled into the histologies.tsv file: dkfz_v12_methylation_subclass (predicted methylation subtype), dkfz_v12_methylation_subclass_score (classification score), dkfz_v12_methylation_mgmt_status (*MGMT* methylation status), dkfz_v12_methylation_mgmt_estimated (estimated *MGMT* methylation fraction), NIH_v2_methylation_Superfamily, NIH_v2_methylation_Superfamily_mean_score, NIH_v2_methylation_Superfamily_Consistency_score, NIH_v2_methylation_Class, NIH_v2_methylation_Class_mean_score, NIH_v2_methylation_Class_consistency_score, NIH_v2_methy-lation_Superfamily_match, and NIH_v2_methylation_Class_match.

#### Gene set variation analysis (gene-set-enrichment-analysis analysis module)

We performed gene set variation analysis (GSVA) for the Hallmark gene sets from MSigDB [56] on log2-transformed, gene-collapsed RSEM TPM expression values from RNA-seq using the GSVA package from Bioconductor [57]. GSVA was performed separately by RNA library type to avoid batch effects.

#### Fusion prioritization (fusion_filtering analysis module)

The fusion_filtering module filters artifacts and annotates fusion calls, with prioritization for oncogenic fusions, for the fusion calls from STAR-Fusion and Arriba. After artifact filtering, fusions were prioritized and annotated as “putative oncogenic fusions” when at least 1 gene was a known kinase, oncogene, tumor suppressor, curated transcription factor, on the COSMIC Cancer Gene Census List, or observed in TCGA. Fusions were retained in this module if they were called by both callers, recurrent or specific to a cancer group, or annotated as a putative oncogenic fusion. Please refer to the module linked above for more detailed documentation and scripts.

#### Consensus CNV calling (WGS samples only) (copy_number_consensus_call* analysis modules)

We adopted the consensus CNV calling described in the OpenPBTA manuscript [6] with minor adjustments. For each caller and sample with WGS performed, we called CNVs based on consensus among Control-FREEC [26, 27], CNVkit [28], and GATK [29]. Sample and consensus caller files with more than 2,500 CNVs were removed to de-noise and increase data quality, based on cutoffs used in GISTIC [58]. For each sample, we included the following regions in the final consensus set: (i) regions with reciprocal overlap of 50% or more between at least 2 of the callers and (ii) smaller CNV regions in which more than 90% of regions were covered by another caller. For GATK, if a panel of normal was not able to be created (required 30 male and 30 female with the same sequencing platform), consensus was run for that tumor using Control-FREEC, CNVkit, and MantaSV. We defined copy number as NA for any regions that had a neutral call for the samples included in the consensus file. We merged CNV regions within 10,000 bp of each other with the same direction of gain or loss into single region.

Any CNVs that overlapped 50% or more with immunoglobulin, telomeric, centromeric, or segment duplicated regions or that were shorter than 3,000 bp were filtered out. The CNVKit calls for WXS samples were appended to the consensus CNV file.

#### Focal copy number calling (focal-cn-file-preparation analysis module)

Please refer to the OpenPBTA manuscript for details on assignment of copy number status values to CNV segments, cytobands, and genes [6]. We applied criteria to resolve instances of multiple conflicting status calls for the same gene and sample, which are described in detail in the focal-cn-file-preparation module. Briefly, we prioritized (i) nonneutral status calls, (ii) calls made from dominant segments with respect to gene overlap, and (iii) amplification and deep deletion status calls over gain and loss calls, respectively, when selecting a dominant status call per gene and sample. These methods resolved >99% of duplicated gene-level status calls.

#### Mutational signatures (mutational-signatures analysis module)

We obtained mutational signature weights (i.e., exposures) from consensus SNVs using the deconstructSigs R package [59]. We estimated weights for single- and double-base substitution (SBS and DBS, respectively) signatures from the Catalogue of Somatic Mutations in Cancer (COSMIC) database versions 2 and 3.3, as well as SBS signatures from Alexandrov et al. [60]. The following COSMIC SBS signatures were excluded from weight estimation in all tumors: (i) sequencing artifact signatures, (ii) signatures associated with environmental exposure, and (iii) signatures with an unknown etiology. Additionally, we excluded therapy-associated signatures from mutational signature weight estimation in tumors collected prior to treatment (i.e., “Initial CNS Tumor” or “Primary Tumor”).

#### Tumor mutation burden (tmb-calculation analysis module)

Recent clinical studies have associated high tumor mutation burden (TMB) with improved patient response rates and survival benefit from immune checkpoint inhibitors [61].

The TMB tmb-calculation module was adapted from the snv-callers module of the OpenPBTA project [6]. Here, we use mutations in the snv-consensus-plus-hotspots.maf.tsv.gz file, which is generated using Kids First DRC Consensus Calling Workflow [62] and is included in the OpenPedCan data download. The consensus MAF contains SNVs or MNVs called in at least 2 of the 4 callers (Mutect2, Strelka2, Lancet, and Vardict) plus hotspot mutations if called in 1 of the 4 callers. We calculated TMB for tumor samples sequenced with either WGS or WXS. Briefly, we split the SNV consensus MAF into SNVs and MNVs. We split the MNV subset into SNV calls, merged those back with the SNVs subset, and then removed sample-specific redundant calls. The resulting merged and nonredundant SNV consensus calls were used as input for the TMB calculation. We tallied only nonsynonymous variants with classifications of high/moderate consequence (“Missense_Mutation,” “Frame_Shift_Del,” “In_Frame_Ins,” “Frame_Shift_Ins,” “Splice_Site,” “Nonsense_Mutation,” “In_Frame_Del,” “Nonstop_Mutation,” and “Translation_Start_Site”) for the numerator. All BED files are provided in the data release.

### All-mutation TMB

For WGS samples, we calculated the size of the genome covered as the intersection of Strelka2 and Mutect2’s effectively surveyed areas, regions common to all variant callers, and used this as the denominator. WGS_all_mutations_TMB = (total # mutations in consensus MAF)/intersection_strelka_mutect_vardict_genome_size. For WXS samples, we used the size of the WXS bed region file as the denominator. WXS_all_mutations_TMB = (total # mutations in consensus MAF))/wxs_genome_size.

### Coding-only TMB

We generated coding only TMB from the consensus MAF as well. We calculated the intersection for Strelka2 and Mutect2 surveyed regions using the coding sequence ranges in the GENCODE v39 gtf supplied in the OpenPedCan data download. We removed SNVs outside of these coding sequences prior to implementing the TMB calculation below: WGS_coding_only_TMB = (total # coding mutations in consensus MAF)/intersection_wgs_strelka_mutect_vardict_CDS_genome_size. For WXS samples, we intersected each WXS bed region file with the GENCODE v39 coding sequence, sum only variants within this region for the numerator, and calculate the size of this region as the denominator. WXS_coding_only_TMB = (total # coding mutations in consensus MAF)/intersection_wxs_CDS_genome_size.

Finally, we include an option (nonsynfilter_focr) to use specific nonsynonymous mutation variant classifications recommended from the TMB Harmonization Project [63].

#### Molecular subtyping

Here, we build upon the molecular subtyping performed in OpenPBTA [6] to align with WHO 2021 subtypes [64]. Molecular subtypes were generated per tumor event and are listed for each biospecimen in Supplementary Table S1, with the number of tumors grouped by broad histology and molecular subtype in Supplementary Table S3.

### High-grade gliomas

High-grade gliomas (HGGs) were categorized based on a combination of clinical information, molecular features, and DNA methylation data. H3 K28-altered diffuse midline gliomas (DMGs) were classified based on the presence of a p.K28M or p.K28I mutation in *H3F3A, HIST1H3B, HIST1H3C*, or *HIST2H3C*, or a high-confidence DKFZ methylation score (≥0.8) in the appropriate subclass. Oligodendroglioma, IDH-mutant tumors were classified based on high-confidence “O_IDH” methylation classifications, and oligosarcoma, IDH-mutant tumors were defined as those with high-confidence “OLIGOSARC_IDH” methylation classifications. Pleomorphic xanthoastrocytomas (PXAs) were classified using the following criteria: (i) methylation subtype is high-confidence “PXA” or pathology_free_text_diagnosis contains “pleomorphic xanthoastrocytoma” or “pxa,” and (ii) tumor contains a *BRAF* V600E mutation and a *CDKN2A* or *CDKN2B* homozygous deletion. Methylation classifications were used in classifying the following subtypes:

1. DHG, H3 G35 (“DHG_G34” and “GBM_G34” classifications)
2. HGG, IDH (“A_IDH_HG” and “GBM_IDH” classifications)
3. HGG, H3 wild-type (methylation classification contains “GBM_MES,” “GBM_RTK,” “HGG_,” “HGAP,” “AAP,” or “ped_”)

A new high-grade glioma entity, called infant-type hemispheric gliomas (IHGs), characterized by distinct gene fusions enriched in receptor tyrosine kinase (RTK) genes, including *ALK, NTRK1/2/3, ROS1*, or *MET*, was identified in 2021 [65]. To identify IHG tumors, first, tumors that were classified as “IHG” by the DKFZ methylation classifier or diagnosed as “infant type hemispheric glioma” from pathology_free_text_diagnosis were selected [55]. Then, the corresponding tumor RNA-seq data were utilized to seek the evidence for RTK gene fusion. Based on the specific RTK gene fusion present in the samples, IHGs were further classified as “IHG, ALK-altered,” “IHG, NTRK-altered,” “IHG, ROS1-altered,” or “IHG, MET-altered.” If no fusion was observed, the samples were identified as “IHG, To be classified.”

### Atypical teratoid rhabdoid tumors

Atypical teratoid rhabdoid tumors (ATRTs) were categorized into 3 subtypes: “ATRT, MYC,” “ATRT, SHH,” and “ATRT, TYR” [66]. In OpenPedCan, the molecular subtyping of ATRT was based solely on the DNA methylation data. Briefly, ATRT samples with a high-confidence DKFZ methylation subclass score (≥0.8) were selected, and subtypes were assigned based on the DKFZ methylation subclass [55]. Samples with low-confidence DKFZ methylation subclass scores (<0.8) were identified as “ATRT, To be classified.”

### Neuroblastoma tumors

Neuroblastoma (NBL) tumors with a pathology diagnosis of neuroblastoma, ganglioneuroblastoma, or ganglioneuroma were subtyped based on their MYCN copy number status as either “NBL, MYCN amplified” or “NBL, MYCN non-amplified.” If pathology_free_text_diagnosis was “NBL, MYCN non-amplified” and the genetic data suggested MYCN amplification, the samples were subtyped as “NBL, MYCN amplified.” On the other hand, if pathology_free_text_diagnosis was “NBL, MYCN amplified” and the genetic data suggested MYCN nonamplification, the RNA-seq gene expression level of *MYCN* was used as a prediction indicator. In those cases, samples with *MYCN* gene expression above or below the cutoff (TPM ≥140.83 based on visual inspection of MYCN CNV status) were subtyped as “NBL, MYCN amplified” and “NBL, MYCN non-amplified,” respectively. *MYCN* gene expression was also used to subtype samples without DNA sequencing data. If a sample did not fit none of these situations, it was denoted as “NBL, To be classified.”

### Craniopharyngiomas

In addition to molecular criteria established in OpenPBTA [6], craniopharyngiomas (CRANIO) are now subtyped using DNA methylation classifiers. Craniopharyngiomas with a high-confidence methylation subclass containing “CPH_PAP” were classified as papillary (CRANIO, PAP), and those with high-confidence methylation subclass containing “CPH_ADM” were classified as adamantinomatous (CRANIO, ADAM), respectively.

### Ependymomas

Ependymomas (EPNs) are subtyped using the following criteria:

1. Any spinal tumor with *MYCN* amplification or with a high-confidence “EPN, SP-MYCN” methylation classification was subtyped as EPN, spinal and MYCN-amplified (SP-MYCN).
2. EPN tumors containing 1 or more gene fusions of *YAP1::MAMLD1, YAP1::MAML2*, or *YAP1::FAM118B*, or else had a high-confidence “EPN, ST YAP1” methylation classification, were subtyped as EPN, ST YAP1.
3. EPN tumors containing 1 or more gene fusions of *ZFTA::RELA* or *ZFTA::MAML2*, or else had a high-confidence “EPN, ST ZFTA” methylation classification, were subtyped as EPN, ST ZFTA. This reflects an update to WHO classifications that now characterizes this subtype based on *ZFTA* fusions rather than *RELA* fusions.
4. EPN tumors with (i) chromosome 1q gain and *TKTL1* overexpression, or (ii) *EZHIP* overexpression, or (iii) posterior fossa anatomical location and a histone H3 K28 mutation in *H3F3A, HIST1H3B, HIST1H3C*, or *HIST2H3C*, or (iv) a high-confidence “EPN, PF A” methylation classification were subtyped as posterior fossa group A ependymomas (EPN, PF A).
5. Tumors with (i) chr 6p or 6q loss and *GPBP1* or *IFT46* overexpression, or (ii) a high-confidence “EPN, PF B” methylation classification were subtyped as posterior fossa group B ependymomas (EPN, PF B).
6. EPN tumors with a high-confidence “EPN, MPE” methylation classification were subtyped as myxopapillary ependymomas (EPN, MPE).
7. EPN tumors with a high-confidence “EPN, PF SE” methylation classification were subtyped as posterior fossa subependymomas (EPN, PF SE).
8. EPN tumors with a high-confidence “EPN, SP SE” methylation classification were subtyped as spinal subependymomas (EPN, SP SE).
9. EPN tumors with a high-confidence “EPN, SP” methylation classification were subtyped as spinal ependymomas (EPN, SP).
10. All other EPN tumors were classified as “EPN, To be classified.”

### Low-grade gliomas

In addition to subtyping methods described in OpenPBTA [6], high-confidence methylation classifications are now used in classifying the following low-grade glioma (LGG) subtypes:

1. LGG, other MAPK-altered (methylation subclass “PA_MID” or “PLNTY”)
2. LGG, FGFR-altered (methylation subclass “PA_INF_FGFR”)
3. LGG, IDH-altered (methylation subclass “A_IDH_LG”)
4. LGG, MYB/MYBL1 fusion (methylation subclass “AG_MYB” or “LGG_MYB”)
5. LGG, MAPK-altered (methylation subclass “LGG, MAPK”)
6. LGG, BRAF- and MAPK-altered (methylation subclass “LGG, BRAF/MAPK”)
7. SEGA, to be classified (methylation subclass “SEGA, To be classified”)

**Figure 3:**
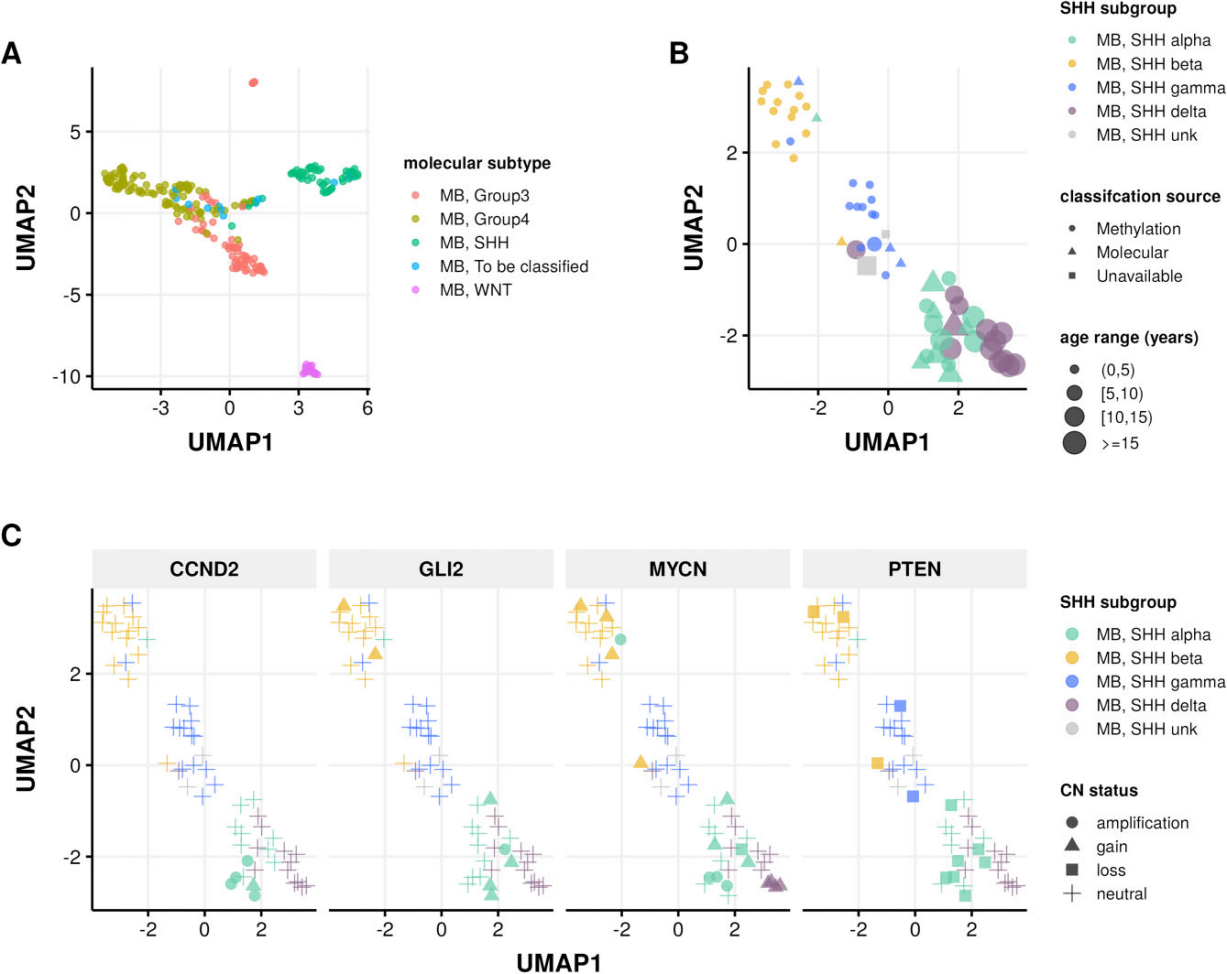
Medulloblastoma sample clustering. (A) UMAP projection of 271 MB tumors and (B), 63 SHH-activated MB tumors using methylation beta values of the 20,000 most variable probes from the Infinium MethylationEPIC array. (C) UMAP projection of MB, SHH-activated samples indicating copy number status of SHH subgroup known somatic driver genes CCND2, GLI2, MYCN, and PTEN.

**Medulloblastomas (MBs)** In addition to our previous work classifying MB tumors into the 4 major subtypes (WNT, SHH, group 3, and group 4) using the transcriptomic MedulloClassifier [67], we integrated high-confidence methylation classification, demographic, and molecular criteria to molecularly subtype SHH tumors into 1 of 4 subgroups (alpha, beta, gamma, or delta) (Fig. 3).

We implemented molecular subtyping as follows:

1. MB tumors with methylation classification that contains “MB_SHH” are subtyped as SHH-activated medulloblastoma (MB, SHH)
2. MB tumors with “MB_G34_I,” “MB_G34_II,” “MB_G34_III,” and “MB_G334_IV” methylation classifications are subtyped as medulloblastoma group 3 (MB, Group3)
3. MB tumors with “MB_G34_V,” “MB_G34_VI,” “MB_G34_VII,” and “MB_G334_VIII” methylation classifications are subtyped as medulloblastoma group 4 (MB, Group4)
4. MB tumors with “MB_WNT” methylation classification are subtyped as WNT-activated MB (MB, WNT)
5. MB tumors with “MB_MYO” methylation classification are subtyped as medulloblastomas with myogenic differentiation (MB, MYO)

We classified MB, SHH subtype tumors using the following criteria:

1. *MB, SHH alpha*: sample has a high-confidence “MB_SHH_3” methylation classification, or patient had an age at diagnosis ≥2 years and harbored 1 of the following molecular alterations in tumor or germline:
2. *MYCN, GLI2*, or *CCND2* amplification or sample TPM *z*-score ≥2 in tumor
3. A pathogenic or likely pathogenic germline variant in *ELP1* or *TP53*
4. A *TP53* hotspot mutation in tumor
5. Chromosome 9p gain or chromosome 17p loss in tumor
6. *MB, SHH beta*: sample has a high-confidence “MB_SHH_1” methylation classification, or patient had an age at diagnosis <5 years and harbored 1 of the following molecular alterations:
7. A *KMT2D* loss of function variant
8. *PTEN* copy number loss or deep deletion, or sample TPM z-score <−2.
9. Chromosome 2p or 2q gain
10. *MB, SHH gamma*: sample has a high-confidence “MB_SHH_2” methylation classification, or patient had an age at diagnosis <5 years and tumor harbored a chromosome 2p arm gain
11. *MB, SHH delta*: sample has a high-confidence “MB_SHH_4” methylation classification, or patient had an age at diagnosis ≥10 years and harbored 1 of the following molecular alterations in tumor:
12. A *DDX3X* or *SMO* loss-of-function mutation
13. A hotspot *TERT* or U1 snRNA gene mutation
14. Chromosome 14q arm loss.

### Pineoblastomas

Pineoblastomas (PBs) are classified as follows using high-confidence methylation classifications:

1. Pineoblastoma, MYC/FOXR2-activated (“PB_FOXR2” methylation classification)
2. Pineoblastoma, RB1-altered (“PB_RB1” methylation classification)
3. Pineoblastoma, group 1 (“PB_GRP1A” and “PB_GRP1B” methylation classifications)
4. Pineoblastoma, group 2 (“PB_GRP2” methylation classification)
5. All other pineoblastomas were classified as “PB, To be classified”

### Non-MB, non-ATRT embryonal tumors

Updates were made to non-MB, non-ATRT embryonal tumor subtyping as follows:

1. Embryonal tumors with multilayered rosettes and C19MC-altered (ETMR, C19MC-altered) were classified based on (i) high-confidence “ETMR_C19MC” methylation classification or (ii) *TTYH1* gene fusion and either chromosome 19 amplification or *LIN28A* overexpression.
2. ETMR, not otherwise specified (NOS) were classified based on *LIN28A* overexpression and no *TTYH1* gene fusion.

#### TP53 alteration annotation (tp53_nf1_score analysis module)

We classified TP53-altered HGG samples as either *TP53* lost or *TP53* activated and incorporated these annotations into the molecular subtype framework. To support this classification, we used a previously published RNA-based *TP53* inactivation signature originally developed using TCGA pan-cancer cohorts [68]. We applied this to OpenPedCan RNA-seq data, stratified by library preparation type. This classifier was used in combination with genomic variant data, including consensus SNVs, CNVs, and SVs, as well as curated reference databases cataloging somatic *TP53* hotspot mutations [69, 70] and known functional domains [71] to annotate lost or activated status. Briefly, samples were annotated as *TP53* activated if they harbored either of 2 known gain-of-function mutations: p.R273C or p.R248W [72]. Samples were assigned *TP53* lost status under any of the following conditions: (i) presence of a hotspot *TP53* mutation listed in the IARC or MSKCC databases, (ii) detection of 2 distinct TP53 alterations (e.g., SNV, CNV, or SV) consistent with biallelic inactivation, (iii) presence of a single somatic *TP53* variant or a pathogenic germline variant associated with Li-Fraumeni syndrome (LFS) [73], or (iv) presence of a germline *TP53* variant linked to LFS alongside a *TP53* inactivation classifier score >0.5 from matched RNA-seq data.

#### Clinical data harmonization

To remain consistent with the Kids First data model and our previous OpenPBTA study [6], all clinical metadata were harmonized using the same data model. TARGET and TCGA metadata fields (e.g., sample_type, composition, tumor_descriptor, etc.) were harmonized to those of Kids First. Additional histology-related fields were created through OpenPedCan, following molecular subtyping: integrated_diagnosis, harmonized_diagnosis, and cancer_group. These fields were expanded from our previous study, to utilize the WHO 2021 CNS tumor classifications [64]. Any samples with molecular subtypes that did not match the initial pathology_diagnosis were reviewed with a board-certified molecular pathologist and updated accordingly.

#### EFO, MONDO, and NCIT mapping

We created a script to search ontology mappings by cancer_group. The efo_code represents the Experimental Factor Ontology (EFO) description available in the European Bioinformatics Institute database, the mondo_code represents the Mondo Disease Ontology (MONDO) from an independent resource that aims to harmonize disease definitions, and the ncit_code represents the NCI Thesaurus (NCIt) reference terminology. Codes were automatically pulled based on text matching, manually reviewed, and can be found in Supplementary Table S1.

#### Selection of independent samples (independent-samples analysis module)

For analyses that require all input biospecimens to be independent, we use the OpenPedCan-analysis independent-samples module to select only 1 biospecimen from each input participant. For each input participant of an analysis, the independent biospecimen is selected based on the analysis-specific filters and preferences for the biospecimen metadata, such as experimental strategy, cancer group, and tumor descriptor.

## Data Validation and Quality Control

All RNA-seq and WGS samples passed minimum quality thresholds, including ≥20 million total reads and ≥50% alignment for RNA-seq, and ≥20× mean coverage for DNA sequencing Supplementary Table S4. Sample identity was confirmed using NGSCheckMate [74] and Somalier relate [75] to detect and exclude mismatched or contaminated samples.

We expanded upon the molecular subtyping modules from OpenPBTA to recover hallmark genomic and transcriptomic features known in pediatric tumors. These include *KIAA1549::BRAF* fusions in low-grade gliomas, H3 K28M/I mutations in diffuse midline gliomas, H3 G35R/V mutations in diffuse hemispheric gliomas, somatic *TP53* mutations in high-grade gliomas, and MYCN amplification in neuroblastoma, for example.

All subtyping modules are version-controlled, containerized, and publicly available, and they have undergone internal code review and validation by independent analysts. Where molecular features conflicted with original pathology labels, cases were reviewed with board-certified molecular pathologists, and integrated diagnoses were updated accordingly. This collaborative re-review process led to improved sample annotation and is fully documented in the molecular-subtype-pathology module.

To assess concordance between data types, we compared RNA-based and methylation-based molecular subtypes in medulloblastoma. As shown in Table 1, we observed nearly 100% concordance, validating both experimental modalities and classifier accuracy. Notably, methylation classification identified 1 rare case (MB, MYO) not captured by the transcriptome-based MedulloClassifier.

**Table 1:** Medulloblastoma subtype concordance across experimental strategies. Comparison of medulloblastoma subtypes using methylation or RNA-seq classification.

Methylation subtype	Group3 (RNA-seq)	Group4 (RNA-seq)	SHH (RNA-seq)	WNT (RNA-seq)
MB_G34_II	8	0	0	0
MB_G34_III	17	0	0	0
MB_G34_IV	8	0	0	0
MB_G34_V	0	6	0	0
MB_G34_VI	0	4	0	0
MB_G34_VII	0	30	0	0
MB_G34_VIII	0	34	0	0
MB_MYO	1	0	0	0
MB_SHH_1	0	0	11	0
MB_SHH_2	0	0	4	0
MB_SHH_3	0	0	2	0
MB_SHH_4	0	0	7	0
MB_WNT	0	0	0	18

In addition to verifying known findings, OpenPedCan modules support pediatric cancer discovery and translation. The reproducibility of these results is further supported by their reuse across studies, >100 Zenodo downloads, GitHub forks, and independent analysis pipelines. Together, these validation measures—spanning sample quality control, molecular feature recovery, cross-platform concordance, and expert review—ensure that OpenPedCan is a robust, reproducible, and reusable resource for the pediatric cancer research community.

## Ethics and Consent Statement

This study did not generate new sequencing data. All previously published raw data were obtained through Database of Genotypes and Phenotypes (dbGAP) access requests with patients consented as “General Research Use (GRU)” or “Disease-Specific (Pediatric Cancer Research).” OpenPedCan integrates only summary-level outputs (e.g., gene expression matrices, mutation calls) that are designated for GRU by the data custodians. No protected health information, raw sequencing files, or individually identifiable clinical metadata are distributed as part of this project.

## Reuse Potential

OpenPedCan represents a valuable resource, not only by significantly extending OpenPBTA to include more than 5,000 additional patients and 6,000 tumors, but also by adding a number of new “omic” data types not previously included, such as methylation arrays, miRNA-seq, proteomics, and normal tissue RNA-seq. An overview of the OpenPedCan data availability is summarized in Table 2. OpenPedCan also serves as a community resource whose outputs and/or code can be leveraged directly to ask research questions or serve as an orthogonal validation dataset. By providing these data in a harmonized manner, we enable investigators to reduce the financial and time-related costs associated with their analyses, which would otherwise total years of project hours and over $50,000 in data analysis alone [76]. We encourage reuuse of the data, ideas and suggestions for improving the data or adding analyses, and/or direct code contributions through a pull-request.

**Table 2:** OpenPedCan data availability. OpenPedCan data are available on multiple platforms with varying access requirements.

Platform	Data type	Access type	Access requirement
PedcBioPortal	Individual and summary somatic data	Query	Gmail account
Molecular Targets Platform	Cancer group summary data	Query	Open access
GitHub	Merged summary files	Full access	AWS S3 download script
CAVATICA	Merged summary files	Full access	CAVATICA account
dbGAP—phs000218.v23.p8	Raw data	Full access	Access request via institution
dbGAP—phs001436.v1.p1	Raw data	Full access	Access request via institution
dbGAP—phs001437.v2.p1	Raw data	Full access	Access request via institution
dbGAP—phs002517.v4.p2	Raw data	Full access	Access request via institution
dbGAP—phs000424.v9.p2	Raw data	Full access	Access request via institution
dbGAP—phs000178.v11.p8	Raw data	Full access	Access request via institution
NCI GDC Data Portal	Individual expression data	Full access	Open access
GTEx portal	Summary expression data	Full access	Open access
Proteomic Data Commons	Raw data	Full access	Open access

## Availability of Source Code and Requirements

Project name: The Open Pediatric Cancer (OpenPedCan) Project

Project homepage: https://github.com/d3b-center/OpenPedCan-analysis [77]

Archived source code: https://doi.org/10.5281/zenodo.15750097 [78]

Operating system(s): Platform independent

Programming languages: R, Python, bash

Other requirements: CAVATICA is required to run all primary Kids First workflows out of the box. All downstream OpenPedCan workflows can be run using the Docker image at pgc-images.sbgenomics.com/d3b-bixu/openpedcanverse:latest. Most workflows run efficiently on local or cloud machines with 16–64 GB RAM. The most memory-intensive module runs on a 64 GB instance at <$2 per run.

License: CC0 (data)/BSD3-clause (code)

Primary analyses were performed using Gabriella Miller Kids First pipelines and are listed in the Methods section. Analysis modules were either initially developed within Alex’s Lemonade Stand Foundation’s publicly available OpenPBTA-analysis GitHub repository: https://github.com/AlexsLemonade/OpenPBTA-analysis [6]; they were modified and/or created anew within the OpenPedCan GitHub repository: https://github.com/d3b-center/OpenPedCan-analysis [78].

Software versions are documented in Supplementary Table S5.

## Additional Files

**Supplementary Table S1**. README, metadata, and clinical data for each patient and biospecimen in OpenPedCan.

**Supplementary Table S2**. Description of OpenPedCan analysis modules. Listed are the modules, short descriptions, links, input files, whether the resulting files are contained in data releases, and which files are consumed in other analyses.

**Supplementary Table S3**. Number of tumors and corresponding patients from which WHO 2021 molecular subtypes were generated through OpenPedCan analysis modules are listed in Sheet 1. Molecular subgroups (alpha, beta, gamma, or delta) for medulloblastoma SHH tumors are listed in Sheet 2.

**Supplementary Table S4**. Read count and coverage for DNA and RNA sequencing biospecimens.

**Supplementary Table S5**. Listed are the software versions for all packages and workflows used in this article.

giaf093_Supplemental_Files

giaf093_Authors_Response_To_Reviewer_Comments_Original_Submission

giaf093_Authors_Response_To_Reviewer_Comments_Revision_1

giaf093_GIGA-D-25-00086_Original_Submission

giaf093_GIGA-D-25-00086_Revision_1

giaf093_GIGA-D-25-00086_Revision_2

giaf093_Reviewer_1_Report_Original_Submission Stephen R Piccolo, Ph.D. -- 3/28/2025

giaf093_Reviewer_1_Report_Revision_1 Stephen R Piccolo, Ph.D. -- 7/2/2025

giaf093_Reviewer_2_Report_Original_Submission Jacek Majewski -- 4/7/2025

## Abbreviations

ATRT: atypical teratoid rhabdoid tumor; BAF: B-allele frequency; CBTN: Children’s Brain Tumor Network; CNS: central nervous system; COSMIC: Catalogue of Somatic Mutations in Cancer; CPTAC: Clinical Proteomic Tumor Analysis Consortium; CPTAC: Clinical Proteomic Tumor Analysis Consortium; DGD: Division of Genomic Diagnostics at CHOP; DMG: diffuse midline glioma; GMKF: Gabriella Miller Kids First; GSVA: gene set variation analysis; GTeX: Genotype-Tissue Expression; HGG: high-grade glioma; IHG: infant-type hemispheric glioma; LC-MS/MS: liquid chromatography with tandem mass spectrometry; LFS: Li-Fraumeni syndrome; LGG: low-grade glioma; MNP: multinucleotide polymorphism; MTP: Molecular Targets Platform; NBL: neuroblastoma; NCI: National Cancer Institute; OpenPBTA: Open Pediatric Brain Tumor Atlas; OpenPedCan: Open Pediatric Cancer; PBTA: Pediatric Brain Tumor Atlas; PDX: patient-derived xenograft; PPTC: Pediatric Preclinical Testing Consortium; PPTC: Pediatric Preclinical Testing Consortium; PXA: pleomorphic xanthoastrocytoma; SNP: single-nucleotide polymorphism; SNV: single-nucleotide variant; SV: structural variant; TARGET: Therapeutically Applicable Research to Generate Effective Treatments; TCGA: The Cancer Genome Atlas Program; TMB: tumor mutation burden; WGS: whole-genome sequencing; WTA: whole transcriptome assay.

## Data Availability

The datasets supporting this study are available as follows: The TARGET dataset is available in dbGAP under phs000218.v23.p8 [7], and processed somatic data are openly available at the National Cancer Institute (NCI) Genomic Data Commons (GDC) data portal [79]. The GMKF Neuroblastoma dataset is available in dbGAP under phs001436.v1.p1 [8]. The PPTC dataset is available in dbGAP under phs001437.v2.p1 [13]. The Pediatric Brain Tumor Atlas data (PBTA), containing the subcohorts OpenPBTA, Kids First PBTA (X01), Chordoma Foundation, MI-ONCOSEQ Study, PNOC, and DGD, are available in dbGAP under phs002517.v4.p2 [9] or in the Kids First Portal at https://kidsfirstdrc.org [4]. The raw Genotype-Tissue Expression (GTEx) dataset is available in dbGAP under phs000424.v9.p2 and processed data are publicly available at https://gtexportal.org/home [16]. The Cancer Genome Atlas (TCGA) dataset is available in dbGAP under phs000178.v11.p8 [17], and processed somatic data are openly available at the NCI GDC data portal [79]. Raw CBTN proteomics data are available under PDC000180 and PDC000176 and adult GBM datasets under PDC000204, PDC000446, PDC000205, and PDC000448 from the Proteomic Data Commons [80]. Merged summary files for the latest release of OpenPedCan are openly accessible in CAVATICA [37] or via download-data.sh script in the OpenPedCan-analysis repository [77]. Cancer group summary data from release v12 are visible within the NCI’s pediatric Molecular Targets Platform [1]. Cohort, cancer group, and individual data are visible within PedcBioPortal [81].

## References

[1] Molecular Targets Platform . https://moleculartargets.ccdi.cancer.gov/. Accessed 14 July 2025.

[2] Children's Brain Tumor Network . https://cbtn.org/. Accessed 14 July 2025.

[3] Lilly JV, Rokita JL, Mason JL, et al. The Children's Brain Tumor Network (CBTN)—accelerating research in pediatric central nervous system tumors through collaboration and open science. Neoplasia. 2023;35:100846. 10.1016/j.neo.2022.100846. 36335802 PMC9641002

[4] Gabriella Kids First Pediatric Research Program Data Resource Center . https://kidsfirstdrc.org/. Accessed 14 July 2025.

[5] Pediatric Neuro-Oncology Consortium . https://pnoc.us/. Accessed 14 July 2025.

[6] Shapiro JA, Gaonkar KS, Spielman SJ, et al. OpenPBTA: the Open Pediatric Brain Tumor Atlas. Cell Genomics. 2023;3(7):100340. 10.1016/j.xgen.2023.100340. 37492101 PMC10363844

[7] National Cancer Institute (NCI) . TARGET: Therapeutically Applicable Research to Generate Effective Treatments dbGaP Study. https://www.ncbi.nlm.nih.gov/projects/gap/cgi-bin/study.cgi?study_id=phs000218.v23.p8. Accessed 14 July 2025.

[8] Discovering the genetic basis of human neuroblastoma: a Gabriella Miller Kids First Pediatric Research Program (Kids First) Project dbGaP Study. https://www.ncbi.nlm.nih.gov/projects/gap/cgi-bin/study.cgi?study_id=phs001436.v1.p1. Accessed 14 July 2025.

[9] Childhood Cancer Data Initiative (CCDI) . Molecular characterization across pediatric brain tumors and other solid and hematologic malignancies for research, diagnostic, and precision medicine dbGaP study. https://www.ncbi.nlm.nih.gov/projects/gap/cgi-bin/study.cgi?study_id=phs002517.v2.p2. Accessed 14 July 2025.

[10] Chordoma Foundation . https://www.chordomafoundation.org/. Accessed 14 July 2025.

[11] Pediatric Preclinical In Vivo Testing Consortium (PIVOT) . https://preclinicalpivot.org/about-pivot/. Accessed 14 July 2025.

[12] Rokita JL, Rathi KS, Cardenas MF, et al. Genomic profiling of childhood tumor patient-derived xenograft models to enable rational clinical trial design. Cell Rep. 2019;29(6):1675–89. 10.1016/j.celrep.2019.09.071. 31693904 PMC6880934

[13] Pediatric Preclinical Testing Consortium (PPTC) dbGaP Study . https://www.ncbi.nlm.nih.gov/projects/gap/cgi-bin/study.cgi?study_id=phs001437.v2.p1. Accessed 14 July 2025.

[14] Harenza JL, Diamond MA, Adams RN, et al. Transcriptomic profiling of 39 commonly-used neuroblastoma cell lines. Sci Data. 2017;4:170033. 10.1038/sdata.2017.33. 28350380 PMC5369315

[15] Michigan Center for Translational Pathology . https://mctp.med.umich.edu. Accessed 14 July 2025.

[16] The Genotype Tissue Expression (GTEx) Portal . https://gtexportal.org/home. Accessed 14 July 2025.

[17] National Institutes of Health. The Cancer Genome Atlas (TCGA) dbGaP Study . https://www.ncbi.nlm.nih.gov/projects/gap/cgi-bin/study.cgi?study_id=phs000178.v11.p8 Accessed 14 July 2025.

[18] Petralia F, Tignor N, Reva B, et al. Integrated proteogenomic characterization across major histological types of pediatric brain cancer. Cell. 2020;183(7):1962–85. 10.1016/j.cell.2020.10.044. 33242424 PMC8143193

[19] Wang L-B, Karpova A, Gritsenko MA, et al. Proteogenomic and metabolomic characterization of human glioblastoma. Cancer Cell. 2021; 39(4):509–28.e20. 10.1016/j.ccell.2021.01.006. 33577785 PMC8044053

[20] Kids First Tumor Only Workflow v0.3.0-beta . https://github.com/kids-first/kf-tumor-workflow/tree/v0.3.0-beta. Accessed 14 July 2025.

[21] McLaren W, Gil L, Hunt SE, et al. The Ensembl variant effect predictor. Genome Biol. 2016;17:122. 10.1186/s13059-016-0974-4. 27268795 PMC4893825

[22] Kim S, Scheffler K, Halpern AL, et al. Strelka2: fast and accurate calling of germline and somatic variants. Nat Methods. 2018; 15:591–94. 10.1038/s41592-018-0051-x. 30013048

[23] Benjamin D, Sato T, Cibulskis K, et al. Calling somatic SNVs and indels with Mutect2. bioRxiv. 2019. 10.1101/861054. Accessed 01 May 2022.

[24] Narzisi G, Corvelo A, Arora K, et al. Genome-wide somatic variant calling using localized colored de Bruijn graphs. Commun Biol. 2018;1:20. 10.1038/s42003-018-0023-9. 30271907 PMC6123722

[25] Lai Z, Markovets A, Ahdesmaki M, et al. VarDict: a novel and versatile variant caller for next-generation sequencing in cancer research. Nucleic Acids Res. 2016;44(11):20. 10.1093/nar/gkw227. PMC4914105 27060149

[26] Boeva V, Popova T, Bleakley K, et al. Control-FREEC: a tool for assessing copy number and allelic content using next-generation sequencing data. Bioinformatics. 2011; 28(3):423–25. 10.1093/bioinformatics/btr670. 22155870 PMC3268243

[27] Boeva V, Zinovyev A, Bleakley K, et al. Control-free calling of copy number alterations in deep-sequencing data using GC-content normalization. Bioinformatics. 2010;27(2):268–69. 10.1093/bioinformatics/btq635. 21081509 PMC3018818

[28] Talevich E, Shain AH, Botton T, et al. Genome-wide copy number detection and visualization from targeted DNA sequencing. PLoS Comput Biol. 2016;12(4):e1004873. 10.1371/journal.pcbi.1004873. 27100738 PMC4839673

[29] McKenna A, Hanna M, Banks E, et al. The Genome Analysis Toolkit: a MapReduce framework for analyzing next-generation DNA sequencing data. Genome Res. 2010; 20:1297–1303. 10.1101/gr.107524.110. 20644199 PMC2928508

[30] Kids First AWS SNP hg38 reference file. https://kids-first-seq-data.s3.amazonaws.com/pipeline_references/dbSNP_v153_ucsc-compatible.converted.vt.decomp.norm.common_snps.vcf.gz. Accessed 14 July 2025.

[31] Kids First AWS hg38 mappability reference file. https://s3.amazonaws.com/kids-first-seq-data/pipeline_references/hg38_canonical_150.mappability. Accessed 14 July 2025.

[32] Kids First CAVATICA reference project. https://cavatica.sbgenomics.com/u/kfdrc-harmonization/kf-references. Accessed 14 July 2025.

[33] Kids First GitHub repository for ControlFREEC tumor only workflow. https://github.com/kids-first/kf-tumor-workflow/blob/v0.3.0-beta/workflows/kfdrc_controlfreec_tumor_only_wf.cwl. Accessed 14 July 2025.

[34] Chen X, Schulz-Trieglaff O, Shaw R, et al. Manta: rapid detection of structural variants and indels for germline and cancer sequencing applications. Bioinformatics. 2015;32(8):1220–22. 10.1093/bioinformatics/btv710. 26647377

[35] Geoffroy V, Herenger Y, Kress A, et al. AnnotSV: an integrated tool for structural variations annotation. Bioinformatics. 2018;34(20):3572–74. 10.1093/bioinformatics/bty304. 29669011

[36] Love MI, Huber W, Anders S. Moderated estimation of fold change and dispersion for RNA-seq data with DESeq2. Genome Biol. 2014;15:550. 10.1186/s13059-014-0550-8. 25516281 PMC4302049

[37] OpenPedCan CAVATICA Project release v15. https://cavatica.sbgenomics.com/u/cavatica/opentarget/files/#q?path=v15. Accessed 14 July 2025.

[38] Gene Symbol Conversion Python Script. https://github.com/d3b-center/D3b-DGD-Collaboration/blob/d7ca30291dfc705639c42cd51e7a92dc32cc9096/scripts/update_gene_symbols.py. Accessed 14 July 2025.

[39] The HGNC Database. https://ftp.ebi.ac.uk/pub/databases/genenames/hgnc/archive/monthly/tsv/hgnc_complete_set_2021-06-01.txt. Accessed 14 July 2025.

[40] Uhrig S, Ellermann J, Walther T, et al. Accurate and efficient detection of gene fusions from RNA sequencing data. Genome Res. 2021;31:448–60. 10.1101/gr.257246.119. 33441414 PMC7919457

[41] Haas BJ, Dobin A, Stransky N, et al. STAR-Fusion: fast and accurate fusion transcript detection from RNA-seq. bioRxiv. 2017. 10.1101/120295. Accessed 10 May 2023.

[42] Rokita J, Marini F, Gaonkar K, et al. AnnoFuseData: v1.0.0. Zenodo. 10.5281/zenodo.13152566. Accessed 01 May 2022.

[43] Gaonkar KS, Marini F, Rathi KS, et al. annoFuse: an R package to annotate, prioritize, and interactively explore putative oncogenic RNA fusions. BMC Bioinf. 2020;21:577. 10.1186/s12859-020-03922-7. PMC7737294 33317447

[44] Marini F, Gaonkar K, Rokita JL. annoFuse v0.90.0. Zenodo. 2020. 10.5281/zenodo.4036788.

[45] Children's Hospital of Philadelphia Test Directory. https://www.testmenu.com/chop/Tests/785504. Accessed 14 July 2025.

[46] Kids First GitHub repository for rMATs workflow. https://github.com/kids-first/kf-rnaseq-workflow/blob/5d9dd57011df312e3ecdea4e64d71e24ed38ac12/workflow/rmats_wf.cwl. Accessed 14 July 2025.

[47] Kong AT, Leprevost FV, Avtonomov DM, et al. MSFragger: ultrafast and comprehensive peptide identification in mass spectrometry–based proteomics. Nat Methods. 2017;14:513–20. 10.1038/nmeth.4256. 28394336 PMC5409104

[48] Gibbons BC, Chambers MC, Monroe ME, et al. Correcting systematic bias and instrument measurement drift with mzRefinery. Bioinformatics. 2015; 31(23):3838–40. 10.1093/bioinformatics/btv437. 26243018 PMC4653383

[49] Kim S, Pevzner PA, 5:5277. MS-GF+ makes progress towards a universal database search tool for proteomics. Nat Commun. 2014; 10.1038/ncomms6277. 25358478 PMC5036525

[50] Liu X, Segar MW, Li SC, et al. Spectral probabilities of top-down tandem mass spectra. BMC Genomics. 2014;15(Suppl 1):S9. 10.1186/1471-2164-15-s1-s9. PMC4046700 24564718

[51] Beausoleil SA, Villén J, Gerber SA, et al. A probability-based approach for high-throughput protein phosphorylation analysis and site localization. Nat Biotechnol. 2006;24:1285–92. 10.1038/nbt1240. 16964243

[52] Fortin J-P, Triche TJ Jr, Hansen KD. Preprocessing, normalization and integration of the Illumina HumanMethylationEPIC array with minfi. Bioinformatics. 2016;33(4):558–60. 10.1093/bioinformatics/btw691. PMC5408810 28035024

[53] Wilhelm-Benartzi CS, Koestler DC, Karagas MR, et al. Review of processing and analysis methods for DNA methylation array data. Br J Cancer. 2013;109:1394–1402. 10.1038/bjc.2013.496. 23982603 PMC3777004

[54] Daca-Roszak P, Pfeifer A, Żebracka-Gala J, et al. Impact of SNPs on methylation readouts by Illumina Infinium HumanMethylation450 BeadChip Array: implications for comparative population studies. BMC Genomics. 2015;16:1003. 10.1186/s12864-015-2202-0. 26607064 PMC4659175

[55] Capper D, Jones DTW, Sill M, et al. DNA methylation-based classification of central nervous system tumours. Nature. 2018;555:469–74. 10.1038/nature26000. 29539639 PMC6093218

[56] Liberzon A, Birger C, Thorvaldsdóttir H, et al. The molecular signatures database hallmark gene set collection. Cell Syst. 2015;1(6):417–25. 10.1016/j.cels.2015.12.004. 26771021 PMC4707969

[57] Hänzelmann S, Castelo R, Guinney J. GSVA: gene set variation analysis for microarray and RNA-seq data. BMC Bioinf. 2013;14:7. 10.1186/1471-2105-14-7. PMC3618321 23323831

[58] Mermel CH, Schumacher SE, Hill B, et al. GISTIC2.0 facilitates sensitive and confident localization of the targets of focal somatic copy-number alteration in human cancers. Genome Biol. 2011;12:R41. 10.1186/gb-2011-12-4-r41. 21527027 PMC3218867

[59] Rosenthal R, McGranahan N, Herrero J, et al. deconstructSigs: delineating mutational processes in single tumors distinguishes DNA repair deficiencies and patterns of carcinoma evolution. Genome Biol. 2016;17:31. 10.1186/s13059-016-0893-4. 26899170 PMC4762164

[60] Alexandrov LB, Nik-Zainal S, Wedge DC, et al. Signatures of mutational processes in human cancer. Nature. 2013;500:415–421. 10.1038/nature12477. 23945592 PMC3776390

[61] Stenzinger A, Allen JD, Maas J, et al. Tumor mutational burden standardization initiatives: recommendations for consistent tumor mutational burden assessment in clinical samples to guide immunotherapy treatment decisions. Genes Chromosomes Cancer. 2019;58(8):578–88. 10.1002/gcc.22733. 30664300 PMC6618007

[62] Kids First GitHub repository for Somatic Consensus Variant Calling workflow. https://github.com/kids-first/kf-somatic-workflow/blob/9ce392106e7cbbda569842cda1751d0b29225c83/docs/kfdrc-consensus-calling.md. Accessed 14 July 2025.

[63] Vega DM, Yee LM, McShane LM, et al. Aligning tumor mutational burden (TMB) quantification across diagnostic platforms: phase II of the Friends of Cancer Research TMB Harmonization Project. Ann Oncol. 2021;32(12):1626–36. 10.1016/j.annonc.2021.09.016. 34606929

[64] Louis DN, Perry A, Wesseling P, et al. The 2021 WHO Classification of Tumors of the Central Nervous System: a summary. Neurooncology. 2021; 23(8):1231–51. 10.1093/neuonc/noab106. PMC8328013 34185076

[65] Guerreiro Stucklin AS, Ryall S, Fukuoka K, et al. Alterations in ALK/ROS1/NTRK/MET drive a group of infantile hemispheric gliomas. Nat Commun. 2019;10:4343. 10.1038/s41467-019-12187-5. 31554817 PMC6761184

[66] Ho B, Johann PD, Grabovska Y, et al. Molecular subgrouping of atypical teratoid/rhabdoid tumors—a reinvestigation and current consensus. Neurooncology. 2019;22(5):613–24. 10.1093/neuonc/noz235. PMC7229260 31889194

[67] Rathi KS, Arif S, Koptyra M, et al. A transcriptome-based classifier to determine molecular subtypes in medulloblastoma. PLoS Comput Biol. 2020; 16(10):e1008263. 10.1371/journal.pcbi.1008263. 33119584 PMC7654754

[68] Knijnenburg TA, Wang L, Zimmermann MT, et al. Genomic and molecular landscape of DNA damage repair deficiency across the cancer genome atlas. Cell Rep. 2018;23(1):239–254.e6. 10.1016/j.celrep.2018.03.076. 29617664 PMC5961503

[69] Chang MT, Bhattarai TS, Schram AM, et al. Accelerating discovery of functional mutant alleles in cancer. Cancer Discov. 2018;8(2):174–183. 10.1158/2159-8290.cd-17-0321. 29247016 PMC5809279

[70] Chang MT, Asthana S, Gao SP, et al. Identifying recurrent mutations in cancer reveals widespread lineage diversity and mutational specificity. Nat Biotechnol. 2015;34:155–163. 10.1038/nbt.3391. 26619011 PMC4744099

[71] Harms KL, Chen X. The functional domains in p53 family proteins exhibit both common and distinct properties. Cell Death Differ. 2006;13:890–897. 10.1038/sj.cdd.4401904. 16543939

[72] Dittmer D, Pati S, Zambetti G, et al. Gain of function mutations in p53. Nat Genet. 1993;4:42–46. 10.1038/ng0593-42. 8099841

[73] Guha T, Malkin D. Inherited *TP53* mutations and the Li–Fraumeni syndrome. Cold Spring Harb Perspect Med. 2017;7(4):a026187. 10.1101/cshperspect.a026187. 28270529 PMC5378014

[74] Lee S, Lee S, Ouellette S, et al. NGSCheckMate: software for validating sample identity in next-generation sequencing studies within and across data types. Nucleic Acids Res. 2017;45(11):e103. 10.1093/nar/gkx193. 28369524 PMC5499645

[75] Pedersen BS, Bhetariya PJ, Brown J, et al. Somalier: rapid relatedness estimation for cancer and germline studies using efficient genome sketches. Genome Med. 2020;12:62. 10.1186/s13073-020-00761-2. 32664994 PMC7362544

[76] Learned K, Durbin A, Currie R, et al. Barriers to accessing public cancer genomic data. Sci Data. 2019;6:98. 10.1038/s41597-019-0096-4. 31222016 PMC6586850

[77] OpenPedCan-analysis GitHub Repository. d3b-center/OpenPedCan-analysis. Accessed 14 July 2025.

[78] Rokita J, Wafula E, Jin R, et al., Accessed 2025 July 20 OpenPedCan-analysis GitHub repository v2.5.1. Zenodo. 10.5281/zenodo.6473912.

[79] The National Cancer Institute Genomic Data Commons Portal . https://portal.gdc.cancer.gov/. Accessed 14 July 2025.

[80] National Cancer Institute Proteomic Data Commons . https://proteomic.datacommons.cancer.gov/pdc/. Accessed 14 July 2025.

[81] PedcBioPortal for Integrated Childhood Cancer Genomics . https://pedcbioportal.org. Accessed 14 July 2025.

