# Supplementary material for: The Open Pediatric Cancer Project
Source: Gigascience. 2025 Sep 2;14:giaf093. doi: 10.1093/gigascience/giaf093 (PMC12402770; doi:10.1093/gigascience/giaf093)
Supplement: giaf093_GIGA-D-25-00086_Original_Submission [file giaf093_giga-d-25-00086_original_submission.pdf]

GigaScience
The Open Pediatric Cancer Project
--Manuscript Draft--

**Manuscript Number:**	GIGA-D-25-00086	
**Full Title:**	The Open Pediatric Cancer Project	
**Article Type:**	Data Note	
**Funding Information:**	National Cancer Institute (75N91019D00024)	Dr. Deanne Taylor
**Abstract:**	Background: In 2019, the Open Pediatric Brain Tumor Atlas (OpenPBTA) was created as a global, collaborative open-science initiative to genomically characterize 1,074 pediatric brain tumors and 22 patient-derived cell lines. Here, we present an extension of the OpenPBTA called the Open Pediatric Cancer (OpenPedCan) Project, a harmonized open-source multi-omic dataset from 6,112 pediatric cancer patients with 7,096 tumor events across more than 100 histologies. Combined with RNA-Seq from the Genotype-Tissue Expression (GTEx) and The Cancer Genome Atlas (TCGA), OpenPedCan contains nearly 48,000 total biospecimens (24,002 tumor and 23,893 normal specimens).<br>Findings: We utilized Gabriella Miller Kids First (GMKF) workflows to harmonize WGS, WXS, RNA-seq, and Targeted Sequencing datasets to include somatic SNVs, InDels, CNVs, SVs, RNA expression, fusions, and splice variants. We integrated summarized CPTAC whole cell proteomics and phospho-proteomics data, miRNA-Seq data, and have developed a methylation array harmonization workflow to include m-values, beta-vales, and copy number calls. OpenPedCan contains reproducible, dockerized workflows in GitHub, CAVATICA, and Amazon Web Services (AWS) to deliver harmonized and processed data from over 60 scalable modules which can be leveraged both locally and on AWS. The processed data are released in a versioned manner and accessible through CAVATICA or AWS S3 download (from GitHub), and queryable through PedcBioPortal and the NCI's pediatric Molecular Targets Platform. Notably, we have expanded PBTA molecular subtyping to include methylation information to align with the WHO 2021 Central Nervous System Tumor classifications, allowing us to create research-grade integrated diagnoses for these tumors.<br>Conclusions: OpenPedCan data and its reproducible analysis module framework are openly available and can be utilized and/or adapted by researchers to accelerate discovery, validation, and clinical translation.	
**Corresponding Author:**	Jo Lynne Rokita, Ph.D.<br>Children's National Hospital<br>UNITED STATES	
**Corresponding Author Secondary Information:**		
**Corresponding Author's Institution:**	Children's National Hospital	
**Corresponding Author's Secondary Institution:**		
**First Author:**	Zhuangzhuang Geng	
**First Author Secondary Information:**		
**Order of Authors:**	Zhuangzhuang Geng	
	Eric Wafula	
	Ryan J. Corbett	
	Yuanchao Zhang	
	Run Jin	
	Krutika S. Gaonkar	
	Sangeeta Shukla	

Powered by Editorial Manager® and ProduXion Manager® from Aries Systems Corporation

	Komal S. Rathi
	Dave Hill
	Aditya Lahiri
	Daniel P. Miller
	Alex Sickler
	Kelsey Keith
	Christopher Blackden
	Antonia Chroni
	Miguel A. Brown
	Adam A. Kraya
	Brian R. Rood
	Adam C. Resnick
	Nicholas Van Kuren
	John M. Maris
	Alvin Farrel
	Mateusz P. Koptyra
	Gerri R. Trooskin
	Noel Coleman
	Yuankun Zhu
	Stephanie Stefankiewicz
	Zied Abdullaev
	Asif Chinwalla
	Mariarita Santi
	Ammar S. Naqvi
	Jennifer L. Mason
	Carl J. Koschmann
	Xiaoyan Huang
	Sharon J. Diskin
	Kenneth Aldape
	Bailey K. Farrow
	Weiping Ma
	Bo Zhang
	Brian M. Ennis
	Sarah Tasian
	Saksham Phul
	Matthew R. Leuder
	Chuwei Zhong
	Joseph M. Dybas
	Pei Wang
	Deanne Taylor
	Jo Lynne Rokita, Ph.D.

Powered by Editorial Manager® and ProduXion Manager® from Aries Systems Corporation

Order of Authors Secondary Information:	
**Additional Information:**	
**Question**	**Response**
Are you submitting this manuscript to a special series or article collection?	No
**Experimental design and statistics**<br><br>Full details of the experimental design and statistical methods used should be given in the Methods section, as detailed in our Minimum Standards Reporting Checklist. Information essential to interpreting the data presented should be made available in the figure legends.<br><br>Have you included all the information requested in your manuscript?	Yes
**Resources**<br><br>A description of all resources used, including antibodies, cell lines, animals and software tools, with enough information to allow them to be uniquely identified, should be included in the Methods section. Authors are strongly encouraged to cite Research Resource Identifiers (RRIDs) for antibodies, model organisms and tools, where possible.<br><br>Have you included the information requested as detailed in our Minimum Standards Reporting Checklist?	Yes
**Availability of data and materials**<br><br>All datasets and code on which the conclusions of the paper rely must be either included in your submission or deposited in publicly available repositories (where available and ethically appropriate), referencing such data using a unique identifier in the references and in the "Availability of Data and Materials" section of your manuscript.	Yes

Have you have met the above requirement as detailed in our Minimum Standards Reporting Checklist?	

Manuscript

Click here to access/download;Manuscript;Geng_et_al_OpenPedCan_manus

The Open Pediatric Cancer Project

Authors

- **Zhuangzhuang Geng** 0009-0007-6883-0691 · zzgeng Center for Data-Driven Discovery in Biomedicine, Children's Hospital of Philadelphia, Philadelphia, PA, 19104, USA; Division of Neurosurgery, Children's Hospital of Philadelphia, Philadelphia, PA, 19104, USA

- **Eric Wafula** 0000-0001-8073-3797 · ewafula Department of Biomedical and Health Informatics, Children's Hospital of Philadelphia, Philadelphia, PA, 19104, USA

- **Ryan J. Corbett** 0000-0002-3478-0784 · rjcorb Center for Cancer and Immunology Research, Children's National Hospital, Washington, DC, 20010, USA; Center for Data-Driven Discovery in Biomedicine, Children's Hospital of Philadelphia, Philadelphia, PA, 19104, USA; Division of Neurosurgery, Children's Hospital of Philadelphia, Philadelphia, PA, 19104, USA

- **Yuanchao Zhang** Department of Biomedical and Health Informatics, Children's Hospital of Philadelphia, Philadelphia, PA, 19104, USA

- **Run Jin** 0000-0002-8958-9266 · runjin326 Center for Data-Driven Discovery in Biomedicine, Children's Hospital of Philadelphia, Philadelphia, PA, 19104, USA; Division of Neurosurgery, Children's Hospital of Philadelphia, Philadelphia, PA, 19104, USA

- **Krutika S. Gaonkar** 0000-0003-0838-2405 Center for Data-Driven Discovery in Biomedicine, Children’s Hospital of Philadelphia, Philadelphia, PA, 19104, USA; Division of Neurosurgery, Children’s Hospital of Philadelphia, Philadelphia, PA, 19104, USA; Department of Biomedical and Health Informatics, Children’s Hospital of Philadelphia, Philadelphia, PA, 19104, USA
- **Sangeeta Shukla** 0000-0002-3727-9602 · sangeetashukla Department of Biomedical and Health Informatics, Children’s Hospital of Philadelphia, Philadelphia, PA, 19104, USA
- **Komal S. Rathi** 0000-0001-5534-6904 · komalsrathi Center for Data-Driven Discovery in Biomedicine, Children’s Hospital of Philadelphia, Philadelphia, PA, 19104, USA; Department of Biomedical and Health Informatics, Children’s Hospital of Philadelphia, Philadelphia, PA, 19104, USA
- **Dave Hill** 0000-0002-1337-1789 · atlas4213 Department of Biomedical and Health Informatics, Children’s Hospital of Philadelphia, Philadelphia, PA, 19104, USA
- **Aditya Lahiri** 0000-0001-9352-1312 · adilahiri Department of Biomedical and Health Informatics, Children’s Hospital of Philadelphia, Philadelphia, PA, 19104, USA
- **Daniel P. Miller** 0000-0002-2032-4358 · dmiller15 Center for Data-Driven Discovery in Biomedicine, Children’s Hospital of Philadelphia, Philadelphia, PA, 19104, USA; Division of Neurosurgery, Children’s Hospital of Philadelphia, Philadelphia, PA, 19104, USA

- **Alex Sickler** 0000-0001-7830-7537 · sickler-alex Center for Cancer and Immunology Research, Children's National Hospital, Washington, DC, 20010, USA; Center for Data-Driven Discovery in Biomedicine, Children's Hospital of Philadelphia, Philadelphia, PA, 19104, USA; Division of Neurosurgery, Children's Hospital of Philadelphia, Philadelphia, PA, 19104, USA

- **Kelsey Keith** 0000-0002-7451-5117 · kelseykeith Department of Biomedical and Health Informatics, Children's Hospital of Philadelphia, Philadelphia, PA, 19104, USA

- **Christopher Blackden** 0000-0002-4928-090X · devbyaccident Center for Data-Driven Discovery in Biomedicine, Children's Hospital of Philadelphia, Philadelphia, PA, 19104, USA; Division of Neurosurgery, Children's Hospital of Philadelphia, Philadelphia, PA, 19104, USA

- **Antonia Chroni** · AntoniaChroni Center for Data-Driven Discovery in Biomedicine, Children's Hospital of Philadelphia, Philadelphia, PA, 19104, USA; Division of Neurosurgery, Children's Hospital of Philadelphia, Philadelphia, PA, 19104, USA

- **Miguel A. Brown** 0000-0001-6782-1442 · migbro Center for Data-Driven Discovery in Biomedicine, Children's Hospital of Philadelphia, Philadelphia, PA, 19104, USA; Division of Neurosurgery, Children's Hospital of Philadelphia, Philadelphia, PA, 19104, USA

- **Adam A. Kraya** 0000-0002-8526-5694 · aadamk Center for Data-Driven Discovery in Biomedicine, Children's Hospital of Philadelphia, Philadelphia, PA, 19104,

USA; Division of Neurosurgery, Children’s Hospital of Philadelphia, Philadelphia, PA, 19104, USA

- **Brian R. Rood** Children’s National Research Institute, Washington, D.C.; George Washington University School of Medicine and Health Sciences, Washington, D.C., 20052, USA

- **Adam C. Resnick** 0000-0003-0436-4189 · adamcresnick Center for Data-Driven Discovery in Biomedicine, Children’s Hospital of Philadelphia, Philadelphia, PA, 19104, USA; Division of Neurosurgery, Children’s Hospital of Philadelphia, Philadelphia, PA, 19104, USA · Funded by Children’s Brain Tumor Network; NIH 3P30 CA016520-44S5, U2C HL138346-03, U24 CA220457-03; NCI/NIH Contract No. 75N91019D00024, Task Order No. 75N91020F00003; Children’s Hospital of Philadelphia Division of Neurosurgery

- **Nicholas Van Kuren** 0000-0002-7414-9516 · nicholasvk Center for Data-Driven Discovery in Biomedicine, Children’s Hospital of Philadelphia, Philadelphia, PA, 19104, USA; Division of Neurosurgery, Children’s Hospital of Philadelphia, Philadelphia, PA, 19104, USA

- **John M. Maris** Division of Oncology, Children’s Hospital of Philadelphia, Philadelphia, PA, 19104, USA; Department of Pediatrics, University of Pennsylvania, Philadelphia, PA, 19104, USA

- **Alvin Farrel** 0000-0003-1087-9840 · afarrel Department of Biomedical and Health Informatics, Children’s Hospital of Philadelphia, Philadelphia, PA, 19104, USA; Division of Oncology, Children’s Hospital of Philadelphia, Philadelphia, PA, 19104, USA; Center for Childhood Cancer Research, Children’s Hospital of Philadelphia,

Philadelphia, PA, 19104, USA · Funded by NCI/NIH Contract No. 75N91019D00024, Task Order No. 75N91020F00003

- **Mateusz P. Koptyra** 0000-0002-3857-6633 · mkoptyra Center for Data-Driven Discovery in Biomedicine, Children's Hospital of Philadelphia, Philadelphia, PA, 19104, USA; Division of Neurosurgery, Children's Hospital of Philadelphia, Philadelphia, PA, 19104, USA

- **Gerri R. Trooskin** Center for Data-Driven Discovery in Biomedicine, Children's Hospital of Philadelphia, Philadelphia, PA, 19104, USA; Division of Neurosurgery, Children's Hospital of Philadelphia, Philadelphia, PA, 19104, USA

- **Noel Coleman** 0000-0001-6454-1285 Center for Data-Driven Discovery in Biomedicine, Children's Hospital of Philadelphia, Philadelphia, PA, 19104, USA; Division of Neurosurgery, Children's Hospital of Philadelphia, Philadelphia, PA, 19104, USA

- **Yuankun Zhu** 0000-0002-2455-9525 · yuankunzhu Center for Data-Driven Discovery in Biomedicine, Children's Hospital of Philadelphia, Philadelphia, PA, 19104, USA; Division of Neurosurgery, Children's Hospital of Philadelphia, Philadelphia, PA, 19104, USA

- **Stephanie Stefankiewicz** Center for Data-Driven Discovery in Biomedicine, Children's Hospital of Philadelphia, Philadelphia, PA, 19104, USA; Division of Neurosurgery, Children's Hospital of Philadelphia, Philadelphia, PA, 19104, USA

- **Zied Abdullaev** Laboratory of Pathology, National Cancer Institute, Bethesda, MD, 20892, USA

- **Asif T Chinwalla** 0000-0001-7831-3996 · chinwallaa Department of Biomedical and Health Informatics, Children's Hospital of Philadelphia, Philadelphia, PA, 19104, USA

- **Mariarita Santi** 0000-0002-6728-3450 Department of Pathology and Laboratory Medicine, Children's Hospital of Philadelphia, Philadelphia, PA, 19104, USA; Department of Pathology and Laboratory Medicine, University of Pennsylvania Perelman School of Medicine, Philadelphia, PA, 19104, USA

- **Ammar S. Naqvi** · naqvia Center for Data-Driven Discovery in Biomedicine, Children's Hospital of Philadelphia, Philadelphia, PA, 19104, USA; Division of Neurosurgery, Children's Hospital of Philadelphia, Philadelphia, PA, 19104, USA

- **Jennifer L. Mason** Center for Data-Driven Discovery in Biomedicine, Children's Hospital of Philadelphia, Philadelphia, PA, 19104, USA; Division of Neurosurgery, Children's Hospital of Philadelphia, Philadelphia, PA, 19104, USA

- **Carl J. Koschmann** 0000-0002-0825-7615 Department of Pediatrics, University of Michigan Health, Ann Arbor, MI, 48105, USA; Pediatric Hematology Oncology, Mott Children's Hospital, Ann Arbor, MI, 48109, USA

- **Xiaoyan Huang** 0000-0001-7267-4512 · HuangXiaoyan0106 Center for Data-Driven Discovery in Biomedicine, Children's Hospital of Philadelphia, Philadelphia, PA, 19104, USA; Division of Neurosurgery, Children's Hospital of Philadelphia, Philadelphia, PA, 19104, USA

- **Sharon J. Diskin** 0000-0002-7200-8939 Division of Oncology, Children's Hospital of Philadelphia, Philadelphia, PA, 19104, USA; Department of Pediatrics, University of Pennsylvania, Philadelphia, PA, 19104, USA
- **Kenneth Aldape** Laboratory of Pathology, National Cancer Institute, Bethesda, MD, 20892, USA
- **Bailey K. Farrow** 0000-0001-6727-6333 · baileyckelly Center for Data-Driven Discovery in Biomedicine, Children's Hospital of Philadelphia, Philadelphia, PA, 19104, USA; Division of Neurosurgery, Children's Hospital of Philadelphia, Philadelphia, PA, 19104, USA
- **Weiping Ma** Department of Genetics and Genomic Sciences, Icahn School of Medicine at Mount Sinai, New York, NY 10029, USA; Tisch Cancer Institute, Icahn School of Medicine at Mount Sinai, New York, NY 10029, USA
- **Bo Zhang** 0000-0002-0743-5379 · zhangb1 Center for Data-Driven Discovery in Biomedicine, Children's Hospital of Philadelphia, Philadelphia, PA, 19104, USA; Division of Neurosurgery, Children's Hospital of Philadelphia, Philadelphia, PA, 19104, USA
- **Brian M. Ennis** 0000-0002-2653-5009 · bmennis Center for Data-Driven Discovery in Biomedicine, Children's Hospital of Philadelphia, Philadelphia, PA, 19104, USA; Division of Neurosurgery, Children's Hospital of Philadelphia, Philadelphia, PA, 19104, USA

- **Sarah Tasian** Division of Oncology, Children's Hospital of Philadelphia, Philadelphia, PA, 19104, USA; Department of Pediatrics, University of Pennsylvania, Philadelphia, PA, 19104, USA

- **Saksham Phul** 0000-0002-2771-2572 · sakshamphul Center for Data-Driven Discovery in Biomedicine, Children's Hospital of Philadelphia, Philadelphia, PA, 19104, USA; Division of Neurosurgery, Children's Hospital of Philadelphia, Philadelphia, PA, 19104, USA

- **Matthew R. Lueder** 0009-0002-7370-102X · luederm Center for Data-Driven Discovery in Biomedicine, Children's Hospital of Philadelphia, Philadelphia, PA, 19104, USA; Division of Neurosurgery, Children's Hospital of Philadelphia, Philadelphia, PA, 19104, USA; Department of Pathology and Laboratory Medicine, Children's Hospital of Philadelphia, Philadelphia, PA, 19104, USA

- **Chuwei Zhong** 0000-0003-2406-2735 · zoomzoom1011 Center for Data-Driven Discovery in Biomedicine, Children's Hospital of Philadelphia, Philadelphia, PA, 19104, USA; Division of Neurosurgery, Children's Hospital of Philadelphia, Philadelphia, PA, 19104, USA

- **Joseph M. Dybas** · JosephDybas Center for Data-Driven Discovery in Biomedicine, Children's Hospital of Philadelphia, Philadelphia, PA, 19104, USA; Division of Neurosurgery, Children's Hospital of Philadelphia, Philadelphia, PA, 19104, USA

- **Pei Wang** Department of Genetics and Genomic Sciences, Icahn School of Medicine at Mount Sinai, New York, NY 10029, USA; Tisch Cancer Institute, Icahn School of Medicine at Mount Sinai, New York, NY 10029, USA

- **Deanne Taylor** 0000-0002-3302-4610 · taylordm Department of Biomedical and Health Informatics, Children's Hospital of Philadelphia, Philadelphia, PA, 19104, USA; Department of Pediatrics, University of Pennsylvania Perelman Medical School, Philadelphia, PA, 19104, USA · Funded by NCI/NIH Contract No. 75N91019D00024, Task Order No. 75N91020F00003

- **Jo Lynne Rokita** ✉ 0000-0003-2171-3627 · jharenza Center for Cancer and Immunology Research, Children's National Hospital, Washington, DC, 20010, USA; Center for Data-Driven Discovery in Biomedicine, Children's Hospital of Philadelphia, Philadelphia, PA, 19104, USA; Division of Neurosurgery, Children's Hospital of Philadelphia, Philadelphia, PA, 19104, USA; Department of Biomedical and Health Informatics, Children's Hospital of Philadelphia, Philadelphia, PA, 19104, USA · Funded by NCI/NIH Contract No. 75N91019D00024, Task Order No. 75N91020F00003

Contact information

✉Correspondence: Jo Lynne Rokita jrokita@childrensnational.org

Abstract

Background: In 2019, the Open Pediatric Brain Tumor Atlas (OpenPBTA) was created as a global, collaborative open-science initiative to genomically characterize 1,074 pediatric brain tumors and 22 patient-derived cell lines. Here, we present an extension of the OpenPBTA called the Open Pediatric Cancer (OpenPedCan) Project, a harmonized open-source multi-omic dataset from 6,112 pediatric cancer patients with 7,096 tumor events across more than 100 histologies. Combined with RNA-Seq from the Genotype-Tissue Expression (GTEx) and The

Cancer Genome Atlas (TCGA), OpenPedCan contains nearly 48,000 total biospecimens (24,002 tumor and 23,893 normal specimens).

Findings: We utilized Gabriella Miller Kids First (GMKF) workflows to harmonize WGS, WXS, RNA-seq, and Targeted Sequencing datasets to include somatic SNVs, InDels, CNVs, SVs, RNA expression, fusions, and splice variants. We integrated summarized CPTAC whole cell proteomics and phospho-proteomics data, miRNA-Seq data, and have developed a methylation array harmonization workflow to include m-values, beta-vales, and copy number calls. OpenPedCan contains reproducible, dockerized workflows in GitHub, CAVATICA, and Amazon Web Services (AWS) to deliver harmonized and processed data from over 60 scalable modules which can be leveraged both locally and on AWS. The processed data are released in a versioned manner and accessible through CAVATICA or AWS S3 download (from GitHub), and queryable through PedcBioPortal and the NCI's pediatric Molecular Targets Platform. Notably, we have expanded PBTA molecular subtyping to include methylation information to align with the WHO 2021 Central Nervous System Tumor classifications, allowing us to create research-grade integrated diagnoses for these tumors.

Conclusions: OpenPedCan data and its reproducible analysis module framework are openly available and can be utilized and/or adapted by researchers to accelerate discovery, validation, and clinical translation.

Keywords

Pediatric cancer, open science, reproducibility, multi-omics, Docker

Data Description

The Open Pediatric Cancer (OpenPedCan) project is an iterative open analysis effort in which we harmonize pediatric cancer data from multiple sources, perform downstream cancer analyses on these data, and provide them through Amazon S3, CAVATICA, PedcBioPortal, and v2.1 of NCI's [Pediatric Molecular Targets Platform (MTP)](). We harmonized, aggregated, and analyzed data from multiple pediatric and adult data sources, building upon the work of the OpenPBTA (**Figure** **1**). Biospecimen-level metadata and clinical data are contained in **Supplemental Table 1**.

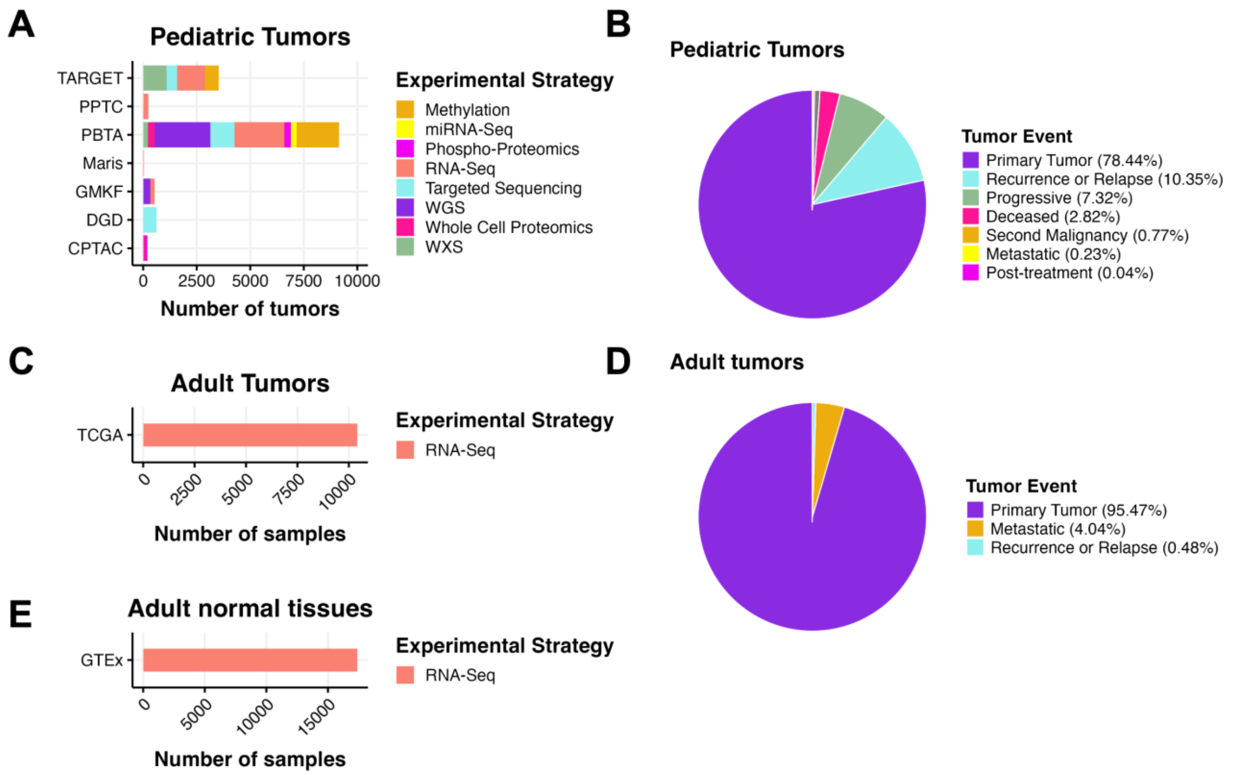

Figure 1: ***OpenPedCan Data.*** *A, OpenPedCan contains multi-omic data from seven cohorts of pediatric tumors (A-B) with counts by tumor event, RNA-Seq from adult tumors from The Cancer Genome Atlas (TCGA) Program (C-D) and RNA-Seq from normal adult tissues from the*

Genotype-Tissue Expression (GTeX) project (E) with counts by specimen. (Abbreviations: TARGET = Therapeutically Applicable Research to Generate Effective Treatments , PPTC = Pediatric Preclinical Testing Consortium, PBTA = Pediatric Brain Tumor Atlas, Maris = Neuroblastoma cell lines from the Maris Laboratory at CHOP, GMKF = Gabriella Miller Kids First, DGD = Division of Genomic Diagnostics at CHOP, CPTAC = Clinical Proteomic Tumor Analysis Consortium)

OpenPedCan currently include the following datasets, described more fully below:

- OpenPBTA
- TARGET
- Kids First Neuroblastoma (X01)
- Kids First PBTA (X01)
- Chordoma Foundation
- PPTC
- Maris
- MI-ONCOSEQ Study
- DGD
- GTEx
- TCGA
- CPTAC PBTA
- CPTAC GBM
- HOPE proteomics

Open Pediatric Brain Tumor Atlas (OpenPBTA)

In September of 2018, the Children's Brain Tumor Network (CBTN) released the Pediatric Brain Tumor Atlas (PBTA), a genomic dataset (whole genome sequencing, whole exome sequencing, RNA sequencing, proteomic, and clinical data) for nearly 1,000 tumors, available from the Gabriella Miller Kids First Portal. In September of 2019, the Open Pediatric Brain Tumor Atlas (OpenPBTA) Project was launched. OpenPBTA was a global open science initiative to comprehensively define the molecular landscape of tumors of 943 patients from the CBTN and the PNOC003 DIPG clinical trial from the Pediatric Pacific Neuro-oncology Consortium through real-time, collaborative analyses and collaborative manuscript writing on GitHub [1]. Additional PBTA data has been, and will be continually added to, OpenPedCan.

Therapeutically Applicable Research to Generate Effective Treatments (TARGET)

The Therapeutically Applicable Research to Generate Effective Treatments (TARGET) Initiative is an NCI-funded collection of disease-specific projects that seeks to identify the genomic changes of pediatric cancers. The overall goal is to collect genomic data to accelerate the development of more effective therapies. OpenPedCan analyses include the seven diseases present in the TARGET dataset: Acute Lymphoblastic Leukemia (ALL), Acute Myeloid Leukemia (AML), Clear cell sarcoma of the kidney, Neuroblastoma, Osteosarcoma, Rhabdoid tumor, and Wilm's Tumor.

Gabriella Miller Kids First (Neuroblastoma) and PBTA

The Gabriella Miller Kids First Pediatric Research Program (Kids First) is a large-scale effort to accelerate research and gene discovery in pediatric cancers and structural birth defects. The program includes whole genome sequencing (WGS) from patients with pediatric cancers and structural birth defects and their families. OpenPedCan analyses include Neuroblastoma and PBTA data from the Kids First projects.

Chordoma Foundation

The Chordoma Foundation seeks to advance research and improve healthcare for patients diagnosed with chordoma and has shared patient and model sequencing data with the CBTN.

Pediatric Preclinical Testing Consortium (PPTC)

The National Cancer Institute's (NCI) former PPTC, now the Pediatric Preclinical in Vivo Testing (PIVOT) Program, molecularly and pharmacologically characterizes cell-derived and patient-derived xenograft (PDX) models. OpenPedCan includes re-harmonized RNA-Seq data for 244 models from the initial PPTC study [2].

MI-ONCOSEQ Study [3]

These clinical sequencing data from the University of Michigan were donated to CBTN and added to the PBTA cohort.

Division of Genomic Diagnostics at Children's Hospital of Philadelphia (DGD)

CHOP's Division of Genomic Diagnostics has partnered with CCDI to add somatic panel sequencing data to OpenPedCan and the Molecular Targets Platform.

The Genotype-Tissue Expression Project (GTEx)

The GTEx project is an ongoing effort to build a comprehensive public data resource and tissue bank to study tissue-specific gene expression, regulation and their relationship with genetic variants. Samples were collected from 54 non-diseased tissue sites across nearly 1000 individuals, primarily for molecular assays including WGS, WXS, and RNA-Seq. OpenPedCan project includes 17,382 GTEx RNA-Seq samples from GTEx v8 release, which span across 31 GTEx groups in the v12 release.

The Cancer Genome Atlas Program (TCGA)

TCGA is a landmark cancer genomics program that molecularly characterized over 20,000 primary cancer and matched normal samples spanning 33 cancer types. It is a joint effort between NCI and the National Human Genome Research Institute. OpenPedCan project includes 10,414 TCGA RNA-Seq samples (716 normal and 9698 tumor) from 33 cancer types.

Clinical Proteomic Tumor Analysis Consortium (CPTAC) PBTA proteomics study

The CPTAC pediatric pan-brain tumor study [4] contains 218 tumors profiled by proteogenomics and are included in OPC.

CPTAC adult GBM proteomics study

This CPTAC adult GBM study [5] contains 99 tumors profiled by proteogenomics and are included in OPC.

Project HOPE proteomics study

Project HOPE is an adolescent and young adult high-grade glioma study (in preparation for publication) that contains 90 tumors profiled by proteogenomics and are included in OPC.

Context

Creation of this dataset had multiple motivations. First, we sought to harmonize, summarize, and contextualize pediatric cancer genomics data among normal tissues (GTEx) and adult cancer tissues (TCGA) to enable the creation of the National Cancer Institute’s Molecular Targets Platform (MTP) at https://moleculartargets.ccdi.cancer.gov/. Next, we created this resource for broad community use to promote rapid reuse and accelerate the discovery of

additional mechanisms contributing to the pathogenesis of pediatric cancers and/or to identify novel candidate therapeutic targets for pediatric cancer.

Similar to OpenPBTA, OpenPedCan operates on a pull request model to accept contributions. We set up continuous integration software via GitHub Actions to confirm the reproducibility of analyses within the project's Docker container. We maintained a data release folder on Amazon S3, downloadable directly from S3 or our open-access CAVATICA project, with merged files for each analysis. As we produced new results, identified data issues, or added additional data, we created new data releases in a versioned manner. The project maintainers have included engineers and scientists from the Children's Hospital of Philadelphia and Children's National Hospital.

Methods

An overview of the OpenPedCan methods is depicted in **Figure 2**. Briefly, most primary harmonization analysis workflows were performed with Kids First pipelines written in Common Workflow Language (CWL) using CAVATICA (detailed below). Alignment and expression quantification for GTEx and TCGA RNA-Seq was performed by the respective consortium. Custom python, R, and/or bash scripts were then created in OpenPedCan using the primary harmonized output files.

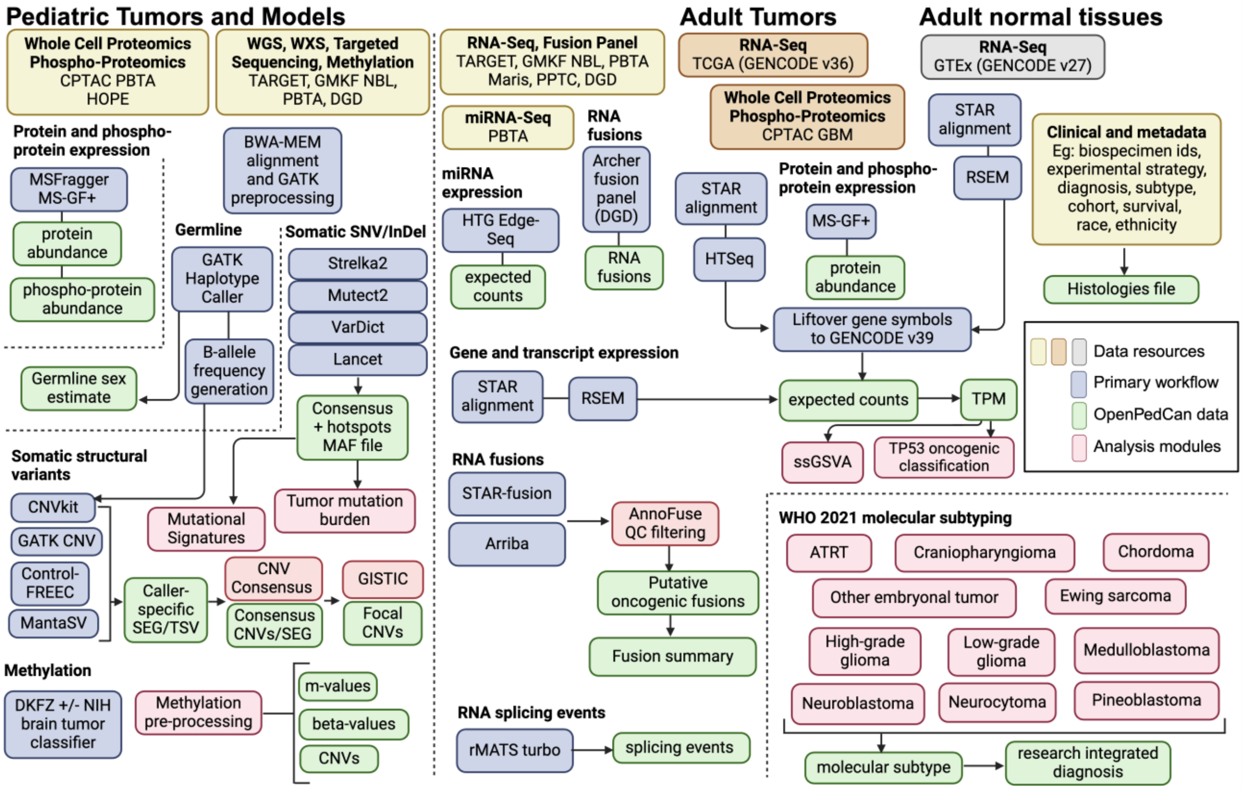

Figure 2: ***OpenPedCan Analysis Workflow.*** *Depicted are the datasets (yellow, orange, and grey) contained within OpenPedCan. These datasets are made available in a harmonized manner through primary analysis workflows (blue) for DNA, RNA, and/or proteogenomics data. Files derived from the primary analysis workflows (green) are released within OpenPedCan. Additional analysis modules developed within OpenPedCan (red) also generate results files (green) which are released within OpenPedCan.*

Method Details

Nucleic acids extraction and library preparation (PBTA X01 and miRNA-Seq)

For detailed methods about the OpenPBTA cohort, please refer to the manuscript [1]. For the PBTA X01 cohort, libraries were prepped using the Illumina TruSeq Strand-Specific Protocol to pull out poly-adenylated transcripts.

cDNA Library Construction

Total RNA was quantified using the Quant-iT RiboGreen RNA Assay Kit and normalized to 5ng/ul. Following plating, 2 uL of ERCC controls (using a 1:1000 dilution) were spiked into each sample. An aliquot of 325 ng for each sample was transferred into library preparation. The resultant 400bp cDNA went through dual-indexed library preparation: ‘A’ base addition, adapter ligation using P7 adapters, and PCR enrichment using P5 adapters. After enrichment, the libraries were quantified using Quant-iT PicoGreen (1:200 dilution). Samples were normalized to 5 ng/uL. The sample set was pooled and quantified using the KAPA Library Quantification Kit for Illumina Sequencing Platforms.

miRNA Extraction and Library Preparation

Total RNA for CBTN samples was extracted as described in OpenPBTA [1] and prepared according to the HTG Edge Seq protocol for the extracted RNA miRNA Whole transcriptome assay (WTA). 15ng of RNA were mixed in 25ul of lysis buffer, which were then loaded onto a 96-well plate. Human Fetal Brain Total RNA (Takara Bio USA, #636526) and Human Brain Total RNA (Ambion, Inc., Austin, TX, USA) were used as controls. The plate was loaded into the HTG EdgeSeq processor along with the miRNA WTA assay reagent pack. Samples were processed

for 18-20 hours, then were barcoded and amplified using a unique forward and reverse primer combination. PCR settings used for barcoding and amplification were 95C for 4 min, 16 cycles of (95C for 15 sec, 56C for 45 sec, 68C for 45 sec), and 68C for 10 min. Barcoded and amplified samples were cleaned using AMPure magnetic beads (Ampure XP,Cat# A63881). Libraries were quantified using the KAPA Biosystem assay qPCR kit (Kapa Biosystems Cat#KK4824) and CT values were used to determine the pM concentration of each library.

Data generation

PBTA X01 Illumina Sequencing Pooled libraries were normalized to 2nM and denatured using 0.1 N NaOH prior to sequencing. Flowcell cluster amplification and sequencing were performed according to the manufacturer’s protocols using the NovaSeq 6000. Each run was a 151bp paired-end with an eight-base index barcode read. Data was analyzed using the Broad Picard Pipeline which includes de-multiplexing and data aggregation.

PBTA miRNA Sequencing Libraries were pooled, denatured, and loaded onto sequencing cartridge. Libraries were sequenced using an Illumina Nextseq 500 per manufacturer guidelines. FASTQ files were generated from raw sequencing data using Illumina BaseSpace and analyzed with the HTG EdgeSeq Parser software v5.4.0.7543 to generate an excel file containing quantification of 2083 miRNAs per sample. Any sample that did not pass the quality control set by the HTG REVEAL software version 2.0.1 (Tuscon, AR, USA) was excluded from the analysis.

DNA WGS Alignment and SNP Calling

Please refer to the OpenPBTA manuscript for details on DNA WGS Alignment, prediction of participants’ genetic sex, and SNP calling for B-allele Frequency (BAF) generation. [1].

Somatic Mutation and INDEL Calling

For matched tumor/normal samples, we used the same mutation calling methods as described in OpenPBTA manuscript for details [1]. For tumor only samples, we ran Mutect2 from GATK v4.2.2.0 using the following workflow.

VCF annotation and MAF creation

Somatic variants were annotated by the Ensembl Variant Effect Predictor (VEP v105) [6]. From tumor only variant calls, we removed variants with `alt_depth == 0` or `t_depth < 4`.

Consensus SNV Calling (tumor/normal only)

We adopted the consensus SNV calling method described in OpenPBTA manuscript with adjustment [1]. For SNV calling, we combined four consensus SNV calling algorithms: Strelka2[7], Mutect2[8], Lancet[9], and VarDict[10].

Strelka2 outputs multi-nucleotide polymorphisms (MNPs) as consecutive single-nucleotide polymorphisms. In order preserve MNPs, we gather MNP calls from the other caller inputs, and search for evidence supporting these consecutive SNP calls as MNP candidates. Once found, the Strelka2 SNP calls supporting a MNP are converted to a single MNP call. This is done to preserve the predicted gene model as accurately as possible in our consensus calls. Consensus SNV from all four callers were collected and by default, calls that were detected in at least two calling algorithms or marked with “HotSpotAllele” were retained.

For all SNVs, potential non-hotspot germline variants were removed if they had a normal depth <= 7 and gnomAD allele frequency > 0.001. Final results were saved in MAF format.

Somatic Copy Number Variant (CNV) Calling

We called copy number variants for tumor/normal samples using Control-FREEC [11,12] and CNVkit [13] as described in the OpenPBTA manuscript [1]. We used GATK [14] to call CNVs for matched tumor/normal WGS samples when there were at least 30 male and 30 female normals from the same sequencing platform available for panel of normal creation. For tumor only samples, we used Control-FREEC with the following modifications. Instead of the b-allele frequency germline input file, we used the `dbSNP_v153_ucsc-compatible.converted.vt.decomp.norm.common_snps.vcf.gz` dbSNP common snps file and to avoid hard-to-call regions, utilized the `hg38_canonical_150.mappability` mappability file. Both are also linked in the public Kids First references CAVATICA project. The Control-FREEC tumor only workflow can be found here.

Somatic Structural Variant Calling (WGS samples only)

Please refer to the OpenPBTA manuscript for details [1].

Methylation Analysis

Methylation array preprocessing

We preprocessed raw Illumina 450K and EPIC 850K Infinium Human Methylation Bead Array intensities using the array preprocessing methods implemented in the `minfi Bioconductor package` [15]. We utilized either `preprocessFunnorm` when an array dataset had both tumor and normal samples or multiple OpenPedcan-defined `cancer_groups` and `preprocessQuantile` when an array dataset had only tumor samples from a single OpenPedcan-defined `cancer_group` to estimate usable methylation measurements (`beta-values` and `m-values`) and copy number (`cn-values`). Some Illumina Infinium array probes

targeting CpG loci contain single-nucleotide polymorphisms (SNPs) near or within the probe [16], which could affect DNA methylation measurements [17]. As the minfi preprocessing workflow recommends, we dropped probes containing common SNPs in dbSNP (minor allele frequency > 1%) at the CpG interrogation or the single nucleotide extensions.

Details of methylation array preprocessing are available in the OpenPedCan methylation-preprocessing module.

Methylation classification of brain tumor molecular subtypes

The Clinical Methylation Unit Laboratory of Pathology at the National Cancer Institute Center for Cancer Research ran the DKFZ brain classifier version 12.6, a comprehensive DNA methylation-based classification of CNS tumors across all entities and age groups [18] and/or the Bethesda Brain tumor classifier v2.0 (NIH_v2) and the combo reporter pipeline v2.0 on docker container trust1/bethesda:latest. Unprocessed IDAT-files from the Children's Brain Tumor Network (CBTN) `Infinium Human Methylation EPIC (850k) BeadChip arrays` were used as input and the following information was compiled into the `histologies.tsv` file: `dkfz_v12_methylation_subclass` (predicted methylation subtype), `dkfz_v12_methylation_subclass_score` (classification score), `dkfz_v12_methylation_mgmt_status` (*MGMT* methylation status), `dkfz_v12_methylation_mgmt_estimated` (estimated *MGMT* methylation fraction), `NIH_v2_methylation_Superfamily`, `NIH_v2_methylation_Superfamily_mean_score`, `NIH_v2_methylation_Superfamily_Consistency_score`, `NIH_v2_methylation_Class`, `NIH_v2_methylation_Class_mean_score`, `NIH_v2_methylation_Class_consistency_score`, `NIH_v2_methylation_Superfamily_match`, and `NIH_v2_methylation_Class_match`.

Gene Expression

The `tumor-normal-differential-expression` module performs differential expression analyses for all sets of Disease (`cancer_group`) and Dataset (`cohort`) across all genes found in the `gene-expression-rsem-tpm-collapsed.rds` table. The purpose of this analysis is to highlight the correlation and understand the variability in gene expression in different cancer conditions across different histological tissues. For OpenPedCan v12 data release, this module performs expression analysis over 102 cancer groups across 52 histological tissues for all 54,346 genes found in the dataset. This analysis was performed on the Children's Hospital of Philadelphia HPC and was configured to use 96G of RAM per CPU, with one task (one iteration of expression analysis for each set of tissue and cancer group) per CPU (total 102x52=5304 CPUs) using the R/DESeq2 package. Please refer to script `run-tumor-normal-differential-expression.sh` in the module for additional details on Slurm processing configuration. The same analysis can also be performed on CAVATICA, but requires further optimization. The module describes the steps for CAVATICA set up, and scripts to publish an application on the portal. The required data files are also available publicly on CAVATICA under the Open Pediatric Cancer (OpenPedCan) Open Access. Refer to the module for detailed description and scripts.

Abundance Estimation

Among the data sources used for OpenPedCan, GTEx and TCGA used GENCODE v26 and v36, respectively. Therefore, the gene symbols had to be harmonized to GENCODE v39 for compatibility with the rest of the dataset. The liftover process was done via a custom script. The script first constructs an object detailing the gene symbol changes from the HGNC symbol database. Using the symbol-change object, the script updates any columns containing gene

symbols. This liftover process was used on GTEx RNA-Seq, TCGA RNA-Seq, DGD fusions, and DNA hotspot files.

Additionally, the gene expression matrices had some instances where multiple Ensembl gene identifiers mapped to the same gene symbol. This was dealt with by filtering the expression matrix to only genes with [FPKM/TPM] > 0 and then selecting the instance of the gene symbol with the maximum mean [FPKM/TPM/Expected_count] value across samples. This enabled many downstream modules that require RNA-seq data have gene symbols as unique gene identifiers. Refer to collapse-rnaseq module for scripts and details.

Gene fusion detection from RNA-Seq

Gene fusions were called using Arriba [19] and STAR-Fusion [20] as previously reported in OpenPBTA [1]. We updated the `annoFuseData` R package to liftover gene symbols to be concordant with VEP v. 105. Fusions are now filtered with annoFuse [21] upstream and released in `fusion-annoFuse.tsv.gz`.

Gene fusion detection from fusion panels (DGD only)

Clinical RNA fusion calls from the CHOP DGD fusion panel are included in the data release in the `fusion-dgd.tsv.gz` file.

Splicing quantification

To detect alternative splicing events, we utilized rMATS turbo (v. 4.1.0) with Ensembl/GENCODE v39 GFF annotations using the Kids First RNA-Seq workflow. We used `--variable-read-length` and `-t paired` options and applied an additional filter to include only splicing events with total junction read counts greater than 10.

CPTAC PBTA, CPTAC GBM, and HOPE proteogenomics

The following methods are the general proteomics approaches used for the CPTAC PBTA [4], CPTAC GBM [5], and HOPE (pre-publication, correspondence with Dr. Pei Wang) studies. For specific descriptions of sample preparation, mass spectrometry instrumentation and approaches, and data generation, processing, or analysis please refer to the relevant publications.

TMT-11 Labeling and Phosphopeptide Enrichment

Proteome and phosphoproteome analysis of brain cancer samples in the CPTAC PBTA (pediatric), CPTAC GBM (adult), and HOPE (adolescent and young adult, AYA) cohort studies were structured as TMT11-plex experiments. Tumor samples were digested with LysC and trypsin. Digested peptides were labeled with TMT11-plex reagent and prepared for phosphopeptide enrichment. For each dataset, a common reference sample was compiled from representative samples within the cohort. Phosphopeptides were enriched using Immobilized Metal Affinity Chromatography (IMAC) with Fe^{3+}-NTA-agarose bead kits.

Liquid Chromatography with Tandem Mass Spectrometry (LC-MS/MS) Analysis

To reduce sample complexity, peptide samples were separated by high pH reversed phase HPLC fractionation. For CPTAC PBTA a total of 96 fractions were consolidated into 12 final fractions for LC-MS/MS analysis. For CPTAC GBM and HOPE cohorts a total of 96 fractions were consolidated into 24 fractions. For CPTAC PBTA, global proteome mass spectrometry analyses were performed on an Orbitrap Fusion Tribrid Mass Spectrometer and phosphoproteome analyses were performed on an Orbitrap Fusion Lumos Tribrid Mass Spectrometer. For CPTAC GBM and HOPE studies, mass spectrometry analysis was performed using an Orbitrap Fusion Lumos Mass Spectrometer.

Protein Identification

The CPTAC PBTA spectra data were analyzed with MSFragger version 20190628 [22] searching against a CPTAC harmonized RefSeq-based sequence database containing 41,457 proteins mapped to the human reference genome (GRCh38/hg38) obtained via the UCSC Table Browser on June 29, 2018, with the addition of 13 proteins encoded in the human mitochondrial genome, 264 common laboratory contaminant proteins, and an equal number of decoy sequences. The CPTAC GBM and HOPE spectra data were analyzed with MS-GF+ v9881 [23,24,25] searching against the RefSeq human protein sequence database downloaded on June 29, 2018 (hg38; 41,734 proteins), combined with 264 contaminants, and a decoy database composed of the forward and reversed protein sequences.

Protein Quantification and Data Analysis

Relative protein (gene) abundance was calculated as the ratio of sample abundance to reference abundance using the summed reporter ion intensities from peptides mapped to the respective gene. For phosphoproteomic datasets, data were not summarized by protein but left at the phosphopeptide level. Global normalization was performed on the gene-level abundance matrix (log2 ratio) for global proteomic and on the site-level abundance matrix (log2 ratio) for phosphoproteomic data. The median, log2 relative protein or peptide abundance for each sample was calculated and used to normalize each sample to achieve a common median of 0. To identify TMT outliers, inter-TMT t-tests were performed for each individual protein or phosphopeptide. Batch effects were checked using the log2 relative protein or phosphopeptide abundance and corrected using the Combat algorithm [26]. Imputation was performed after batch effect correction for proteins or phosphopeptides with a missing rate < 50%. For the phosphopeptide datasets, 440 markers associated with cold-regulated ischemia genes were filtered and removed.

Creation of OpenPedCan Analysis modules

Gene Set Variation Analysis (`gene-set-enrichment-analysis` analysis module)

Please refer to the OpenPBTA manuscript for details [1].

Fusion prioritization (`fusion_filtering` analysis module)

The `fusion_filtering` module filters artifacts and annotates fusion calls, with prioritization for oncogenic fusions, for the fusion calls from STAR-Fusion and Arriba. After artifact filtering, fusions were prioritized and annotated as "putative oncogenic fusions" when at least one gene was a known kinase, oncogene, tumor suppressor, curated transcription factor, on the COSMIC Cancer Gene Census List, or observed in TCGA. Fusions were retained in this module if they were called by both callers, recurrent or specific to a cancer group, or annotated as a putative oncogenic fusion. Please refer to the module linked above for more detailed documentation and scripts.

Consensus CNV Calling (WGS samples only) (`copy_number_consensus_call*` analysis modules)

We adopted the consensus CNV calling described in OpenPBTA manuscript [1] with minor adjustments. For each caller and sample with WGS performed, we called CNVs based on consensus among Control-FREEC [11,12], CNVkit [13], and GATK [14]. Sample and consensus caller files with more than 2,500 CNVs were removed to de-noise and increase data quality, based on cutoffs used in GISTIC [27]. For each sample, we included the following regions in the final consensus set: 1) regions with reciprocal overlap of 50% or more between at least two of

the callers; 2) smaller CNV regions in which more than 90% of regions were covered by another caller. For GATK, if a panel of normal was not able to be created (required 30 male and 30 female with the same sequencing platform), consensus was run for that tumor using Control-FREEC, CNVkit, and MantaSV. We defined copy number as NA for any regions that had a neutral call for the samples included in the consensus file. We merged CNV regions within 10,000 bp of each other with the same direction of gain or loss into single region.

Any CNVs that overlapped 50% or more with immunoglobulin, telomeric, centromeric, segment duplicated regions, or that were shorter than 3000 bp were filtered out. The CNVKit calls for WXS samples were appended to the consensus CNV file.

Focal Copy Number Calling (`focal-cn-file-preparation` analysis module)

Please refer to the OpenPBTA manuscript for details on assignment of copy number status values to CNV segments, cytobands, and genes [1]. We applied criteria to resolve instances of multiple conflicting status calls for the same gene and sample, which are described in detail in the focal-cn-file-preparation module. Briefly, we prioritized 1) non-neutral status calls, 2) calls made from dominant segments with respect to gene overlap, and 3) amplification and deep deletion status calls over gain and loss calls, respectively, when selecting a dominant status call per gene and sample. These methods resolved >99% of duplicated gene-level status calls.

Mutational Signatures (`mutational-signatures` analysis module)

We obtained mutational signature weights (i.e., exposures) from consensus SNVs using the deconstructSigs R package [28]. We estimated weights for single- and double-base substitution (SBS and DBS, respectively) signatures from the Catalogue of Somatic Mutations in Cancer (COSMIC) database versions 2 and 3.3, as well as SBS signatures from Alexandrov et al. 2013 [29]. The following COSMIC SBS signatures were excluded from weight estimation in all tumors:

1) sequencing artifact signatures, 2) signatures associated with environmental exposure, and 3) signatures with an unknown etiology. Additionally, we excluded therapy-associated signatures from mutational signature weight estimation in tumors collected prior to treatment (i.e. “Initial CNS Tumor” or “Primary Tumor”).

Tumor Mutation Burden [TMB] (`tmb-calculation` analysis module)

Recent clinical studies have associated high TMB with improved patient response rates and survival benefit from immune checkpoint inhibitors [30].

The Tumor Mutation Burden (TMB) `tmb-calculation` module was adapted from the `snv-callers` module of the OpenPBTA project [1]. Here, we use mutations in the `snv-consensus-plus-hotspots.maf.tsv.gz` file which is generated using Kids First DRC Consensus Calling Workflow and is included in the OpenPedCan data download. The consensus MAF contains SNVs or MNVs called in at least 2 of the 4 callers (Mutect2, Strelka2, Lancet, and Vardict) plus hotspot mutations if called in 1 of the 4 callers. We calculated TMB for tumor samples sequenced with either WGS or WXS. Briefly, we split the SNV consensus MAF into SNVs and multinucleotide variants (MNVs). We split the MNV subset into SNV calls, merged those back with the SNVs subset, and then removed sample-specific redundant calls. The resulting merged and non-redundant SNV consensus calls were used as input for the TMB calculation. We tallied only nonsynonymous variants with classifications of high/moderate consequence (“Missense_Mutation”, “Frame_Shift_Del”, “In_Frame_Ins”, “Frame_Shift_Ins”, “Splice_Site”, “Nonsense_Mutation”, “In_Frame_Del”, “Nonstop_Mutation”, and “Translation_Start_Site”) for the numerator. All BED files are provided in the data release.

All mutation TMB

For WGS samples, we calculated the size of the genome covered as the intersection of Strelka2 and Mutect2's effectively surveyed areas, regions common to all variant callers, and used this as the denominator. `WGS_all_mutations_TMB = (total # mutations in consensus MAF) / intersection_strelka_mutect_vardict_genome_size` For WXS samples, we used the size of the WXS bed region file as the denominator. `WXS_all_mutations_TMB = (total # mutations in consensus MAF)) / wxs_genome_size`

Coding only TMB

We generated coding only TMB from the consensus MAF as well. We calculated the intersection for Strelka2 and Mutect2 surveyed regions using the coding sequence ranges in the GENCODE v39 gtf supplied in the OpenPedCan data download. We removed SNVs outside of these coding sequences prior to implementing the TMB calculation below: `WGS_coding_only_TMB = (total # coding mutations in consensus MAF) / intersection_wgs_strelka_mutect_vardict_CDS_genome_size` For WXS samples, we intersected each WXS bed region file with the GENCODE v39 coding sequence, sum only variants within this region for the numerator, and calculate the size of this region as the denominator. `WXS_coding_only_TMB = (total # coding mutations in consensus MAF) / intersection_wxs_CDS_genome_size`

Finally, we include an option (`nonsynfilter_focr`) to use specific nonsynonymous mutation variant classifications recommended from the TMB Harmonization Project.

Molecular Subtyping

Here, we build upon the molecular subtyping performed in OpenPBTA [1] to align with WHO 2021 subtypes [31]. Molecular subtypes were generated per tumor event and are listed for each

biospecimen in **Supplemental Table 1**, with the number of tumors grouped by broad histology and molecular subtype in **Supplemental Table 2**.

High-grade gliomas

High-grade gliomas (HGG) were categorized based on a combination of clinical information, molecular features, and DNA methylation data. H3 K28-altered diffuse midline gliomas (DMG) were classified based on the presence of a p.K28M or p.K28I mutation in *H3F3A*, *HIST1H3B*, *HIST1H3C*, or *HIST2H3C*, or a high-confidence DKFZ methylation score (>=0.8) in the appropriate subclass. Oligodendroglioma, IDH-mutant tumors were classified based on high-confidence "O_IDH" methylation classifications, and oligosarcoma, IDH-mutant tumors were defined as those with high-confidence "OLIGOSARC_IDH" methylation classifications. Pleomorphic xanthoastrocytomas (PXA) were classified using the following criteria: 1) methylation subtype is high-confidence "PXA" or `pathology_free_text_diagnosis` contains "pleomorphic xanthoastrocytoma" or "pxa", and 2) tumor contains a BRAF V600E mutation and a *CDKN2A* or *CDKN2B* homozygous deletion. Methylation classifications were used in classifying the following subtypes:

1. DHG, H3 G35 ("DHG_G34" and "GBM_G34" classifications)
2. HGG, IDH ("A_IDH_HG" and "GBM_IDH" classifications)
3. HGG, H3 wild type (methylation classification contains "GBM_MES", "GBM_RTK", "HGG_", "HGAP", "AAP", or "ped_")

A new high-grade glioma entity called infant-type hemispheric gliomas (IHGs), characterized by distinct gene fusions enriched in receptor tyrosine kinase (RTK) genes including *ALK*, *NTRK1/2/3*, *ROS1* or *MET*, was identified in 2021 [32]. To identify IHG tumors, first, tumors which were classified as "IHG" by the DKFZ methylation classifier or diagnosed as "infant type

hemispheric glioma" from `pathology_free_text_diagnosis` were selected [18]. Then, the corresponding tumor RNA-seq data were utilized to seek the evidence for RTK gene fusion. Based on the specific RTK gene fusion present in the samples, IHGs were further classified as "IHG, ALK-altered", "IHG, NTRK-altered", "IHG, ROS1-altered", or "IHG, MET-altered". If no fusion was observed, the samples were identified as "IHG, To be classified".

Atypical teratoid rhabdoid tumors

Atypical teratoid rhabdoid tumors (ATRT) tumors were categorized into three subtypes: "ATRT, MYC", "ATRT, SHH", and "ATRT, TYR" [33]. In OpenPedCan, the molecular subtyping of ATRT was based solely on the DNA methylation data. Briefly, ATRT samples with a high confidence DKFZ methylation subclass score (>= 0.8) were selected and subtypes were assigned based on the DKFZ methylation subclass [18]. Samples with low confidence DKFZ methylation subclass scores (< 0.8) were identified as "ATRT, To be classified".

Neuroblastoma tumors

Neuroblastoma (NBL) tumors with a pathology diagnosis of neuroblastoma, ganglioneuroblastoma, or ganglioneuroma were subtyped based on their MYCN copy number status as either "NBL, MYCN amplified" or "NBL, MYCN non-amplified". If `pathology_free_text_diagnosis` was "NBL, MYCN non-amplified" and the genetic data suggested MYCN amplification, the samples were subtyped as "NBL, MYCN amplified". On the other hand, if `pathology_free_text_diagnosis` was "NBL, MYCN amplified" and the genetic data suggested MYCN non-amplification, the RNA-Seq gene expression level of *MYCN* was used as a prediction indicator. In those cases, samples with *MYCN* gene expression above or below the cutoff (TPM >= 140.83 based on visual inspection of MYCN CNV status) were subtyped as "NBL, MYCN amplified" and "NBL, MYCN non-amplified", respectively. *MYCN* gene

expression was also used to subtype samples without DNA sequencing data. If a sample did not fit none of these situations, it was denoted as "NBL, To be classified".

Craniopharyngiomas

In addition to molecular criteria established in OpenPBTA [1], craniopharyngiomas (CRANIO) are now subtyped using DNA methylation classifiers. Craniopharyngiomas with a high-confidence methylation subclass containing "CPH_PAP" were classified as papillary (CRANIO, PAP), and those with high-confidence methylation subclass containing "CPH_ADM" were classified as adamantinomatous (CRANIO, ADAM), respectively.

Ependymomas

Ependymomas (EPN) are subtyped using the following criteria:

1. Any spinal tumor with *MYCN* amplification or with a high-confidence "EPN, SP-MYCN" methylation classification was subtyped as EPN, spinal and MYCN-amplified (SP-MYCN).
2. EPN tumors containing one or more gene fusions of *YAP1::MAMLD1*, *YAP1::MAML2*, or *YAP1::FAM118B*, or else had a high-confidence "EPN, ST YAP1" methylation classification were subtyped as EPN, ST YAP1.
3. EPN tumors containing one or more gene fusions of *ZFTA::RELA* or *ZFTA::MAML2*, or else had a high-confidence "EPN, ST ZFTA" methylation classification were subtyped as EPN, ST ZFTA. This reflects an update to WHO classifications that now characterizes this subtype based on *ZFTA* fusions rather than *RELA* fusions.
4. EPN tumors with 1) chromosome 1q gain and *TKTL1* over-expression, or 2) *EZHIP* over-expression, or 3) posterior fossa anatomical location and a histone H3 K28 mutation in *H3F3A*, *HIST1H3B*, *HIST1H3C*, or *HIST2H3C*, or 4) a high-confidence "EPN, PF A"

methylation classification were subtyped as posterior fossa group A ependymomas (EPN, PF A).

5. Tumors with 1) chr 6p or 6q loss and *GPBP1* or *IFT46* over-expression, or 2) a high-confidence "EPN, PF B" methylation classification were subtyped as posterior fossa group B ependymomas (EPN, PF B).
6. EPN tumors with a high-confidence "EPN, MPE" methylation classification were subtyped as myxopapillary ependymomas (EPN, MPE).
7. EPN tumors with a high-confidence "EPN, PF SE" methylation classification were subtyped as posterior fossa subependymomas (EPN, PF SE).
8. EPN tumors with a high-confidence "EPN, SP SE" methylation classification were subtyped as spinal subependymomas (EPN, SP SE).
9. EPN tumors with a high-confidence "EPN, SP" methylation classification were subtyped as spinal ependymomas (EPN, SP).
10. All other EPN tumors were classified as "EPN, To be classified".

Low-grade gliomas

In addition to subtyping methods described in OpenPBTA [1], high-confidence methylation classifications are now used in classifying the following low-grade glioma (LGG) subtypes:

1. LGG, other MAPK-altered (methylation subclass "PA_MID" or "PLNTY")
2. LGG, FGFR-altered (methylation subclass "PA_INF_FGFR")
3. LGG, IDH-altered (methylation subclass "A_IDH_LG")
4. LGG, MYB/MYBL1 fusion (methylation subclass "AG_MYB" or "LGG_MYB")
5. LGG, MAPK-altered (methylation subclass "LGG, MAPK")
6. LGG, BRAF- and MAPK-altered (methylation subclass "LGG, BRAF/MAPK")

7. SEGA, to be classified (methylation subclass “SEGA, To be classified”)

Medulloblastomas (MBs) In addition to our previous work classifying MB tumors into the four major subtypes (WNT, SHH, Group 3, and Group 4) using the transcriptomic MedulloClassifier [34], we integrated high-confidence methylation classification, demographic, and molecular criteria to molecularly subtype SHH tumors into one of four subgroups (alpha, beta, gamma, or delta) (**Figure 3**).

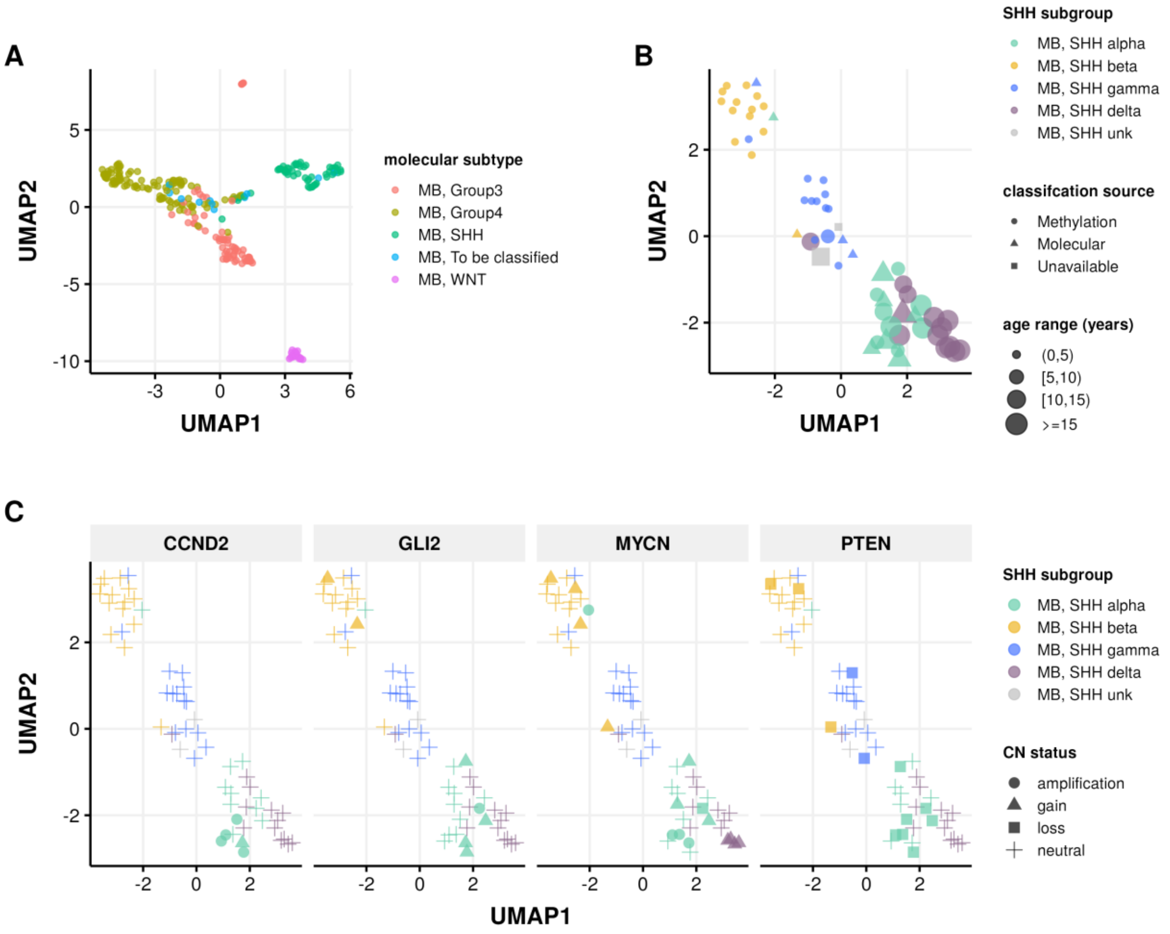

Figure 3: ***Medulloblastoma Sample Clustering.*** *A, UMAP projection of 271 MB tumors and B, 63 SHH-activated MB tumors using methylation beta values of the 20,000 most variable probes from the Infinium MethylationEPIC array. C, UMAP projection of MB, SHH activated samples*

indicating copy number status of SHH subgroup known somatic driver genes CCND2, GLI2, MYCN, and PTEN.

We implemented molecular subtyping as follows:

1. MB tumors with methylation classification that contains “MB_SHH” are subtyped as SHH-activated medulloblastoma (MB, SHH)
2. MB tumors with “MB_G34_I”, “MB_G34_II”, “MB_G34_III”, and “MB_G334_IV” methylation classifications are subtyped as medulloblastoma group 3 (MB, Group3)
3. MB tumors with “MB_G34_V”, “MB_G34_VI”, “MB_G34_VII”, and “MB_G334_VIII” methylation classifications are subtyped as medulloblastoma group 4 (MB, Group4)
4. MB tumors with “MB_WNT” methylation classification are subtyped as WNT-activated MB (MB, WNT)
5. MB tumors with “MB_MYO” methylation classification are subtyped as medulloblastomas with myogenic differentiation (MB, MYO)

We classified MB, SHH subtype tumors using the following criteria:

1. *MB, SHH alpha*: sample has a high-confidence “MB_SHH_3” methylation classification, or patient had an age at diagnosis >= 2 years and harbored one of the following molecular alterations in tumor or germline:
    - *MYCN*, *GLI2*, or *CCND2* amplification or sample TPM z-score >= 2 in tumor.
    - A pathogenic or likely pathogenic germline variant in *ELP1* or *TP53*.
    - A *TP53* hotspot mutation in tumor.
    - Chromosome 9p gain or chromosome 17p loss in tumor.

2. *MB, SHH beta*: sample has a high-confidence “MB_SHH_1” methylation classification, or patient had an age at diagnosis < 5 years and harbored one of the following molecular alterations:

   - A *KMT2D* loss of function variant.
   - *PTEN* copy number loss or deep deletion, or sample TPM z-score < -2.
   - Chromosome 2p or 2q gain.

3. *MB, SHH gamma*: sample has a high-confidence “MB_SHH_2” methylation classification, or patient had an age at diagnosis < 5 years and tumor harbored a chromosome 2p arm gain.

4. *MB, SHH delta*: sample has a high-confidence “MB_SHH_4” methylation classification, or patient had an age at diagnosis >= 10 years and harbored one of the following molecular alterations in tumor:

   - a *DDX3X* or *SMO* loss-of-function mutation.
   - a hotspot *TERT* or U1 snRNA gene mutation.
   - Chromosome 14q arm loss.

Pineoblastomas

Pineoblastomas (PB) are classified as follows using high-confidence methylation classifications:

1. Pineoblastoma, MYC/FOXR2-activated (“PB_FOXR2” methylation classification)
2. Pineoblastoma, RB1-altered (“PB_RB1” methylation classification)
3. Pineoblastoma, group 1 (“PB_GRP1A” and “PB_GRP1B” methylation classifications)
4. Pineoblastoma, group 2 (“PB_GRP2” methylation classification)
5. All other pineoblastomas were classified as “PB, To be classified”

non-MB, non-ATRT Embryonal Tumors

Updates were made to non-MB, non-ATRT embryonal tumor subtyping as follows:

1. Embryonal tumors with multilayered rosettes and C19MC-altered (ETMR, C19MC-altered) were classified based on 1) high-confidence “ETMR_C19MC” methylation classification or 2) *TTYH1* gene fusion and either chromosome 19 amplification or *LIN28A* over-expression.
2. ETMR, not otherwise specified (NOS) were classified based on *LIN28A* over-expression and no *TTYH1* gene fusion.

TP53 Alteration Annotation (`tp53_nf1_score` analysis module)

Please refer to the OpenPBTA manuscript for details [1].

Selection of independent samples (`independent-samples` analysis module)

For analyses that require all input biospecimens to be independent, we use the OpenPedCan-analysis independent-samples module to select only one biospecimen from each input participant. For each input participant of an analysis, the independent biospecimen is selected based on the analysis-specific filters and preferences for the biospecimen metadata, such as experimental strategy, cancer group, and tumor descriptor.

Data Validation and Quality Control

We ran NGSCheckMate [35] to confirm tumor/normal sample matches as described in the OpenPBTA manuscript [1] and excluded mismatched samples. We also ran `somalier relate` [36] to identify potential mismatched samples. We required that at least 20M total reads with 50% of RNA-Seq reads mapped to the human reference for samples to be included in

analysis. We required at least 20X coverage for tumor DNA samples to be included in this analysis. Additional DNA- and RNA-sequencing quality control metrics can be found in **Supplemental Table 4**.

Re-use potential

OpenPedCan serves as a community resource whose outputs and/or code can be leveraged directly to ask research questions or serve as an orthogonal validation dataset. We encourage re-use of the data, ideas and suggestions for improving the data or adding analyses, and/or direct code contributions through a pull-request. Further, the analysis modules can be run within the project Docker container locally or on EC2 and scaled as the data size increases.

Availability of source code and requirements

Project name: The Open Pediatric Cancer (OpenPedCan) Project

Project home page: https://github.com/d3b-center/OpenPedCan-analysis

Operating system(s): Platform independent

Programming languages: R, Python, bash

Other requirements: CAVATICA, Docker image at pgc-images.sbgenomics.com/d3b-bixu/openpedcanverse:latest

License: CC-BY 4.0

Primary analyses were performed using Gabriella Miller Kids First pipelines and are listed in the methods section. Analysis modules were developed within https://github.com/AlexsLemonade/OpenPBTA-analysis [1], modified based on OpenPBTA, or

newly created and can be found within the https://github.com/d3b-center/OpenPedCan-analysis publicly available repository.

Software versions are documented in **Supplemental Table 3**.

Data Availability

Datasets

The datasets supporting this study are available as follows: The TARGET dataset is available in dbGAP under phs000218.v23.p8 [37]. The GMKF Neuroblastoma dataset is available in dbGAP under phs001436.v1.p1[38]. The Pediatric Brain Tumor Atlas data (PBTA), containing the subcohorts OpenPBTA, Kids First PBTA (X01), Chordoma Foundation, MI-ONCOSEQ Study, PNOC, and DGD is available in dbGAP under phs002517.v4.p2 [39] or in the Kids First Portal (kidsfirstdrc.org). The raw Genotype-Tissue Expression (GTEx) dataset is available in dbGAP under phs000424.v9.p2 and publicly available at https://gtexportal.org/home/. The Cancer Genome Atlas (TCGA) dataset is available in dbGAP under phs000178.v11.p8 [40].

Merged summary files for the latest release of OpenPedCan are openly accessible in CAVATICA or via `download-data.sh` script in the https://github.com/d3b-center/OpenPedCan-analysis repository. Cancer group summary data from release v11 are visible within the NCI's pediatric Molecular Targets Platform. Cohort, cancer group, and individual data are visible within PedcBioPortal

Acknowledgments

We are incredibly grateful to each patient and family for donating tissue and associated metadata and clinical data to their respective consortia. This project has been funded in whole

or in part with Federal funds from the National Cancer Institute, National Institutes of Health, under Contract No. 75N91019D00024, Task Order No. 75N91020F00003 (DMT, JLR, SJD, JMM, ST, AF, ACR). The content of this publication does not necessarily reflect the views or policies of the Department of Health and Human Services, nor does mention of trade names, commercial products or organizations imply endorsement by the U.S. Government. We thank Rocky Breslow for GitHub actions contributions and Rust Turakulov for contributing to methylation data analysis.

Author Contributions

**Author**	**Contributions**
Zhuangzhuang Geng	Data curation, Formal analysis, Investigation, Methodology, Software, Writing – Original draft
Eric Wafula	Formal analysis, Software, Investigation, Writing – Original draft
Ryan J. Corbett	Formal analysis, Writing - original draft
Yuanchao Zhang	Software, Formal analysis, Methodology, Writing – Original draft
Run Jin	Formal analysis
Krutika S. Gaonkar	Data curation, Formal analysis, Investigation
Sangeeta Shukla	Formal analysis, Investigation, Methodology, Writing – Original draft, Writing - Review and editing
Komal S. Rathi	Formal analysis, Investigation, Methodology
Dave Hill	Formal analysis, Writing - original draft
Aditya Lahiri	Formal analysis, Investigation, Methodology, Writing – Original draft
Daniel P. Miller	Formal analysis, Writing – Original draft

Author	Contributions
Alex Sickler	Methodology, Formal analysis
Kelsey Keith	Writing - original draft, Formal Analysis
Christopher Blackden	Software
Antonia Chroni	Validation
Miguel A. Brown	Data curation, Methodology, Formal analysis, Investigation, Software, Supervision, Writing – Original draft
Adam A. Kraya	Methodology
Brian R. Rood	Data Curation
Adam C. Resnick	Funding acquisition, Resources
Nicholas Van Kuren	Data curation, Software
John M. Maris	Funding acquisition
Alvin Farrel	Supervision, Investigation, Methodology, Funding acquisition
Mateusz P. Koptyra	Data curation, Investigation, Methodology, Supervision
Gerri R. Trooskin	Funding acquisition
Noel Coleman	Data curation
Yuankun Zhu	Supervision
Stephanie Stefankiewicz	Project administration
Zied Abdullaev	Formal Analysis, Investigation, Data curation
Asif T Chinwalla	Project administration, Supervision, Methodology, Investigation, Validation

**Author**	**Contributions**
Mariarita Santi	Investigation, Validation
Ammar S. Naqvi	Methodology, Writing – Original draft
Jennifer L. Mason	Supervision
Carl J. Koschmann	Data Curation
Xiaoyan Huang	Formal analysis, Software
Sharon J. Diskin	Funding acquisition
Kenneth Aldape	Formal Analysis, Investigation, Data curation
Bailey K. Farrow	Data curation, Software, Project administration, Supervision
Weiping Ma	Formal Analysis, Investigation, Data curation
Bo Zhang	Data curation, Formal analysis
Brian M. Ennis	Formal analysis
Sarah Tasian	Funding acquisition
Saksham Phul	Formal analysis
Matthew R. Lueder	Data curation
Chuwei Zhong	Formal analysis
Joseph M. Dybas	Writing – Original draft, Methodology
Pei Wang	Formal Analysis, Supervision
Deanne Taylor	Conceptualization, Data curation, Funding acquisition, Investigation, Methodology, Supervision, Project administration
Jo Lynne Rokita	Conceptualization, Data curation, Formal analysis, Funding acquisition, Project administration, Investigation, Methodology, Software, Supervision, Writing – Original

Author	Contributions
	draft, Writing - Review and editing

Declarations of Interest

The authors declare no conflicts.

Supplemental Information Titles and Legends

Supplemental Table 1 README, metadata, and clinical data for each patient and biospecimen in OpenPedCan.

Supplemental Table 2 Number of tumors and corresponding patients from which WHO 2021 molecular subtypes were generated through OpenPedCan analysis modules are listed in Sheet 1. Molecular subgroups (alpha, beta, gamma, or delta) for medulloblastoma SHH tumors are listed in Sheet 2.

Supplemental Table 3 Listed are the software versions for all packages and workflows used in this manuscript.

Supplemental Table 4 Read count and coverage for DNA- and RNA-sequencing biospecimens.

References

1. **OpenPBTA: The Open Pediatric Brain Tumor Atlas** Joshua A Shapiro, Krutika S Gaonkar, Stephanie J Spielman, Candace L Savonen, Chante J Bethell, Run Jin, Komal S Rathi, Yuankun Zhu, Laura E Egolf, Bailey K Farrow, … Jaclyn N Taroni *Cell Genomics* (2023-

07) https://doi.org/gr92p6 DOI: 10.1016/j.xgen.2023.100340 · PMID: 37492101 · PMCID: PMC10363844

2. **Genomic Profiling of Childhood Tumor Patient-Derived Xenograft Models to Enable Rational Clinical Trial Design** Jo Lynne Rokita, Komal S Rathi, Maria F Cardenas, Kristen A Upton, Joy Jayaseelan, Katherine L Cross, Jacob Pfeil, Laura E Egolf, Gregory P Way, Alvin Farrel, … John M Maris *Cell Reports* (2019-11) https://doi.org/gg596n DOI: 10.1016/j.celrep.2019.09.071 · PMID: 31693904 · PMCID: PMC6880934

3. **Michigan Center for Translational Pathology** https://mctp.med.umich.edu

4. **Integrated Proteogenomic Characterization across Major Histological Types of Pediatric Brain Cancer** Francesca Petralia, Nicole Tignor, Boris Reva, Mateusz Koptyra, Shrabanti Chowdhury, Dmitry Rykunov, Azra Krek, Weiping Ma, Yuankun Zhu, Jiayi Ji, … William E Bocik *Cell* (2020-12) https://doi.org/ghqjkz DOI: 10.1016/j.cell.2020.10.044 · PMID: 33242424 · PMCID: PMC8143193

5. **Proteogenomic and metabolomic characterization of human glioblastoma** Liang-Bo Wang, Alla Karpova, Marina A Gritsenko, Jennifer E Kyle, Song Cao, Yize Li, Dmitry Rykunov, Antonio Colaprico, Joseph H Rothstein, Runyu Hong, … Jun Zhu *Cancer Cell* (2021-04) https://doi.org/gh7whf DOI: 10.1016/j.ccell.2021.01.006 · PMID: 33577785 · PMCID: PMC8044053

6. **The Ensembl Variant Effect Predictor** William McLaren, Laurent Gil, Sarah E Hunt, Harpreet Singh Riat, Graham RS Ritchie, Anja Thormann, Paul Flicek, Fiona Cunningham *Genome Biology* (2016-06-06) https://doi.org/gdz75c DOI: 10.1186/s13059-016-0974-4 · PMID: 27268795 · PMCID: PMC4893825

7. **Strelka2: fast and accurate calling of germline and somatic variants** Sangtae Kim, Konrad Scheffler, Aaron L Halpern, Mitchell A Bekritsky, Eunho Noh, Morten Källberg, Xiaoyu Chen, Yeonbin Kim, Doruk Beyter, Peter Krusche, Christopher T Saunders *Nature Methods* (2018-07-16) https://doi.org/gdwrp4 DOI: 10.1038/s41592-018-0051-x · PMID: 30013048

8. **Calling Somatic SNVs and Indels with Mutect2** David Benjamin, Takuto Sato, Kristian Cibulskis, Gad Getz, Chip Stewart, Lee Lichtenstein *Cold Spring Harbor Laboratory* (2019-12-02) https://doi.org/ggntwv DOI: 10.1101/861054

9. **Genome-wide somatic variant calling using localized colored de Bruijn graphs** Giuseppe Narzisi, André Corvelo, Kanika Arora, Ewa A Bergmann, Minita Shah, Rajeeva Musunuri, Anne-Katrin Emde, Nicolas Robine, Vladimir Vacic, Michael C Zody *Communications Biology* (2018-03-22) https://doi.org/gfcfr8 DOI: 10.1038/s42003-018-0023-9 · PMID: 30271907 · PMCID: PMC6123722

10. **VarDict: a novel and versatile variant caller for next-generation sequencing in cancer research** Zhongwu Lai, Aleksandra Markovets, Miika Ahdesmaki, Brad Chapman, Oliver Hofmann, Robert McEwen, Justin Johnson, Brian Dougherty, JCarl Barrett, Jonathan R Dry *Nucleic Acids Research* (2016-04-07) https://doi.org/f8v6qz DOI: 10.1093/nar/gkw227 · PMID: 27060149 · PMCID: PMC4914105

11. **Control-FREEC: a tool for assessing copy number and allelic content using next-generation sequencing data** Valentina Boeva, Tatiana Popova, Kevin Bleakley, Pierre Chiche, Julie Cappo, Gudrun Schleiermacher, Isabelle Janoueix-Lerosey, Olivier Delattre, Emmanuel Barillot *Bioinformatics* (2011-12-06) https://doi.org/ckt4vz DOI: 10.1093/bioinformatics/btr670 · PMID: 22155870 · PMCID: PMC3268243

12. **Control-free calling of copy number alterations in deep-sequencing data using GC-content normalization** Valentina Boeva, Andrei Zinovyev, Kevin Bleakley, Jean-Philippe Vert, Isabelle Janoueix-Lerosey, Olivier Delattre, Emmanuel Barillot *Bioinformatics* (2010-11-15) https://doi.org/c6bcps DOI: 10.1093/bioinformatics/btq635 · PMID: 21081509 · PMCID: PMC3018818

13. **CNVkit: Genome-Wide Copy Number Detection and Visualization from Targeted DNA Sequencing** Eric Talevich, AHunter Shain, Thomas Botton, Boris C Bastian *PLOS Computational Biology* (2016-04-21) https://doi.org/c9pd DOI: 10.1371/journal.pcbi.1004873 · PMID: 27100738 · PMCID: PMC4839673

14. **The Genome Analysis Toolkit: A MapReduce framework for analyzing next-generation DNA sequencing data** Aaron McKenna, Matthew Hanna, Eric Banks, Andrey Sivachenko, Kristian Cibulskis, Andrew Kernytsky, Kiran Garimella, David Altshuler, Stacey Gabriel, Mark Daly, Mark A DePristo *Genome Research* (2010-07-19) https://doi.org/bnzbn6 DOI: 10.1101/gr.107524.110 · PMID: 20644199 · PMCID: PMC2928508

15. **Preprocessing, normalization and integration of the Illumina HumanMethylationEPIC array with minfi** Jean-Philippe Fortin, Timothy J Triche Jr, Kasper D Hansen *Bioinformatics* (2016-11-29) https://doi.org/f9x7kd DOI: 10.1093/bioinformatics/btw691 · PMID: 28035024 · PMCID: PMC5408810

16. **Review of processing and analysis methods for DNA methylation array data** CS Wilhelm-Benartzi, DC Koestler, MR Karagas, JM Flanagan, BC Christensen, KT Kelsey, CJ Marsit, EA Houseman, R Brown *British Journal of Cancer* (2013-08-27) https://doi.org/gb9qvv DOI: 10.1038/bjc.2013.496 · PMID: 23982603 · PMCID: PMC3777004

17. **Impact of SNPs on methylation readouts by Illumina Infinium HumanMethylation450 BeadChip Array: implications for comparative population studies** Patrycja Daca-Roszak, Aleksandra Pfeifer, Jadwiga Żebracka-Gala, Dagmara Rusinek, Aleksandra Szybińska, Barbara Jarząb, Michał Witt, Ewa Ziętkiewicz *BMC Genomics* (2015-11-25) https://doi.org/gb3h5r DOI: 10.1186/s12864-015-2202-0 · PMID: 26607064 · PMCID: PMC4659175

18. **DNA methylation-based classification of central nervous system tumours** David Capper, David TW Jones, Martin Sill, Volker Hovestadt, Daniel Schrimpf, Dominik Sturm, Christian Koelsche, Felix Sahm, Lukas Chavez, David E Reuss, … Stefan M Pfister *Nature* (2018-03-14) https://doi.org/gc5t36 DOI: 10.1038/nature26000 · PMID: 29539639 · PMCID: PMC6093218

19. **Accurate and efficient detection of gene fusions from RNA sequencing data** Sebastian Uhrig, Julia Ellermann, Tatjana Walther, Pauline Burkhardt, Martina Fröhlich, Barbara Hutter, Umut H Toprak, Olaf Neumann, Albrecht Stenzinger, Claudia Scholl, … Benedikt Brors *Genome Research* (2021-01-13) https://doi.org/gjvdvp DOI: 10.1101/gr.257246.119 · PMID: 33441414 · PMCID: PMC7919457

20. **STAR-Fusion: Fast and Accurate Fusion Transcript Detection from RNA-Seq** Brian J Haas, Alex Dobin, Nicolas Stransky, Bo Li, Xiao Yang, Timothy Tickle, Asma Bankapur, Carrie Ganote, Thomas G Doak, Nathalie Pochet, … Aviv Regev *Cold Spring Harbor Laboratory* (2017-03-24) https://doi.org/gf5pc5 DOI: 10.1101/120295

21. **annoFuse: an R Package to annotate, prioritize, and interactively explore putative oncogenic RNA fusions** Krutika S Gaonkar, Federico Marini, Komal S Rathi, Payal Jain, Yuankun Zhu, Nicholas A Chimicles, Miguel A Brown, Ammar S Naqvi, Bo Zhang, Phillip B

Storm, … Jo Lynne Rokita *BMC Bioinformatics* (2020-12) https://doi.org/gm84mh DOI: 10.1186/s12859-020-03922-7 · PMID: 33317447 · PMCID: PMC7737294

22. **MSFragger: ultrafast and comprehensive peptide identification in mass spectrometry–based proteomics** Andy T Kong, Felipe V Leprevost, Dmitry M Avtonomov, Dattatreya Mellacheruvu, Alexey I Nesvizhskii *Nature Methods* (2017-04-10) https://doi.org/f9z6p7 DOI: 10.1038/nmeth.4256 · PMID: 28394336 · PMCID: PMC5409104

23. **Correcting systematic bias and instrument measurement drift with mzRefinery** Bryson C Gibbons, Matthew C Chambers, Matthew E Monroe, David L Tabb, Samuel H Payne *Bioinformatics* (2015-08-04) https://doi.org/gb5g57 DOI: 10.1093/bioinformatics/btv437 · PMID: 26243018 · PMCID: PMC4653383

24. **MS-GF+ makes progress towards a universal database search tool for proteomics** Sangtae Kim, Pavel A Pevzner *Nature Communications* (2014-10-31) https://doi.org/ggkdq8 DOI: 10.1038/ncomms6277 · PMID: 25358478 · PMCID: PMC5036525

25. **Spectral probabilities of top-down tandem mass spectra** Xiaowen Liu, Matthew W Segar, Shuai Cheng Li, Sangtae Kim *BMC Genomics* (2014-01) https://doi.org/gb3gzt DOI: 10.1186/1471-2164-15-s1-s9 · PMID: 24564718 · PMCID: PMC4046700

26. **A probability-based approach for high-throughput protein phosphorylation analysis and site localization** Sean A Beausoleil, Judit Villén, Scott A Gerber, John Rush, Steven P Gygi *Nature Biotechnology* (2006-09-10) https://doi.org/dbwqf4 DOI: 10.1038/nbt1240 · PMID: 16964243

27. **GISTIC2.0 facilitates sensitive and confident localization of the targets of focal somatic copy-number alteration in human cancers** Craig H Mermel, Steven E Schumacher,

Barbara Hill, Matthew L Meyerson, Rameen Beroukhim, Gad Getz *Genome Biology* (2011-04-28) https://doi.org/10.1186/gb-2011-12-4-r41 DOI: 10.1186/gb-2011-12-4-r41

28. **deconstructSigs: delineating mutational processes in single tumors distinguishes DNA repair deficiencies and patterns of carcinoma evolution** Rachel Rosenthal, Nicholas McGranahan, Javier Herrero, Barry S Taylor, Charles Swanton *Genome Biology* (2016-02-22) https://doi.org/f8bdsq DOI: 10.1186/s13059-016-0893-4 · PMID: 26899170 · PMCID: PMC4762164

29. **Signatures of mutational processes in human cancer**, Ludmil B Alexandrov, Serena Nik-Zainal, David C Wedge, Samuel AJR Aparicio, Sam Behjati, Andrew V Biankin, … Michael R Stratton *Nature* (2013-08-14) https://doi.org/f22m2q DOI: 10.1038/nature12477 · PMID: 23945592 · PMCID: PMC3776390

30. **Tumor mutational burden standardization initiatives: Recommendations for consistent tumor mutational burden assessment in clinical samples to guide immunotherapy treatment decisions** Albrecht Stenzinger, Jeffrey D Allen, Jörg Maas, Mark D Stewart, Diana M Merino, Madison M Wempe, Manfred Dietel *Genes, Chromosomes and Cancer* (2019-03-07) https://doi.org/ggc8pj DOI: 10.1002/gcc.22733 · PMID: 30664300 · PMCID: PMC6618007

31. **The 2021 WHO Classification of Tumors of the Central Nervous System: a summary** David N Louis, Arie Perry, Pieter Wesseling, Daniel J Brat, Ian A Cree, Dominique Figarella-Branger, Cynthia Hawkins, HK Ng, Stefan M Pfister, Guido Reifenberger, … David W Ellison *Neuro-Oncology* (2021-06-29) https://doi.org/gmqhbf DOI: 10.1093/neuonc/noab106 · PMID: 34185076 · PMCID: PMC8328013

32. **Alterations in ALK/ROS1/NTRK/MET drive a group of infantile hemispheric gliomas** Ana S Guerreiro Stucklin, Scott Ryall, Kohei Fukuoka, Michal Zapotocky, Alvaro Lassaletta, Christopher Li, Taylor Bridge, Byungjin Kim, Anthony Arnoldo, Paul E Kowalski, … Cynthia Hawkins *Nature Communications* (2019-09-25) https://doi.org/gh7bg8 DOI: 10.1038/s41467-019-12187-5 · PMID: 31554817 · PMCID: PMC6761184

33. **Molecular subgrouping of atypical teratoid/rhabdoid tumors—a reinvestigation and current consensus** Ben Ho, Pascal D Johann, Yura Grabovska, Mamy Jean De Dieu Andrianteranagna, Fupan Yao, Michael Frühwald, Martin Hasselblatt, Franck Bourdeaut, Daniel Williamson, Annie Huang, Marcel Kool *Neuro-Oncology* (2019-12-31) https://doi.org/gn3kcm DOI: 10.1093/neuonc/noz235 · PMID: 31889194 · PMCID: PMC7229260

34. **A transcriptome-based classifier to determine molecular subtypes in medulloblastoma** Komal S Rathi, Sherjeel Arif, Mateusz Koptyra, Ammar S Naqvi, Deanne M Taylor, Phillip B Storm, Adam C Resnick, Jo Lynne Rokita, Pichai Raman *PLOS Computational Biology* (2020-10-29) https://doi.org/gm84kq DOI: 10.1371/journal.pcbi.1008263 · PMID: 33119584 · PMCID: PMC7654754

35. **NGSCheckMate: software for validating sample identity in next-generation sequencing studies within and across data types** Sejoon Lee, Soohyun Lee, Scott Ouellette, Woong-Yang Park, Eunjung A Lee, Peter J Park *Nucleic Acids Research* (2017-03-23) https://doi.org/f9xrq4 DOI: 10.1093/nar/gkx193 · PMID: 28369524 · PMCID: PMC5499645

36. **Somalier: rapid relatedness estimation for cancer and germline studies using efficient genome sketches** Brent S Pedersen, Preetida J Bhetariya, Joe Brown, Stephanie N Kravitz, Gabor Marth, Randy L Jensen, Mary P Bronner, Hunter R Underhill, Aaron R Quinlan *Genome Medicine* (2020-07-14) https://doi.org/gtsm62 DOI: 10.1186/s13073-020-00761-2 · PMID: 32664994 · PMCID: PMC7362544

37. **dbGaP Study** https://www.ncbi.nlm.nih.gov/projects/gap/cgi-bin/study.cgi?study_id=phs000218.v23.p8

38. **dbGaP Study** https://www.ncbi.nlm.nih.gov/projects/gap/cgi-bin/study.cgi?study_id=phs001436.v1.p1

39. **dbGaP Study** https://www.ncbi.nlm.nih.gov/projects/gap/cgi-bin/study.cgi?study_id=phs002517.v4.p2

40. **dbGaP Study** https://www.ncbi.nlm.nih.gov/projects/gap/cgi-bin/study.cgi?study_id=phs000178.v11.p8

Table S1

Click here to access/download
Supplementary Material
SuppTable1-Histologies.xlsx

Table S2

Click here to access/download
Supplementary Material
SuppTable2-Molecular-Subtype-Table.xlsx

Table S3

Click here to access/download
Supplementary Material
SuppTable3-List_Package_Table.xlsx

Table S4

Click here to access/download
Supplementary Material
SuppTable4-dna-rna-qc.xlsx

Personal Cover
Click here to access/download;Personal Cover;Geng_OpenPedCan_cover.pdf

Children's National

Children's National Hospital
111 Michigan Ave NW
Washington, DC 20010-2916
ChildrensNational.org

February 28, 2025

Editorial Office
GigaScience

Subject: Submission of Data Note – The Open Pediatric Cancer Project

Dear Editors,

I am pleased to submit our manuscript, entitled **"The Open Pediatric Cancer Project"**, for consideration as a Data Note in *GigaScience*. This manuscript presents comprehensive genomic and clinical data from 6,112 pediatric cancer patients with 7,096 tumor events across more than 100 histologies as a new resource called the Open Pediatric Cancer Project (*OpenPedCan*).

In *OpenPedCan*, we expand our recently published OpenPBTA study to co-harmonize several additional studies (TARGET, GMKF, GTEx, TCGA), allowing cross-cohort integration of genomic, transcriptomic, proteomic, methylation, and clinical datasets from large-scale pediatric cancer studies, enabling cross-study analyses, biomarker discovery, and translational research. We provide scalable analysis modules that support standardized data sharing, reproducibility, and accessibility. *OpenPedCan* is fully aligned with FAIR (Findable, Accessible, Interoperable, Reusable) principles and is intended to facilitate collaborative research in the pediatric oncology community.

We believe this dataset will be of interest to researchers in cancer genomics, computational biology, and precision medicine, particularly those focused on rare and pediatric malignancies. In accordance with *GigaScience*'s commitment to open data, we have made all data, metadata, and analytical workflows publicly available in GitHub and CAVATICA.

We confirm that this manuscript has not been published elsewhere and is not under consideration by another journal. All authors have approved this submission, and we have no conflicts of interest to disclose.

Thank you for considering our submission. We look forward to your feedback and the opportunity to contribute to *GigaScience's* growing repository of high-impact open data resources.

Sincerely,

Jo Lynne Rokita, Ph.D.
Data Science Faculty, Center for Cancer and Immunology Research
Director, Brain Tumor Institute Bioinformatics Core
Children's National Hospital
Assistant Professor of Pediatrics
The George Washington University School of Medicine and Health Sciences
