# Supplementary material for: The Open Pediatric Cancer Project
Source: Gigascience. 2025 Sep 2;14:giaf093. doi: 10.1093/gigascience/giaf093 (PMC12402770; doi:10.1093/gigascience/giaf093)
Supplement: giaf093_Authors_Response_To_Reviewer_Comments_Original_Submission [file giaf093_authors_response_to_reviewer_comments_original_submission.pdf]

Author's Response To Reviewer Comments

The due date for submitting the revised version of your article is 07 Jul 2025.

Dear Dr. Rokita,

Your manuscript "The Open Pediatric Cancer Project" (GIGA-D-25-00086) has been assessed by our reviewers. Although it is of interest, we are unable to consider it for publication in its current form. The reviewers have raised a number of points which we believe would improve the manuscript and may allow a revised version to be published in GigaScience.

Their reports are below.

As you will see, the reviewers have quite different opinions regarding the overall merit of the submission and reviewer #2 is rather critical regarding the advance presented. However, considering our criteria for a "Data Note" submission, I agree with reviewer #1 that a revised submission may be suitable for publication under this article type. Gigascience Data Notes do not need to present new research insights, but they need to present an advance in the sense that the article describes a widely useful, reproducible, large data set).

With this in mind, I recommend to improve the manuscript by showing clearer how the work presents an advance in the field - e.g. by following the advice of reviewer#1 to present more information on validation and quality control.

Please also include a section on ethics and consent, mentioning relevant ethics approvals by IRBs and a declaration on written informed (parental) consent. Please also describe the different access levels of the data (public domain vs controlled access etc) and describe how the framework handles access to controlled data (see the comment of reviewer #2).

Regarding the comment of reviewer #1 to include URLs directly in the paper: According to journal style, URLs should go to the bibliography - we treat internet sources as citable objects, so you may ignore this comment.

Regarding archiving the github repo: Usually our curators take care of this this prior to publication, so you can skip this for now.

Editorial Summary and Required Revisions:
We appreciate the editor’s guidance and agree that this revised submission is best suited for the "Data Note" format. In response, we have:
Clarified the advance of OpenPedCan over OpenPBTA, emphasizing increased scope, novel data types, and reuse potential.
Expanded the Data Validation and Quality Control section with detailed benchmarking and concordance metrics.
Added a new section on ethics and consent and describe data access and general research use consents.
Added a new table detailing public vs. controlled access data and the framework’s compliance.
Retained URL citations in the bibliography per journal style and clarified Zenodo archiving strategy.

Reviewer reports:

Reviewer #1:
I love this type of work. This research will be invaluable to the wider research community of people studying pediatric cancers. It will save lots of time and frustration and move the field forward. The paper is well written. I have to admit that I am not well versed in all of the latest software tools and settings to use for processing all of the data types that the repository includes. So I cannot vouch for or against those. However, the tools that I am familiar with seem reasonable. I have a few comments / suggestions / questions.

* How is patient privacy maintained? Sorry if I missed this. The paper mentions the original sources of the data. However, if I understand correctly, OpenPBTA has reprocessed versions of the data. What processes are used to

regulate access to versions of the data that must be kept secure? Perhaps I am misunderstanding the ideas behind how this works.

We appreciate the reviewer's important question regarding patient privacy and data security. To clarify, in our previous work (OpenPBTA) and this project (OpenPedCan), we only include reprocessed and harmonized data derived from sources that are explicitly allowed as open-access/general research use, designated by the original data contributors and data custodians (e.g., dbGAP, Kids First, CBTN, GTEx, and TCGA). Controlled-access datasets (e.g., raw FASTQs/BAMs/CRAMs) are never distributed through OpenPedCan. We obtained de-identified data and no protected health information (PHI) or identifiable data elements are present in the data we distribute through OpenPedCan.

For datasets originally subject to controlled access (e.g., raw sequencing files or individual-level protected clinical metadata), we only incorporate derived, de-identified summary-level outputs that have been approved for public release in OpenPedCan. All processing and harmonization steps occured on the secure CAVATICA platform, with appropriate data use agreements and NIH user authentication in place. This has been clarified in the revised manuscript (Methods and new Ethics and Consent Statement section).

* Validation. It would be helpful if the paper could touch on the approach the authors use to ensure that data that they have (re)processed are valid. For example, are there any known findings that show up after the data have been reprocessed? Or are there other ways of assessing quality?

To ensure the validity and reproducibility of all data presented in OpenPedCan, we implemented rigorous, multi-layered quality control and benchmarking. All RNA-seq and WGS samples passed minimum quality thresholds for coverage and read metrics and underwent sample identity checks using NGSCheckMate and Somalier (Data Validation and Quality Control and Supplemental Table 4). Our molecular subtyping modules recover hallmark genomic and transcriptomic events known in pediatric CNS tumors, including detection of KIAA1549::BRAF fusions in low-grade gliomas, H3 K28 mutations in diffuse midline gliomas, H3 G35 mutations in diffuse hemispheric gliomas, TP53 mutations in high-grade gliomas, and MYCN amplification in neuroblastoma, consistent with prior literature. These subtyping modules were fully version-controlled and subjected to independent code and scientific review. In instances of molecular alterations which did not match an initial pathology diagnosis, we conferred with molecular pathologists to re-review slides and molecular data. In rare cases, the integrated diagnosis has changed due to these findings. This is all documented within our molecular-subtype-pathology module. In medulloblastoma, for instance, we observe a near 100% concordance between RNA-based and methylation-based subtype calls (included in a new Table 1). All workflows are containerized, publicly available, and have been reused by the community (as evidenced by Zenodo downloads, GitHub forks, and cited publications). These validations ensure that OpenPedCan is both technically robust and biologically informative, meeting the goals of a reproducible and reusable community resource.

* The paper mentions TCGA and GTex. It also mentions that adult data are included. But I didn't see a clear rationale for doing this.
We thank the reviewer for this question and welcome the opportunity to clarify our rationale for including adult data from TCGA and GTEx. One of the goals of the OpenPedCan project is to enable interrogation of gene expression patterns that are tumor-specific and/or pediatric-specific. To do this effectively, comparative reference datasets are essential. Since the developmental GTEx sequencing data does not yet exist, we include adult tissue data from GTEx and adult tumor data from TCGA as interim reference points. These harmonized datasets allow users to evaluate whether a gene is aberrantly expressed in pediatric tumors relative to normal adult tissues (GTEx) or is enriched in pediatric tumors relative to adult cancers (TCGA). This type of analysis is frequently used in drug target discovery, particularly in the immunotherapy space, where distinguishing tumor-specific expression from normal tissue expression is critical for minimizing off-target effects.

We have updated the manuscript to clarify this rationale and emphasize the importance of including these adult datasets until developmentally-matched normal references become available.

* The paper includes many links, some of which reference portions of the GitHub site. It would be best to display the URLs in the paper itself. It would also be useful to reference a Zenodo-archived version of the GitHub site so that there is a versioned record of the repository at the time of submission.

Per the editor's suggestion, we will keep URLs in the bibliography. Additionally, we agree with this comment about Zenodo. We currently provide a zenodo-archived versions of the analysis repository with every release (https://zenodo.org/search?q=parent.id%3A6473912&f=allversions%3Atrue&l=list&p=1&s=10&sort=version), as well as the manuscript repository (https://zenodo.org/search?q=parent.id%3A13835293&f=allversions%3Atrue&l=list&p=1&s=10&sort=version). We have now added the zenodo source code URL for the latest release, https://zenodo.org/records/15750097, to Availability of source code and requirements section of the manuscript.

* Supplementary Table 1 has a tab with information about the patient metadata ("Biospecimen-level metadata and clinical data"). However, I didn't see details in the paper about how these were harmonized. How did the authors ensure that the metadata values come from disparate sources were used consistently? What expertise did they have? How did they resolve inconsistencies or missing data? Supplementary Table 1 indicates a definition and a data type for each of these fields. It would be much more useful to provide ontology term(s) for each of these fields so that the metadata were machine readable.

We thank the reviewer for this insightful comment and the opportunity to clarify. The biospecimen- and patient-level metadata used in the OpenPedCan project were harmonized through a collaborative effort involving analysts, data engineers, and domain experts from the Children's Brain Tumor Network (CBTN), the Gabriella Miller Kids First Data Resource Center (Kids First DRC), and the OpenPedCan team. These groups, led in part by co-authors Dr. Rokita, Dr. Deanne Taylor, and Dr. Adam Resnick, bring together decades of experience in developing and implementing pediatric-specific data models and ontologies.

To ensure consistency across disparate sources, metadata harmonization followed standardized pipelines and protocols established by the Kids First DRC, which are informed by best practices in biomedical informatics and ontology-based modeling. Key steps in this process included:
Source verification and controlled vocabulary mapping: Metadata fields were reviewed against source documentation, and values were mapped to standardized vocabularies and ontologies (e.g., NCIT, MONDO, and EFO) whenever possible.
Automated and manual QC: Quality control steps included automated validation (e.g., datatype enforcement, missing value checks) and manual review by clinical data experts to resolve ambiguities or inconsistencies.
Consensus curation: In cases of discordant or incomplete information, cross-referencing across multiple fields (e.g., diagnosis, primary site, morphology) and iterative expert curation were used to assign the most accurate and biologically meaningful labels.
We have now added a new sub-section called Clinical data harmonization and updated Supplementary Table 1 to include ontology terms from NCIT, MONDO, and/or EFO for each diagnosis to enhance machine readability and facilitate integration with external tools and databases. These updates are intended to improve data interoperability and reuse, in line with FAIR data principles.

============

Reviewer #2:

Shapiro et al. describe the Open Pediatric Cancer Project, a dataset, web portals, and a Github repository to facilitate data access, analysis, and encourage collaborations using pediatric cancer omics data. While the concept is inspired, it does not constitute a significant advance over the previously described OpenPBTA project. The goal of the manuscript may be to provide a pointer to the updated datasets and web resources, but this does not seem like a sufficient reason to publish. As far as I can tell, all of the information in the manuscript is already provided on the OpenPedCan Bioportal (which is really useful, to be fair) and on GitHub. To publish a manuscript just as a pointer to that information does not seem justifiable in my opinion.

Major Concerns:

1. Novelty and Validity of Key Features:

The manuscript highlights several key features of OpenPedCan, including data harmonization, multi-omic integration, reproducibility, scalability, versioned data releases, accessibility, alignment with WHO 2021

classifications, and the open-source framework. However, these features are not novel. Many of them represent standard practices in the field. Moreover, some claims appear questionable:
* Reproducibility: While the authors claim reproducibility, using OpenPedCan's dockerized workflows would require significant computational resources (e.g., 98GB of CPU) or expensive cloud services (e.g., AWS).

We thank the reviewer for this important observation and the opportunity to clarify. While we agree that reproducibility is now a widely expected standard in the field, our emphasis on it reflects our commitment to transparency and usability across diverse research environments. All OpenPedCan workflows are fully open-source, version-controlled, and containerized using Docker and CWL, enabling reproducibility across both local and cloud-based platforms.

In response to the reviewer's concern regarding computational requirements: although some of the full-cohort analyses are resource-intensive, none of the OpenPedCan modules require 98 GB of CPU memory. In practice, the majority of modules can be run on local machines or HPCs with 16–64 GB of RAM, or via cost-efficient cloud options. For example, the most computationally demanding module "Analysis pre-release" involves full pan-cohort reruns for new data releases, requiring an m6i.4xlarge AWS EC2 instance (64 GB RAM, 16 vCPUs), which costs approximately $0.768/hour. This most memory-intensive module generally completes in ~80 minutes, resulting in a total cost of less than $2. We have now added this to the Availability of source code and requirements section.

Importantly, the memory-intensive modules are typically such because of the extraordinary number of samples in this dataset. If users would like to run these modules on their own data, they are typically not memory intensive.

Additionally, upstream harmonized data and workflows are available in the CAVATICA platform, further reducing the barrier to entry by enabling researchers to run specific analyses without repeating the full pipeline.

* Accessibility: The platform's interface requires users to have a Gmail account, limiting its accessibility. Alternative login options should be considered.
We thank the reviewer for this important feedback regarding accessibility. While it is true that the PedcBioPortal interface currently requires a Gmail account for access, we would like to emphasize that this is only one of several access points for OpenPedCan data. To ensure broad and equitable access, the data and results are made available through multiple platforms, most of which do not require a Gmail account. We have now added a table to the manuscript (Table 2) summarizing the access methods, data types, and any associated requirements.

* Open-Source Framework: The manuscript does not adequately address how the framework handles access to controlled data, such as those integrated from external sources like TARGET and TCGA, which may require restricted access permissions.
We thank the reviewer for raising this important point. We would like to clarify that the OpenPedCan framework exclusively utilizes open-access data, including that from external resources such as TARGET and TCGA. Only publicly available files (e.g., processed expression matrices, mutation annotation format (MAF) files, and clinical summary data) are integrated into OpenPedCan analyses. No controlled-access data, such as raw sequencing data, are provided within OpenPedCan or included in any release materials. Therefore, no special access permissions or dbGaP approvals are required. We have updated the manuscript to explicitly state that only open-access components of external datasets were included, ensuring full reproducibility and compliance with data use restrictions (Ethics and Consent Statement section).

2. Lack of Novel Methodologies and Findings:

* While OpenPedCan integrates data from existing workflows and portals (e.g., Gabriella Miller Kids First, TCGA), the manuscript does not clearly outline novel methodologies or scientific contributions. Most prominently, the submission appears to be an incremental extension of the previous manuscript describing OpenPBTA published in Cell Genomics 2023.
The only potentially novel components appear to be proteomics and molecular subtyping based on methylation, but no specific examples or case studies demonstrating the novelty or impact of these contributions are

provided.
We thank the reviewer for the opportunity to clarify the significant contributions of OpenPedCan beyond the initial OpenPBTA manuscript. We also agree with the editor that a “Data Note” format need not introduce novel analytical methods, and that value lies in the reproducibility, scale, and usability of the dataset. While OpenPedCan builds upon the OpenPBTA foundation, it represents a significant expansion in both scope and scale, as well as in methodological integration.
1. Dramatic Expansion in Cohort Size and Scope:
OpenPBTA included 943 patients with 1,074 primary brain tumors and 22 patient-derived cell lines. In contrast, OpenPedCan expands the dataset to over 6,000 patients, encompassing 7,096 tumors and more than 48,000 biospecimens, across dozens of pediatric cancer types beyond CNS tumors. This scale substantially increases the statistical power and breadth of analyses available to researchers.

2. Multi-Modal Data Integration Beyond OpenPBTA:
OpenPedCan introduces new data modalities not present in OpenPBTA, including:
Methylation array data with harmonized molecular subtype annotations
miRNA expression data
Harmonized GTEx and TCGA datasets for comparative analyses
Proteomics data from CPTAC for pediatric solid tumors.
These additions required the development of new workflows and data models to integrate across platforms and modalities.

3. Major Annotation and Workflow Enhancements:
All RNA and DNA samples have been re-annotated from GENCODE v27 to v39, which improves transcript model consistency across analyses. This update involved substantial realignment of feature-level annotations and necessitated changes in upstream workflows and downstream interpretations, including the reclassification of fusion events and transcript-level expression. Mention of this update was omitted from the manuscript submission, but we have now added this information to the Data Description and Methods section.

4. Infrastructure and Community Reuse:
OpenPedCan introduces version-controlled, modularized pipelines with support for continuous data updates and downstream reuse. The workflows have been leveraged by external NIH efforts (e.g., Molecular Targets Platform) and by the pediatric cancer research community at large.

We have revised the manuscript to expand the Re-use potential section to demonstrate how additional data types—such as proteomics and methylation-based subtypes—enable new biological insights that were not possible with OpenPBTA alone.

3. Redundancy with Existing Tools:

* The manuscript states that OpenPedCan serves as a community resource for addressing research questions and providing orthogonal validation datasets. However, there is nothing presented in OpenPedCan that cannot already be achieved with existing tools. This makes the claim somewhat redundant, as the platform largely serves as a data integrator rather than offering unique capabilities.
We appreciate the reviewer’s perspective and welcome the opportunity to clarify the unique value OpenPedCan brings as a shared, multi-institutional resource. While OpenPedCan builds upon existing datasets and tools, its core contribution lies in the scale, depth, and accessibility of its harmonized multi-modal pediatric cancer dataset, which would be cost-prohibitive and technically challenging for individual labs to recreate.

OpenPedCan is the result of a multi-million dollar, multi-year effort involving the Children’s Hospital of Philadelphia, the Gabriella Miller Kids First Data Resource Center, and other NIH-supported programs. The project integrates over 48,000 biospecimens from more than 6,000 patients, covering dozens of pediatric cancer types and incorporating genomics, transcriptomics, epigenomics, and proteomics. Achieving this required extensive cross-consortia collaboration, infrastructure investment, and coordinated data modeling, all of which are provided openly and reproducibly to the research community.

Reproducing this level of harmonization and integration at the lab level would cost millions of dollars in compute time and personnel, not including the overhead of aligning disparate data types, annotating metadata with

controlled vocabularies, or maintaining workflows. OpenPedCan absorbs that burden—freeing individual labs to focus on discovery rather than infrastructure.

While our platform integrates existing tools, it enables community-scale reuse, orthogonal validation, and therapeutic discovery that would otherwise be inaccessible to most research groups. We have clarified this impact in the revised manuscript and expanded on how OpenPedCan is actively used in projects such as the Molecular Targets Platform.

Minor Concerns:

1. Splicing Analysis Module:
* The manuscript refers to a splicing analysis module (Figure 2: OpenPedCan Analysis Workflow), but there is no further description or discussion of this module within the text. Further elaboration is needed.
We thank the reviewer for pointing this out. We would like to clarify that splicing is a new data modality included in the OpenPedCan dataset, rather than an analysis module within the repository. In the current release, splicing data are represented as junction counts and splicing events generated using the rMATS algorithm, data which did not exist during the OpenPBTA project. We have now released them into OpenPedCan for the research community.

To avoid confusion, we have updated the manuscript into two sections delineating the upstream workflows (Section called “Primary Workflows through Kids First”) and downstream workflows (Section called “Creation of OpenPedCan Analysis modules”). We have further expanded the Splicing quantification sub-section.

2. Incomplete Module Descriptions:
* The manuscript describes several analysis modules, but it should provide more comprehensive descriptions of the analysis modules, especially the Splicing Analysis module.
* Additionally, the Molecular Subtyping component, based on molecular and methylation data, is the only module with a clear methodological explanation.
* Further clarification on the methods used in other modules would be beneficial.
We thank the reviewer for this valuable feedback. While many of these modules were carried over from OpenPBTA, we have created substantial updates to modules and created new modules. In response, we have revised the manuscript to provide more detailed descriptions of each analysis module.

Specifically:
We expanded all previously sparse module descriptions in the Methods section and added a new Supplemental Table 2 to clarify the analytical approaches, tools used, input data types, and outputs for each module.
We refined the description of the Molecular Subtyping module, which involves methylation-based classification using publicly available classifiers and supervised learning approaches.
In addition, we added a new Clinical Harmonization section to describe our approach for curating and integrating biospecimen- and patient-level metadata—per the helpful suggestion of Reviewer 1.
These updates ensure that each analytical component of OpenPedCan is now described with clarity and completeness, and we hope they address the reviewer’s concerns.
