# Supplementary material for: The Open Pediatric Cancer Project
Source: Gigascience. 2025 Sep 2;14:giaf093. doi: 10.1093/gigascience/giaf093 (PMC12402770; doi:10.1093/gigascience/giaf093)
Supplement: giaf093_Authors_Response_To_Reviewer_Comments_Revision_1 [file giaf093_authors_response_to_reviewer_comments_revision_1.pdf]

Author's Response To Reviewer Comments

We have made editorial revisions which include:
- Removing URLs from within text and citing data, repositories, and code.
- Added the zenodo repository to the bibliography as a citation.
- Updating the license in the manuscript to CC0-BSD3.
- Updated Table 2 to include all accessions for dbGAP and portals with processed data for accessibility.

Additionally, I have added one funding source which was initially omitted, the NIH U24.
