# Supplementary material for: The Open Pediatric Cancer Project
Source: Gigascience. 2025 Sep 2;14:giaf093. doi: 10.1093/gigascience/giaf093 (PMC12402770; doi:10.1093/gigascience/giaf093)
Supplement: giaf093_Reviewer_1_Report_Original_Submission — Stephen R Piccolo, Ph.D. -- 3/28/2025 [file giaf093_reviewer_1_report_original_submission.pdf]

Reviewer Report

Title: The Open Pediatric Cancer Project

Version: Original Submission **Date:** 3/28/2025

Reviewer name: Stephen R Piccolo, Ph.D.

Reviewer Comments to Author:

I love this type of work. This research will be invaluable to the wider research community of people studying pediatric cancers. It will save lots of time and frustration and move the field forward. The paper is well written. I have to admit that I am not well versed in all of the latest software tools and settings to use for processing all of the data types that the repository includes. So I cannot vouch for or against those. However, the tools that I am familiar with seem reasonable. I have a few comments / suggestions / questions.* How is patient privacy maintained? Sorry if I missed this. The paper mentions the original sources of the data. However, if I understand correctly, OpenPBTA has reprocessed versions of the data. What processes are used to regulate access to versions of the data that must be kept secure? Perhaps I am misunderstanding the ideas behind how this works.* Validation. It would be helpful if the paper could touch on the approach the authors use to ensure that data that they have (re)processed are valid. For example, are there any known findings that show up after the data have been reprocessed? Or are there other ways of assessing quality?* The paper mentions TCGA and GTex. It also mentions that adult data are included. But I didn't see a clear rationale for doing this.* The paper includes many links, some of which reference portions of the GitHub site. It would be best to display the URLs in the paper itself. It would also be useful to reference a Zenodo-archived version of the GitHub site so that there is a versioned record of the repository at the time of submission.* Supplementary Table 1 has a tab with information about the patient metadata ("Biospecimen-level metadata and clinical data"). However, I didn't see details in the paper about how these were harmonized. How did the authors ensure that the metadata values come from disparate sources were used consistently? What expertise did they have? How did they resolve inconsistencies or missing data? Supplementary Table 1 indicates a definition and a data type for each of these fields. It would be much more useful to provide ontology term(s) for each of these fields so that the metadata were machine readable.

Level of Interest

Please indicate how interesting you found the manuscript: Choose an item.

Quality of Written English

Please indicate the quality of language in the manuscript: Choose an item.

Declaration of Competing Interests

Please complete a declaration of competing interests, considering the following questions:

- Have you in the past five years received reimbursements, fees, funding, or salary from an organisation that may in any way gain or lose financially from the publication of this manuscript, either now or in the future?
- Do you hold any stocks or shares in an organisation that may in any way gain or lose financially from the publication of this manuscript, either now or in the future?
- Do you hold or are you currently applying for any patents relating to the content of the manuscript?
- Have you received reimbursements, fees, funding, or salary from an organization that holds or has applied for patents relating to the content of the manuscript?
- Do you have any other financial competing interests?
- Do you have any non-financial competing interests in relation to this paper?

If you can answer no to all of the above, write 'I declare that I have no competing interests' below. If your reply is yes to any, please give details below.

I declare that I have no competing interestes

I agree to the open peer review policy of the journal. I understand that my name will be included on my report to the authors and, if the manuscript is accepted for publication, my named report including any attachments I upload will be posted on the website along with the authors' responses. I agree for my report to be made available under an Open Access Creative Commons CC-BY license (http://creativecommons.org/licenses/by/4.0/). I understand that any comments which I do not wish to be included in my named report can be included as confidential comments to the editors, which will not be published.

Choose an item.

To further support our reviewers, we have joined with Publons, where you can gain additional credit to further highlight your hard work (see: https://publons.com/journal/530/gigascience). On publication of this paper, your review will be automatically added to Publons, you can then choose whether or not to claim your Publons credit. I understand this statement.

Yes Choose an item.
