# Supplementary material for: The Open Pediatric Cancer Project
Source: Gigascience. 2025 Sep 2;14:giaf093. doi: 10.1093/gigascience/giaf093 (PMC12402770; doi:10.1093/gigascience/giaf093)
Supplement: giaf093_Reviewer_2_Report_Original_Submission — Jacek Majewski -- 4/7/2025 [file giaf093_reviewer_2_report_original_submission.pdf]

Reviewer Report

Title: The Open Pediatric Cancer Project

Version: Original Submission **Date:** 4/7/2025

Reviewer name: Jacek Majewski

Reviewer Comments to Author:

Shapiro et al. describe the Open Pediatric Cancer Project, a dataset, web portals, and a Github repository to facilitate data access, analysis, and encourage collaborations using pediatric cancer omics data. While the concept is inspired, it does not constitute a significant advance over the previously described OpenPBTA project. The goal of the manuscript may be to provide a pointer to the updated datasets and web resources, but this does not seem like a sufficient reason to publish. As far as I can tell, all of the information in the manuscript is already provided on the OpenPedCan Bioportal (which is really useful, to be fair) and on GitHub. To publish a manuscript just as a pointer to that information does not seem justifiable in my opinion.Major Concerns:1. Novelty and Validity of Key Features:The manuscript highlights several key features of OpenPedCan, including data harmonization, multi-omic integration, reproducibility, scalability, versioned data releases, accessibility, alignment with WHO 2021 classifications, and the open-source framework. However, these features are not novel. Many of them represent standard practices in the field. Moreover, some claims appear questionable:* Reproducibility: While the authors claim reproducibility, using OpenPedCan's dockerized workflows would require significant computational resources (e.g., 98GB of CPU) or expensive cloud services (e.g., AWS).* Accessibility: The platform's interface requires users to have a Gmail account, limiting its accessibility. Alternative login options should be considered.* Open-Source Framework: The manuscript does not adequately address how the framework handles access to controlled data, such as those integrated from external sources like TARGET and TCGA, which may require restricted access permissions.2. Lack of Novel Methodologies and Findings:* While OpenPedCan integrates data from existing workflows and portals (e.g., Gabriella Miller Kids First, TCGA), the manuscript does not clearly outline novel methodologies or scientific contributions. Most prominently, the submission appears to be an incremental extension of the previous manuscript describing OpenPBTA published in Cell Genomics 2023.The only potentially novel components appear to be proteomics and molecular subtyping based on methylation, but no specific examples or case studies demonstrating the novelty or impact of these contributions are provided.3. Redundancy with Existing Tools:* The manuscript states that OpenPedCan serves as a community resource for addressing research questions and providing orthogonal validation datasets. However, there is nothing presented in OpenPedCan that cannot already be achieved with existing tools. This makes the claim somewhat redundant, as the platform largely serves as a data integrator rather than offering unique capabilities.Minor Concerns:1. Splicing Analysis Module:* The manuscript refers to a splicing analysis module (Figure 2: OpenPedCan Analysis Workflow), but there is no further description or discussion of this module within the text. Further elaboration is needed.2. Incomplete Module Descriptions:* The manuscript describes several analysis modules, but it should provide more comprehensive descriptions of the analysis modules, especially the Splicing Analysis

module.* Additionally, the Molecular Subtyping component, based on molecular and methylation data, is the only module with a clear methodological explanation.* Further clarification on the methods used in other modules would be beneficial.

Level of Interest

Please indicate how interesting you found the manuscript: Choose an item.

Quality of Written English

Please indicate the quality of language in the manuscript: Choose an item.

Declaration of Competing Interests

Please complete a declaration of competing interests, considering the following questions:

- Have you in the past five years received reimbursements, fees, funding, or salary from an organisation that may in any way gain or lose financially from the publication of this manuscript, either now or in the future?
- Do you hold any stocks or shares in an organisation that may in any way gain or lose financially from the publication of this manuscript, either now or in the future?
- Do you hold or are you currently applying for any patents relating to the content of the manuscript?
- Have you received reimbursements, fees, funding, or salary from an organization that holds or has applied for patents relating to the content of the manuscript?
- Do you have any other financial competing interests?
- Do you have any non-financial competing interests in relation to this paper?

If you can answer no to all of the above, write 'I declare that I have no competing interests' below. If your reply is yes to any, please give details below.

I declare that I have no competing interests

I agree to the open peer review policy of the journal. I understand that my name will be included on my report to the authors and, if the manuscript is accepted for publication, my named report including any attachments I upload will be posted on the website along with the authors' responses. I agree for my report to be made available under an Open Access Creative Commons CC-BY license (http://creativecommons.org/licenses/by/4.0/). I understand that any comments which I do not wish to be included in my named report can be included as confidential comments to the editors, which will not be published.

Choose an item.

To further support our reviewers, we have joined with Publons, where you can gain additional credit to further highlight your hard work (see: https://publons.com/journal/530/gigascience). On publication of this paper, your review will be automatically added to Publons, you can then choose whether or not to claim your Publons credit. I understand this statement.

Yes Choose an item.
